# The transcription factor HHEX maintains glucocorticoid levels and protects adrenals from androgen-induced lipid depletion

**DOI:** 10.21203/rs.3.rs-6248794/v1

**Published:** 2025-04-15

**Authors:** Typhanie Dumontet, Kaitlin J. Basham, Micah C. Foster, Emma Silverman, Kyle A. Heard, Dominque Johnson, Chaelin Lee, Samuel W. Plaska, David T. Breault, David Penton, Felix Beuschlein, Adina F. Turcu, Christopher R. LaPensee, Antonio Marcondes Lerario, Gary D. Hammer

**Affiliations:** a Training Program in Organogenesis, Center for Cell Plasticity and Organ Design, University of Michigan, Ann Arbor, Michigan, USA; b Department of Internal Medicine, Division of Metabolism, Endocrinology, and Diabetes, University of Michigan, Ann Arbor, Michigan, USA; c Department of Oncological Sciences, Huntsman Cancer Institute, University of Utah, Salt Lake City, Utah, USA; d Department of Molecular and Integrative Physiology, University of Michigan, Ann Arbor, USA; e Division of Endocrinology, Boston Children’s Hospital, Boston, Massachusetts, USA.; f Electrophysiology Facility, University of Zurich, Switzerland; g Department of Endocrinology, Diabetology and Clinical Nutrition, University Hospital of Zurich (USZ) and University of Zurich (UZH), Zurich, Switzerland; h Medizinische Klinik und Poliklinik IV, Klinikum der Universität, Ludwig-Maximilians-Universität, Munich, Germany; i The LOOP Zurich - Medical Research Center, Zurich, Switzerland; j Department of Cell and Molecular Biology, University of Michigan, Ann Arbor, Michigan, United States; k Endocrine Oncology Program, Rogel Cancer Center, University of Michigan, Ann Arbor, Michigan, United States

**Keywords:** Steroidogenic lineage, PRH, zona fasciculata, corticosteroid, cholesterol, endocrine, autophagy, lipophagy, lipid droplet, testosterone, sexual dimorphism, ACTH, stress response, HPA axis

## Abstract

Glucocorticoid-producing cells of the adrenal cortex (*i.e*. zona fasciculata, zF) constitute the critical effectors of the hypothalamic-pituitary-adrenal axis, mediating the mammalian stress response. With glucocorticoids being essential for life, it is not surprising that zF dysfunction perturbs multiple organs that participate in optimizing cardiometabolic fitness. The zF forms a dynamic and heterogenous cell population endowed with the capacity to remodel through the engagement of both proliferative and differentiation programs that enable the adrenal to adapt and respond to diverse stressors. However, the mechanisms that sustain such differential responsiveness remain poorly understood. In this study, we resolved the transcriptome of the steroidogenic lineage by scRNA-seq using *Sf1-Cre; Rosa*^*mT/mG*^ reporter mice. We identified HHEX, a homeodomain protein, as the most enriched transcription factor in glucocorticoid-producing cells. We developed new genetic mouse models to demonstrate that HHEX deletion causes glucocorticoid insufficiency in male animals. Molecularly, we demonstrated that HHEX is an androgen receptor (AR) target gene, shaping the sexual dimorphism of the adrenal gland by repressing the female transcriptional program at puberty, while also maintaining zF cholesterol ester content by protecting lipid droplets from androgen-induced-lipophagy. Moreover, our study revealed that, in both sexes, HHEX is crucial for maintaining the identity of the innermost adrenocortical cell subpopulation. Specifically, loss of HHEX impairs the expression of *Abcb1b* (P-glycoprotein/MDR1), an efflux pump regulating steroid export and cellular levels of xenobiotics. Together, these data demonstrate that HHEX serves as a multi-functional regulator of post-natal adrenal maturation that is potentiated by androgens.

## In brief

In this study, we defined HHEX as a key regulator of adrenocortical cell function. By using multiple transgenic mouse models, we demonstrate that HHEX shapes AR-dependent sexual dimorphism in the zF following the onset of puberty. HHEX protects lipid droplets integrity from lipophagy induced by AR signaling and its absence results in glucocorticoid insufficiency in males. Additionally, in both sexes, HHEX supports the inner zF transcriptome, including the expression of *Abcb1b*, a critical regulator of intracellular levels of steroids and xenobiotics.

## INTRODUCTION

Glucocorticoids (GC) are cholesterol-derived steroid hormones widely known for maintaining glucose homeostasis^1^, modulating the immune system^2^, and orchestrating the circadian rhythm established by the suprachiasmatic nucleus^3^. Cortisol in humans, and corticosterone in rodents, hold central importance for the body’s response to stress by promoting energy mobilization. GC also play a key role in restoring body homeostasis after stress exposure by providing the appropriate inhibitory feedback responses to the hypothalamus and the pituitary gland. However, an imbalance in GC production is a characteristic of chronic stress exposure and is associated with depression, cognitive dysfunction^4^, susceptibility to infections^5^, elevated cardiometabolic morbidity, and increased mortality^6^.

To ultimately exploit biological vulnerabilities and develop targeted therapy for GC-associated disorders, it is essential to understand the mechanisms underlying the coordinated function and responsiveness of the GC-producing cells that reside within the adrenal gland. The *zona fasciculata (zF)* is an inner concentric and circumferential region comprising up to 80% of the adrenal cortex in humans^7^. It is characterized by a high content of the steroid precursor, cholesterol, and the expression of *CYP11B1* that encodes the enzyme required for the final step of GC synthesis^8–13^. At the molecular level, the elevation of circulating ACTH during stress exposure drives adrenal GC production by mobilizing cholesterol stored in lipid droplets in the form of cholesterol esters. The metabolic conversion of free cholesterol moieties into steroid hormones is accomplished by increasing steroidogenic enzyme expression and activity in the mitochondria of zF cells^14,15^.

Historically, the zF has been defined as a homogeneous cell population based on the expression of CYP11B1 in all zF cells. However, recent findings have alluded to heterogeneity within the zF based on variable proliferative capacities, sex differences, and gradients of signaling pathway activation. For example, upon chronic stress, adrenal enlargement results from hyperplasia in the outer portion of the zF and hypertrophy in the innermost region^16^, suggesting multiple subpopulations of GC-producing cells within the zF^17^. New refined sequencing techniques have been applied to the adrenal gland and allowed for the identification of markers of zF subpopulations (e.g. C*yp2f2*^18^ and *Abcb1b*^19^).

As we and others have shown, zF cell diversity arises in part from the intersection of opposing signaling gradients in the adrenal cortex. The homeostatic maintenance of the adrenal cortex is dependent on a centripetal gradient of Wnt/β-catenin activity, regulated in the upper zF by the E3-ubiquitin ligase ZNRF3^20^, and reciprocally inhibited by a centrifugal cAMP/PKA gradient^21^. Disruption of the gradient of either cell signaling pathway results in hormonal disorders^22^ accompanied by alterations of cell differentiation, such as prevention of the zona glomerulosa (zG) to zF zonal transition^23^ or the induction of zona reticularis (zR)-like identity of inner zF cells^24–26^. While such data are consistent with controlled heterogeneity being necessary for adequate zF function, little is known about the rules that govern the cellular diversity within the zF. Additionally, intrinsic sex differences in mammalian adrenal biology are well documented but still poorly understood. For instance, androgens have been shown to markedly influence adrenocortical growth, differentiation, and zonation^24,27–30^. Nevertheless, sex-dependent and independent zF cellular diversity and function remains to be deciphered. The abundant expression of the androgen receptor AR (NR3C4) in the human^31,32^ and rodent adrenals^33–36^, together with numerous mouse models^29,33,34^ and pharmacological studies, indicate that the adrenal gland is a major androgen-responsive organ^37^. Taken together, these studies illustrate that the zF is molecularly heterogeneous and sexually dimorphic.

We describe here for the first time the role of the transcription factor HHEX in adrenal homeostasis and function using male and female mice. HHEX (hematopoietically expressed homeobox), also known as HEX and PRH (Proline-Rich Homeodomain protein), was initially described in the hematopoietic lineage, where it acts as a transcriptional repressor necessary for the maturation and proliferation of definitive hematopoietic progenitors^38–43^. HHEX has since been revealed to function as a transcriptional activator in differentiation processes across various organs^44–50^. Besides its role during development and organogenesis, HHEX expression has also been implicated in adult endocrine tissue homeostasis, including in the pancreas, where it controls the maintenance of somatostatin-secreting delta cell differentiation^51^. Indeed, polymorphisms in the HHEX gene have been associated with type 2 diabetes in Genome-Wide Association Studies (GWAS)^52–59^. While evidence for a functional role in additional endocrine cell types is lacking, meta-analyses have detected a human *HHEX* SNP (rs2497306) associated with serum levels of adrenal-derived dehydroepiandrosterone sulfate (DHEAS)^60,61^, suggesting a role for HHEX in adrenal steroidogenesis.

In this study, we combine single-cell RNA sequencing (scRNA-seq) and *in vivo* knockout of candidate genes to uncover factors that orchestrate cellular diversity in the zF and regulate GC production. We demonstrate that HHEX is uniquely expressed in the zF of rodent and human adrenals. Using the mouse as a model organism, we show that loss of HHEX leads to GC deficiency in males. Importantly, in the male adrenal, we find that HHEX protects the inner zF from androgen-induced lipophagy (lipid depletion) at puberty. Molecularly, HHEX exerts both androgen-dependent and independent functions. We demonstrate that HHEX is a *bona fide* androgen receptor (AR) target gene using CUT&Tag technology^62^ and shapes the AR-driven sexual dimorphism of the adrenal gland in the zF. Finally, in both male and female mice, HHEX maintains the expression of GC exporter *Abcb1b* in the inner zF at baseline and is necessary for the expansion of the *Abcb1b*-positive domain of expression during chronic stress.

## RESULTS

### scRNA-seq reveals heterogeneity within the steroidogenic lineage of the adrenal cortex.

**Figure 1:**
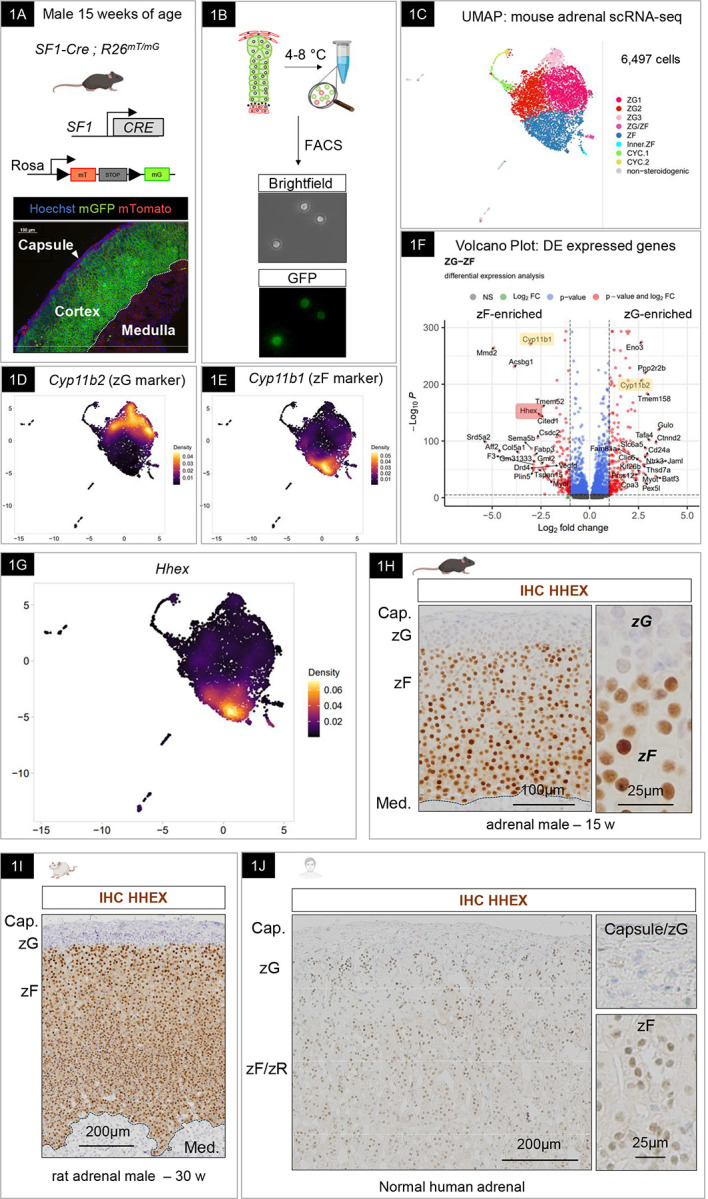
scRNA-seq reveals heterogeneity within the steroidogenic lineage of the adrenal cortex and identified HHEX as a novel and highly conserved marker of GCs-producing cells. (**A**) SF1-Cre mice were bred with Rosa^mT/mG^ mice to label steroidogenic lineage with membrane Green Fluorescent Protein (mG). Non-recombined cells express Tomato (mT). Fluorescent image from a cryosection of an adult male SF1-Cre; mT/mG adrenal gland depicting endogenous mGFP (steroidogenic cells) and mTomato fluorescence. Hoechst was used to stain the nuclei. (**B**) Enzymatic and mechanic single-cell dissociation at low temperature, followed by FACS sorting. The image depicts GFP-positive cells after sorting. (**C**) UMAP representation of scRNA-seq dataset depicting steroidogenic cell populations in the adult male adrenal. zG: zona glomerulosa, zF: zona fasciculata, CYC: cycling. (**D-E**) UMAP representation of *Cyp11b2* (**D**) and *Cyp11b1* (**E**) expression in the scRNA-seq dataset. (**F**) Volcano Plot representing differentially expressed genes between zG and zF. *Cyp11b1* and *Cyp11b2* are highlighted in yellow and *Hhex* is highlighted in red. (**G**) UMAP representation of *Hhex* expression in the scRNA-seq dataset. (**H-I-J**) Expression of HHEX in the mouse (**H**), rat (**I**) and human (**J**) adrenal gland in brown by immunohistochemistry (IHC). Nuclei were stained in blue with hematoxylin.

To achieve single-cell resolution of the adrenocortical transcriptome, we selectively labeled steroidogenic adrenocortical cells using a transgenic Cre-LoxP approach. We combined *Sf1-Cre*^*high* 63^ mice with *Rosa*^*mT/mG* 64^ animals to obtain mice expressing fluorescent reporter proteins at the cell membrane (mT/mG) of cells that express or have expressed the transcription factor SF-1 (Steroidogenic Factor 1, also AdBP4, encoded by *NR5A1*) (Figure 1A). Consistent with previous results using these transgenic mice, only the adrenal cortex was labeled with green fluorescence (mGFP), leaving the capsule and the medulla fluorescent in the red channel (mTomato)^20,65,66^. We prepared a single-cell suspension by combining mechanical and enzymatic dissociation at low temperatures to preserve the viability of steroidogenic cells and their transcriptional state. Fluorescence-activated cell sorting (FACS) was then used to isolate GFP-positive cells representing the steroidogenic lineage (Figure 1B). GFP-positive cells include the progenitor populations as well as differentiated cells (zG and zF cells). Libraries for scRNA-seq were generated using the 10X Genomics platform. Following quality control and filtering out poor-quality cells, a total of 6,497 cells from two replicates were retained for subsequent analyses. A principal component analysis (PCA), considering a gene set of the most variably expressed genes, distinctly separated cells according to their identity. We then annotated 9 clusters according to the expression of known feature genes (Figure 1C and Supplementary Figure 1a). Among the cells, 97% highly expressed GFP, demonstrating the successful enrichment of cells belonging to the steroid lineage. As expected, no medulla cells were found in our dataset. This was shown by the absence of *Th*, *Dbh*, and *Pnmt* expressing cells which are well-known markers of chromaffin cells and are involved in catecholamine synthesis. Across the dataset, we identified the mineralocorticoid-producing zG based on the expression of *Cyp11b2* (Figure 1D) and the glucocorticoid-producing zF based on the expression of *Cyp11b1* (Figure 1E). Among the cells, 35% expressed *Cyp11b2*, 43% expressed *Cyp11b1*, and 13% expressed both. We identified the proliferative population based on the expression of *mKi67* (Supplementary Figure 1b). We confirmed the presence of a recently described unique cell population defined by high expression of *Abcb1b*, *Sbsn*, *Mgst2,* and *Srd5a2* identified by Lopez and colleagues^19^, representing 3–5% of the *Cyp11b1*^+^ population (Supplementary Figure 1c). As expected for adult mouse adrenals, the expression of *Cyp17a1* and *Sult2a1*, markers of the zR were absent from the dataset. To provide user-friendly access to this dataset to the scientific community, we used the 10x Genomics’ LoupeR package to generate a CLOUPE file that can be easily imported into the 10X Genomics Loupe Browser for data visualization and further exploration (See Material and Methods). **To summarize, our scRNA-seq dataset provides an advanced atlas of the adrenal steroid lineage in the male mouse adrenal which can be used to identify new potential regulators of steroid function.**

### HHEX is a novel and highly conserved marker of glucocorticoid-producing cells.

To gain insights into transcripts that underly differences in zonation (zG versus zF) of the cortex and provide a thorough understanding of the zF transcriptome, we analyzed the most enriched genes in zG and zF cell populations (Figure 1F–G). Our dataset revealed specific enrichment of new markers in the zG such as *Ppp2r2b* (a Wnt pathway antagonist), and *Pcdh19* (a proto-cadherin involved in cell-to-cell adhesion) (Supplementary Figure 1d). Besides the *bona fide* marker *Cyp11b1*, we found the expression of genes encoding proteins, such as *Mmd2* (a membrane progestin receptor), *Acsbg1* (an acyl-CoA synthetase), and lipid membrane transporters such as *Abca1* (Supplementary Figure 1e) were enriched in the zF.

To uncover new potential modulators of zF function, we focused our analysis on factors that could directly control gene expression, such as DNA-binding proteins. This analysis identified *Hhex* as the top enriched transcription factor in the zF (Figure 1G). HHEX (also known as PRH) belongs to the homeobox family and is involved in differentiation and proliferation processes^67^. To validate these results, we performed immunohistochemistry and confirmed that HHEX expression is absent from DAB2-positive cells, marking the zG (Supplementary Figure 1f), and is limited to the zF in mouse (Figure 1H) and adult male rat adrenals (Figure 1J). Using publicly available datasets, we found that human adrenal tissue is among the organs that express the highest levels of HHEX (Supplementary Figure 1g). Using immunohistochemistry, we confirmed that HHEX is expressed in the zF of the human adrenal cortex (Figure 1J). **In conclusion, we were able to define HHEX as a new marker of the zF that is conserved across species, which supports a potential role in zF function.**

### Genetic inactivation of *Hhex* in the mouse adrenal cortex impairs glucocorticoid levels in males.

**Figure 2:**
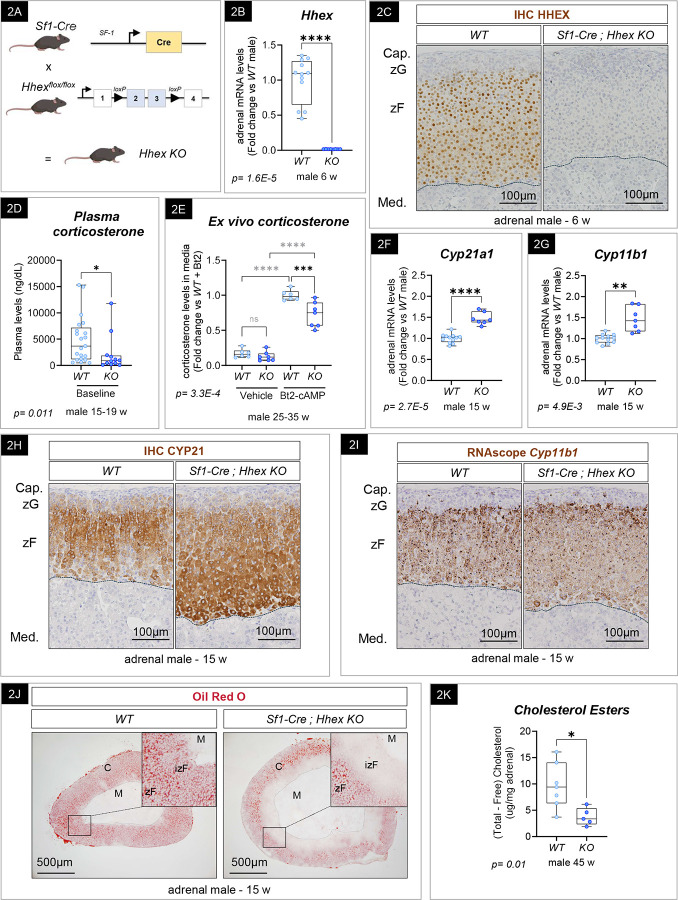
*Hhex KO* male mice develop GC insufficiency. (**A**) Schematic representation of the transgenic mouse models used to inactivate HHEX in adrenocortical cells using SF1-Cre-mediated recombination of exon 2 and 3 of *Hhex* gene. (**B**) Quantification of *Hhex* transcripts by RT-qPCR in 6-week-old *WT* (n=12) and *SF1-Cre Hhex KO* (n=8) male adrenals. Graph represents box plots with individual biological replicates. *p*-value (*p*) was calculated using a Mann-Whitney test. (**C**) Expression of HHEX in 6-week-old *WT* and *SF1-Cre Hhex KO* male adrenals in brown by immunohistochemistry (IHC). Nuclei were stained in blue with hematoxylin. Dotted lines represent the corticomedullary junction. *WT*: Wild Type, *KO*: Knockout, Cap.: Capsule, zG: zona glomerulosa, zF: zona fasciculata, Med.: medulla. (**D**) Corticosterone plasma levels of 15–19 week-old *WT* (n=21) and *SF1-Cre; Hhex KO* males (n=12) Graph represents box plots with individual biological replicates. *p*-value (*p*) was calculated using a Mann-Whitney test. (**E**) *Ex vivo* corticosterone production, released in culture media by adrenal explants after 2.5 hours incubation with 2.5mM of Bt2. *WT* (n=6) and *SF1-Cre; Hhex KO* males (n=7). Graph represents box plot with individual biological replicates. *p*-value (*p*) was calculated using a 2-way ANOVA followed by a Šídák’s multiple comparisons test. (**F**) Quantification of *Cyp21a1* transcripts by RT-qPCR in 15-week-old WT (n=12) and *SF1-Cre Hhex KO* (n=7) male adrenals. Graph represents box plots with individual biological replicates. *p*-value (*p*) was calculated using a two-tailed unpaired t-test with Welch’s correction. (**G**) Quantification of *Cyp11b1* transcripts by RT-qPCR in 15-week-old *WT* (n=12) and *SF1-Cre Hhex KO* (n=7) male adrenals. Graph represents box plots with individual biological replicates. *p*-value (*p*) was calculated using a two-tailed unpaired t-test with Welch’s correction. (**H**) CYP21A1 immunohistochemistry (IHC) in adrenals of *WT* and *SF1-Cre; Hhex KO* adrenals 15-week-old male mice. Nuclei were stained in blue with hematoxylin. Dotted lines represent the corticomedullary junction. *WT*: Wild Type, *KO*: Knockout, Cap.: Capsule, zG: zona glomerulosa, zF: zona fasciculata, Med.: medulla. (**I**) *Cyp11b1* RNAscope in adrenals of *WT* and *SF1-Cre; Hhex KO* adrenals 15-week-old male mice. Nuclei were stained in blue with hematoxylin. Dotted lines represent the corticomedullary junction. *WT*: Wild Type, *KO*: Knockout, Cap.: Capsule, zG: zona glomerulosa, zF: zona fasciculata, Med.: medulla. (**J**) Oil Red O staining of 15-week-old *WT* and *SF1-Cre; Hhex KO* male adrenals. *WT*: Wild Type, *KO*: Knockout. Dotted lines represent corticomedullary junction. C: cortex, M.: medulla, zF, zona fasciculata, izF: inner zona fasciculata. (**K**) Cholesterol esters quantification in adrenals of 45-week-old *WT* and *SF1-Cre; Hhex KO* male. Graph represents box plots with individual biological replicates. *p*-value (*p*) was calculated using a two-tailed unpaired t-test with Welch’s correction.

*In vivo* studies have shown that *Hhex* is expressed in the endocrine lineage of the pancreas and is involved in hormone production and proliferation^51^. However, the role of HHEX in regulating adrenal homeostasis and function remains unknown. To gain insights into HHEX function in the adrenal cortex, *in vivo*, we generated a series of *Hhex* knockout models. First, *Hhex*^*flox/flox*^ mice were crossed with mice expressing *Cre* driven by the *Sf1* promoter^63^ to allow for *Hhex* genetic ablation in the entire adrenal cortex from embryonic day E9.5 (*Sf1-Cre*^*high*^*; Hhex*^*flox/flox*^) (Figure 2A). To analyze the *Hhex KO* (Knockout) phenotype, we collected tissue and plasma samples from 6-, 15-, and 50/55-week-old mice for adrenal histology, transcriptomic analysis, and glucocorticoid measurements. Genetic ablation of *Hhex* was validated in 6-week-old mice by RT-qPCR from mRNAs extracted from whole adrenals and immunohistochemistry. As anticipated, we observed a 99% decrease in *Hhex* expression by RT-qPCR in 6-week-old males (Figure 2B) and 89% in females (Figure 6B). Residual *Hhex* mRNA expression is likely due to *Hhex* expression in non-steroidogenic cells devoid of Cre expression. Indeed, HHEX expression has been previously found in other adult lineages, such as the hematopoietic compartment^38,43^, the endothelial cell population^68,69^, and in resident immune cells^70,71^. As expected, no HHEX staining was observed in *Sf1-Cre*^*high*^ *Hhex KO* animals by immunohistochemistry, contrary to the potent nuclear signal observed in Wild Type (*WT*) and heterozygotes animals in the zF (Figure 2C). We also assessed HHEX expression in other SF-1-expressing organs that could have a direct effect on adrenal function, such as the pituitary gland or the gonads. In the pituitary, the corticotropic cells responsible for ACTH release do not express SF-1^63^ and are consequently not targeted by Cre expression^20,66^. Using pituitary single-cell data^72,73^, we observed *Hhex* was absent from the *Lhb*^*+*^ cell population, the *Sf1*-expressing gonadotroph population^74^, and solely expressed in *Pecam*^*+*^ and *C1qa*^*+*^ cells (data not shown). Since SF-1 is also expressed in somatic cells of the gonads^63^, we assessed HHEX expression in both testis and ovaries. No staining was discernible (Supplementary Figure 2a), ruling out a major effect of gonadal *Hhex* deletion on the adrenal. Finally, we used a zF-specific tamoxifen-inducible Cre model, using the recently developed *Cyp11b1-Cre*^*ERT2*^ mouse, to target *Hhex* deletion and validated key phenotypic findings (Supplementary Figure 2b and Supplementary Figure 6b).

To determine if HHEX contributes to zF function, we first measured plasma GC levels by mass spectrometry in *WT* and *Hhex KO* mice. We observed a significant 55% decrease in baseline corticosterone in adult males (Figure 2D). We then cultivated adrenal explants in the presence of dibutyryl-cAMP (Bt2-cAMP) for 2.5 hours to stimulate GC production and release, and observed a 28% decrease in corticosterone levels in media of *Hhex KO* compared to *WT* explants (Figure 2E). These results show that HHEX is required to maintain normal GC production and/or secretion. Despite the fact that the adrenal gland’s main function is to produce steroids, adrenal cells do not store GC. Instead, they rapidly metabolize cholesterol and engage the steroidogenic pathway to produce steroids on demand^75–80^. To further interrogate steroidogenic potential in the *Hhex KO* adrenals, we first assessed the expression of steroidogenic enzymes such as *Cyp21a1* and *Cyp11b1* by RT-qPCR (Figure 2F–2G) and immunohistochemistry for *in situ* localization (Figure 2H–2I). Given the observed GC deficiency in *Hhex KO*, we expected that steroidogenic enzyme expression would be decreased, but surprisingly, we observed an increase in expression and no change in tissue distribution. The entire zF was positive for both enzymes. This result made us consider the possibility that GC insufficiency was unlikely to be due to a defect in steroidogenesis, but was instead a consequence of a proximal upstream defect leading to compensatory upregulation of steroidogenic enzyme gene expression.

To produce steroid hormones on demand, adrenocortical cells metabolize cholesterol (the precursor to all steroids^81,82^). Storage of cholesterol, in the form of lipid droplets (LD), allows for a reservoir of this precursor to be available when needed. Thus, we evaluated cholesterol content in *Hhex KO* by first detecting neutral lipids such as cholesteryl esters (CE) and triacylglycerol (TG) stored in lipid droplets using Oil Red O staining. By comparing *WT* and *Hhex KO* frozen stained adrenal sections, we observed a striking reduction of neutral lipids stored in LD in the inner part of the cortex in 15-week-old *Sf1-Cre; Hhex KO* male mice (Figure 2J). This observation was confirmed by the quantification of intra-adrenal cholesterol esters by colorimetric assay (Figure 2K), which demonstrated a 61% decrease of CE in the adrenal of *Hhex KO* compared to their littermate *WT* (30–55-week-old) controls. **Taken together, these results suggest that HHEX is a critical transcription factor that serves to maintain glucocorticoid levels *in vivo* in males.**

### Lipid depletion observed in *Hhex KO* males results in part from activation of lipophagy in the inner zF.

To better understand the mechanisms by which HHEX protects the sterol content of the zF, we interrogated cholesterol ester uptake, lipid droplet catabolism, and *de novo* synthesis. In rodents, adrenocortical cells obtain extracellular cholesterol mainly through the import of High Density Lipoprotein particles from the circulation^83^. Analysis by immunohistochemistry demonstrated that the inner cortex of *Hhex KO* strongly maintained the expression of SR-B1, the receptor for CE-laden HDL particles (Supplementary Figure 3a), suggesting cholesterol uptake is not perturbed in the inner zF cells of *Hhex KO* adrenals. The expression of *Hmgcr*, the rate-controlling enzyme of the mevalonate pathway was significantly upregulated (Supplementary Figure 3b), suggesting *de novo* synthesis was not impaired. Taken together, these results indicated that defects in cholesterol uptake and/or synthesis were unlikely the driving mechanism of lipid depletion in *Hhex KO*.

**Figure 3:**
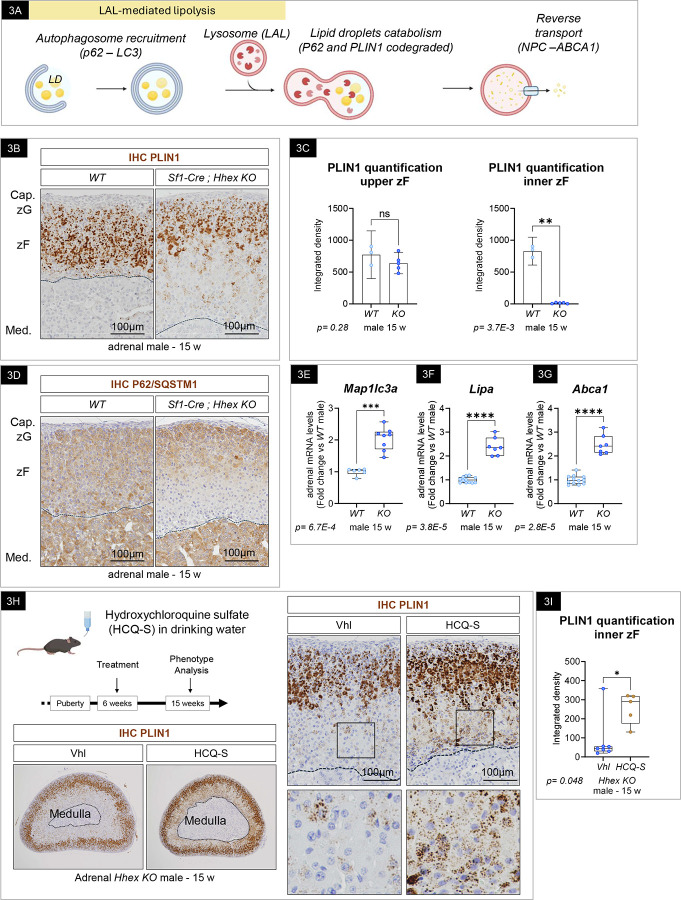
*Hhex KO* adrenals display signs of activated lipophagy. (**A**) Schematic of lipophagy also known as LAL-mediated lipolysis. (**B**) PLIN1 by immunohistochemistry (IHC) in adrenals of 15-week-old *WT* and *SF1-Cre; Hhex KO* adrenals. Nuclei were stained in blue with hematoxylin. Dotted lines represent the corticomedullary junction. *WT*: Wild Type, *KO*: Knockout, Cap.: Capsule, zG: zona glomerulosa, zF: zona fasciculata, Med.: medulla. (**C**) Quantification of PLIN1 immunohistochemistry in the upper zF and inner zF using imageJ. Graph represents mean with 95% Confidence Interval and individual biological replicates. *p*-value (*p*) was calculated using a two-tailed unpaired t-test with Welch’s correction. ns: non-significant. (**D**) P62/SQSTM1 immunohistochemistry (IHC) in adrenals of 15-week-old *WT* and *SF1-Cre; Hhex KO* adrenals. Nuclei were stained in blue with hematoxylin. Dotted lines represent the corticomedullary junction. *WT*: Wild Type, *KO*: Knockout, Cap.: Capsule, zG: zona glomerulosa, zF: zona fasciculata, Med.: medulla. (**E-G**) Quantification of *Map1lc3a* (**E**) (n=6 and 8), *Lipa* (**F**) (n=12 and 7), and *Abca1* (**G**) (n=12 and 7) transcripts by RT-qPCR in 15-week-old *WT* and *SF1-Cre; Hhex KO* male adrenals. (**H**) PLIN1 immunohistochemistry (IHC) in adrenals of *SF1-Cre; Hhex KO*, Vehicle and hydroxychloroquine sulfate-treated 15-week-old male mice. Nuclei were stained in blue with hematoxylin. Dotted lines represent the corticomedullary junction. *WT*: Wild Type, *KO*: Knockout, Cap.: Capsule, zG: zona glomerulosa, zF: zona fasciculata, Med.: medulla. (**I**) Quantification of PLIN1 immunohistochemistry in the inner zF using imageJ. Vehicle n=7, HCQ-s n=5. Graph represents box plots with individual biological replicates. *p*-value (*p*) was calculated using a Mann-Whitney test.

To identify potential alternative mechanisms of lipid depletion, we performed a detailed analysis of the bulk RNAseq from male *Hhex KO* versus *WT* adrenals. This revealed activation of acidic lipolysis by ‘lipophagy’, which is the selective engulfment of lipid droplets by the autophagic machinery (Figure 3A). In brief, this process has been shown to involve the sequestration of lipid droplets by LC3-II positive membranes, where the macroautophagy cargo receptor p62/SQSTM1 bridges LD with the autophagic membrane, the phagophore^84^. The recruitment of p62/SQSTM1 to the LDs is in part mediated by the perilipin PLIN1, an LD-associated protein^85^. Upon fusion with the lysosomes, where the lipase lysosomal Acid Lipase (LAL) resides, lipid droplets are degraded and cholesterol content recycled. Of note, it has been suggested that Perilipins and p62/SQSTM1 are co-degraded with lipid droplets in lysosomes; whereby their expression is inversely correlated with autophagic activity^84,86,87^. We therefore evaluated the integrity of lipophagy in *WT* and *Hhex KO* adrenals. In *WT* adrenals, PLIN1 (Figure 3B, left) and p62/SQSTM1 staining (Figure 3D, left) was robust throughout the cortex, while absent in the capsular cells. In *Hhex KO,* both PLIN1 and p62/SQSTM1 were markedly decreased in the inner zone of the cortex (Figure 3B and 3D, right), mirroring the lipid-depleted area. Specifically, PLIN1 staining was quantified using image processing analysis (Supplementary 3d) demonstrating that the inner zF was significantly affected compared to the upper zF, which displayed similar staining intensities in *WT* and *Hhex KO* (Figure 3C). In parallel, we confirmed by RT-qPCR the expression of key effectors of lipophagy and observed a significant increase in *Map1lc3a* (encoding the autophagic protein LC3), *Lipa* (encoding the acidic lipase LAL) (Figure 3E–3F) and *Npc2* (encoding a lysosomal protein responsible for cholesterol removal from lysosomes) (Supplementary Figure 3c) coincident with a marked increase in *Abca1* (encoding a transporter known to limit the intracellular free concentration by promoting cholesterol efflux referred to as reverse cholesterol transport) (Figure 3G). To test the hypothesis that lipophagy activation contributes to the absence of lipid droplets, we temporally and pharmacologically inhibited autophagy *in vivo* by treating *WT* and *Hhex KO* mice with hydroxychloroquine sulfate (HCQ-S), which prevents the fusion of the autophagosomes with lysosomes^88^. In *WT,* HCQ-S treatment did not affect the domain of expression of PLIN1 compared to vehicle-treated mice (Supplementary Figure 3e). In contrast, we observed a partial lipid rescue specifically in the inner part of the cortex of *Hhex KO* male mice (Figure 3H), which was quantified using image processing analysis (Figure 3I). **Together, these results indicate a crucial role for HHEX in protecting inner zF cells from lipophagy-mediated lipid depletion.**

### HHEX-dependent lipid depletion and HHEX expression are androgen-mediated.

**Figure 4:**
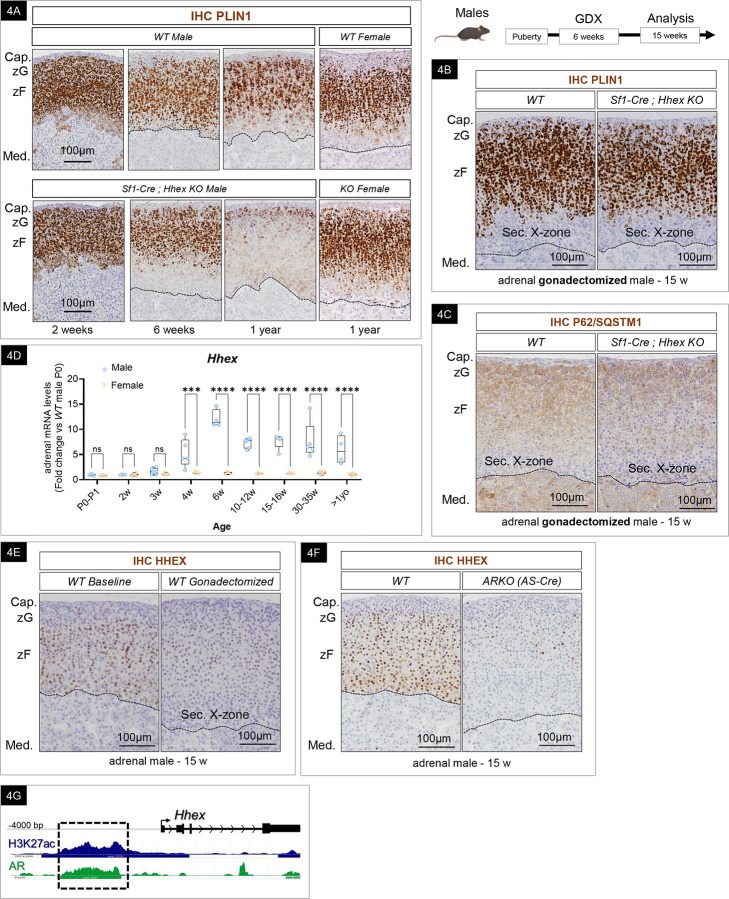
Lipid depletion in *Hhex KO* male adrenals is androgens driven. (**A**) PLIN1 immunohistochemistry (IHC) in adrenals of *WT* and *SF1-Cre; Hhex KO* adrenals at 2, 6, 15 weeks old and over 1-year-old male and female mice. Nuclei were stained in blue with hematoxylin. Dotted lines represent the corticomedullary junction. *WT*: Wild Type, *KO*: Knockout, Cap.: Capsule, zG: zona glomerulosa, zF: zona fasciculata, Med.: medulla. (**B**) PLIN1 immunohistochemistry (IHC) in adrenals of 15 week-old gonadectomized *WT* and *SF1-Cre; Hhex KO* male mice. Nuclei were stained in blue with hematoxylin. Dotted lines represent the corticomedullary junction. GDX: gonadectomy, *WT*: Wild Type, *KO*: Knockout, Cap.: Capsule, zG: zona glomerulosa, zF: zona fasciculata, Med.: medulla. Sec. X-zone: gonadectomy-induced secondary X-zone. (**C**) P62/SQSTM1 (lipophagy marker) immunohistochemistry (IHC) in adrenals of 15 week-old gonadectomized *WT* and *SF1-Cre; Hhex KO* male mice. Nuclei were stained in blue with hematoxylin. Dotted lines represent the corticomedullary junction. GDX: gonadectomy, *WT*: Wild Type, *KO*: Knockout, Cap.: Capsule, zG: zona glomerulosa, zF: zona fasciculata, Med.: medulla. Sec. X-zone: gonadectomy-induced secondary X-zone. (**D**) Timecourse of *Hhex* expression by RT-qPCR in male and female adrenal gland from birth to over 1 year old. Graph represents box plots with individual biological replicates. *p*-value (*p*) was calculated using a 2-way ANOVA followed by a Šídák’s multiple comparisons test. The number of biological replicates for each condition and a complete list of *p*-values are provided in the supplementary table related to Statistics (**E**) HHEX expression by immunohistochemistry (IHC) in the adrenals of 15-week-old *WT* intact and gonadectomized male adrenals. Nuclei were stained in blue with hematoxylin. Dotted lines represent the corticomedullary junction. GDX: gonadectomy, *WT* = Wild Type, Cap.: Capsule, zG: zona glomerulosa, zF: zona fasciculata, Med.: medulla. Sec. X-zone= gonadectomy-induced secondary X-zone. (**F**) HHEX expression by immunohistochemistry (IHC) in AS-Cre; ARKO. Nuclei were stained in blue with hematoxylin. Dotted lines represent the corticomedullary junction. *WT*: Wild Type, *KO*: Knockout, Cap.: Capsule, zG: zona glomerulosa, zF: zona fasciculata, Med.: medulla. Sec. X-zone: gonadectomy-induced secondary X-zone. (**G**) Histogram depicting the accumulation of androgen receptor (AR) CUT&Tag products obtained from adult male adrenal glands matching the *Hhex* promoter. H3K27ac is used to identify the open chromatin regions of the genome.

To characterize the temporal dynamic of the LD observed in *Hhex KO* adrenals, we assessed PLIN1 expression by immunohistochemistry at multiple time points (in 2-week-old, 6-week-old, and > 1-year-old mice) (Figure 4A). The results revealed that the genetic loss of HHEX prompted a decrease in lipid droplets in *Hhex KO* male adrenals that became visible at 6 weeks of age. This lipid depletion was noted primarily in the inner zF and expanded over time. With age, the entire zF was substantially lipid depleted. However, in 2-week-old prepubertal mice, *WT* and *Hhex KO* male adrenals were indiscernible, coincident with intense staining for PLIN1 throughout the cortex in both genotypes. These results indicate that lipid depletion in the *Hhex KO* adrenal begins near puberty and suggests that HHEX is required to actively maintain LD stores from this stage onward. Contrary to what was observed in aged *Hhex KO* male adrenals, we did not observe a drastic LD depletion in females over 1 year of age (Figure 4A, right). Consistently, when stimulated with Bt2-cAMP, *WT* and *Hhex KO* female adrenal explants released an equivalent amount of corticosterone into the media (Supplementary Figure 4a). Of note, we observed that the X-zone was devoid of lipid droplets, which complicated the analysis of the lipid depletion phenotype in females and gonadectomized male mice. To circumvent this issue, we also performed immunohistochemistry for PLIN1 on aged *Hhex KO* females who carried one or more pregnancies resulting in the well-characterized complete regression of the X-zone. We did not observe any loss of lipid droplets in *Hhex KO* primiparous females (Supplementary Figure 4b).

To explain the decrease of lipid stores observed only in *Hhex KO* males after puberty, we hypothesized an interplay between lipid droplet catabolism and androgen signaling in the adrenal zF. We postulated that the androgenic milieu is the driving force of lipophagy and that HHEX acts as a protective mechanism to maintain LD integrity. To test this hypothesis, we gonadectomized *WT* and *Hhex KO* males at 6 weeks of age, when lipid depletion is first observed, and analyzed the adrenal glands at 15 weeks of age, when LD depletion is observed in the majority of the zF cells in non-gonadectomized *Hhex KO* males. In accordance with our hypothesis, we observed a rescue of lipid droplets in the zF of gonadectomized *Hhex KO*, becoming visually indistinguishable from their gonadectomized *WT* littermates (Figure 4B). As previously mentioned, the gonadectomy-induced secondary X-zone was negative for PLIN1 expression in both *WT* and *Hhex KO*. The lipid rescue observed upon gonadectomy was also seen when *Hhex* was deleted using the *AS-Cre* mouse line, which targets the definitive cortex^27^, thus ruling out the role of the X-zone (not derived from the outer zG) as a contributing source of the observed phenotype (Supplementary Figure 4c). Consistently, p62/SQSTM1 staining was restored in the inner cortex of previously gonadectomized *Hhex KO* (Figure 4C). Together, these data support the hypothesis that lipid depletion in *Hhex KO* results from an increase in autophagic flux that is specifically the result of androgen-driven lipophagy.

The adrenal glands rely on a high content of cholesterol ester to support steroidogenesis, which suggests that the catabolic action of the androgens on LD content needs to be tightly controlled. Therefore, we questioned the possible signaling pathways controlling HHEX expression. We hypothesized that to protect the LDs at puberty, HHEX expression itself might be regulated by androgens. To address this question, we first looked at the expression of HHEX at different time points from birth through aging past 1-year, in both males and females. At the mRNA level, we observed a dramatic increase at puberty, specifically in males (between 3 and 4 of age), resulting in an 9-fold difference between sexes at 6 weeks of age (Figure 4D). We then proceeded to assess the expression of HHEX in the absence of circulating androgens. We analyzed adrenals from adult gonadectomized male mice and observed a striking decrease in HHEX staining compared to their non-gonadectomized littermates (Figure 4E). Next, we confirmed the direct contribution of androgens to the upregulation of HHEX expression *ex vivo* while ruling out the participation of gonadotrophins released by the central system. We incubated female adrenal explants with 5a-Androstan-17b-ol-3-one (DHT) (an androgen, non-aromatizable into estrogens^89,90^) to activate androgen signaling. Adrenal explants incubated with DHT for 48 hours led to a significant 2.2-fold increase in *Hhex* expression compared to the control treated explants (Supplementary Figure 4d). This result confirms the direct role of androgens in promoting HHEX expression in adrenocortical cells.

We then tested whether *Hhex* expression is directly regulated through AR signaling. AR is known to be expressed and to function in the adrenal cortex^33,34^, so we proceeded to look at HHEX expression following adrenal-specific AR deletion in the adrenal cortex (*ARKO*) by breeding *AR flox*^91^ mice with *AS-Cre*^27^ mice. Similar to our findings upon gonadectomy in *WT* mice, HHEX expression was dramatically reduced in the adrenals of adult adrenal-specific *ARKO* mice (Figure 4F). Finally, we employed CUT&Tag, a technique based on the same principles as Chromatin Immunoprecipitation (ChIP) that offers the advantage of being more sensitive and requiring less material^62^, which makes it suitable to study binding profiles of transcription factors *in vivo* in the mouse adrenal gland. Using an antibody targeting AR to map genomic AR binding sites in the mouse adrenal, we identified 106,726 peaks genome-wide. This finding underscores the significant role that AR plays in transcriptional regulation in the male mouse adrenal. Next, we conducted *de novo* motif analysis using homer and identified enrichment for the motif – AGTACACATAMTGT-, which carries similarities to known steroid receptor binding motifs, including AR (MA0007.3 in JASPAR). In exploring a possible connection between AR and *Hhex* expression, we identified in *WT* male adrenals an AR peak in the *Hhex* promoter that overlaps with a H_3_K_27ac_ peak (Figure 4G). H_3_K_27ac_ is a histone modification associated with active chromatin, typically found at active enhancers and promoters, indicating potential transcriptional activation. The sexually dimorphic expression of HHEX in the adrenal cortex led us to examine other HHEX-expressing, sexually dimorphic tissues, such as the liver and the pancreas^92,93^. Contrary to the adrenal gland, HHEX was strongly expressed in both male and female hepatocytes (liver), somatostatin-secreting delta cells (pancreas), and hematopoietic cells from the bone marrow, where it was first identified (Supplementary Figure 4e). **Altogether, these results demonstrate that HHEX is a *bona fide* AR-target gene in the adrenal zF, which coordinates the precise timing of protecting the integrity of lipid droplets at puberty.**

### Adrenal sexual dimorphism is partially mediated by HHEX.

**Figure 5:**
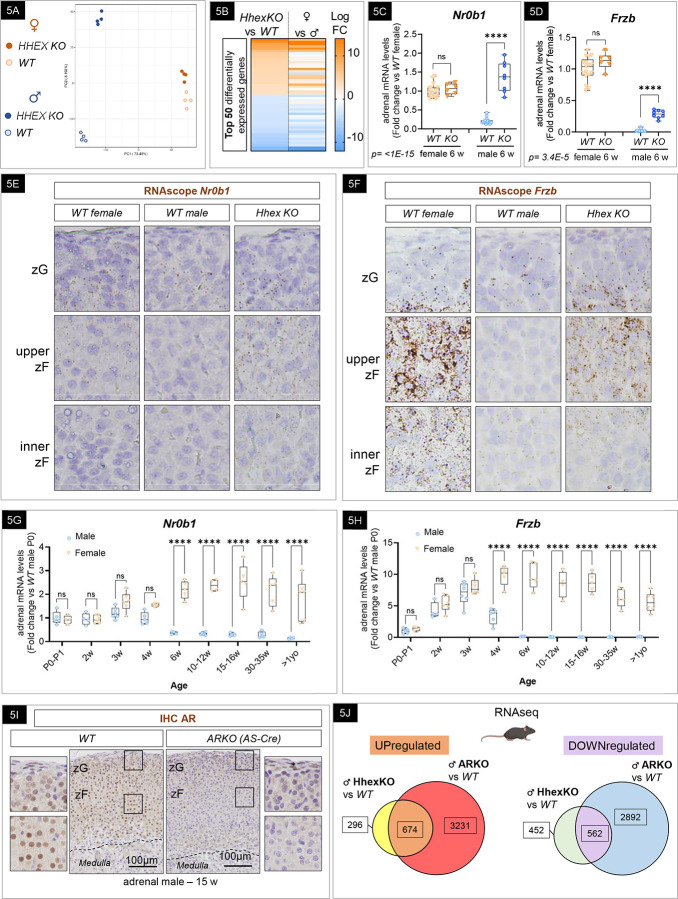
HHEX shapes the sexual dimorphism of the transcriptome of the zF. (**A**) Principal Component Analysis (PCA) plots from bulk RNAseq analysis of 6-week-old *WT* and *SF1-Cre; Hhex KO* male and female adrenal glands (n=4 for each group). (**B**) Heatmaps of the top 50 differentially expressed genes between male *SF1-Cre; Hhex KO* and *WT* compared to dimorphic genes. (**C-D**) *Nr0b1* (**C**) and *Frzb* (**D**) expression by RT-qPCR in the adrenal gland of 6-week-old female (▽) and male (_◯_), *WT* (n= 10 & 12) and *SF1-Cre; Hhex KO* (n= 6 & 8). Graph represents box plots with individual biological replicates. *p*-value (*p*) was calculated using a 2-way ANOVA followed by a Šídák’s multiple comparisons test. (**E-F**) Expression of *Nr0b1* (**E**) and *Frzb* (**E**) by RNAscope in adrenal glands of 6-week-old *WT* female and *WT* and *SF1-Cre; Hhex KO* male. (**G-H**) Timecourse of *Nr0b1* (**G**) and *Frzb* (**H**) expression by RT-qPCR in male (_◯_) and female (▽) adrenal gland from birth to over 1 year old. Graph represents box plots with individual biological replicates. *p*-value (*p*) was calculated using a 2-way ANOVA followed by a Šídák’s multiple comparisons test. The number of biological replicates for each condition and a complete list of *p*-values are provided in the supplementary table related to Statistics (**I**) AR expression by immunohistochemistry (IHC) in 15-week-old *WT* and *ARKO*. Insets are focusing on capsule-zG cells (top) and zF cells (bottom). Nuclei are counterstained with hematoxylin. Dotted line represents the corticomedullary junction. Asterisk is pointing out AR-positive capsular cells in ARKO. zG: zona glomerulosa, zF = zona fasciculata. (**J**) Venn diagram of differentially expressed genes in *SF1-Cre; Hhex KO* and *AS-Cre; ARKO* compared to their respective *WT*.

Recent studies have highlighted the influence of androgens, rather than sex chromosomes, on differentiation, proliferation, and disease development in the adrenal cortex^24,30,94^. Given the sex differences observed in HHEX expression and the direct transcriptional dependency of HHEX expression on AR, we postulated that HHEX is a mediator of AR actions in the zF and contributes to sex-biased transcriptional programs in the adrenal cortex. To decipher the transcriptional programs driven by HHEX in the adrenal cortex, we performed bulk RNAseq on adrenals from *WT* and *Hhex KO* mice at 6 weeks of age in both males and females. For a global analysis of gene expression, we performed PCA, a common unsupervised dimensionality-reduction method to visualize and assess the clustering of the data^95^. PCA revealed that over 80% of the variance between the studied mice was explained by the first and second principal components (PCs) that separated individual mice according to sex on the first component (X-axis) and according to genotype on the second component (Y-axis) (Figure 5A). The clustering suggested that *Hhex* deletion in the steroidogenic lineage drives significant changes at the transcriptome level in the adrenal as soon as 6 weeks of age. To further explore the basis of this change, we examined the differentially expressed genes (DEGs). Using a cut-off of 0.05 for the adjusted *p*-value, the analysis revealed that 3,607 genes were differentially expressed between genotypes in males, with only 23 DEGs in females. By analyzing the top 50 DEGs in 6-week-old *Hhex KO* males versus their *WT* littermates, we observed an enrichment in well-known sexually dimorphic genes^18,24,96,97^ (Figure 5B), suggesting a feminization of the male transcriptome when *Hhex* is deleted. We confirmed these observations by assessing the expression of ‘female-biased’ *Nr0b1* (known as Dax1) (Figure 5C), *Frzb* (Figure 5D), and *Cyp2f2* (Supplementary Figure 5a) in *WT* and *Hhex KO*, male and female, adrenals by RT-qPCR. The sexual dimorphism was completely or at least partially abolished for all the genes tested in *Hhex KO* male adrenals. *In situ*, *Nr0b1*-positive cells assessed by RNAscope were observed in the zF of *Hhex KO* male adrenals (similar to *WT* female), while restricted to the zG in *WT* male adrenals,as previously described^97^ (Figure 5E). The expression of *Frzb* was mostly found in cells of the zF in *Hhex KO* (similar to the females) while absent from the *WT* zF (Figure 5F), and CYP2F2-positive cells, visualized by immunohistochemistry, were limited to the inner zF (Supplementary Figure 5b). By quantifying the expression of *Frzb* and *Nr0b1* by RT-qPCR during postnatal life, we observed a striking decrease in males at puberty, which corresponds to the time when HHEX expression increases, supporting the role of HHEX in repressing female programs and promoting male programs (Figure 5G and 5H). Since testosterone levels (assessed by LC-MSMS) were unchanged in *Hhex KO* compared to *WT* plasma (Supplementary Figure 5c), and HHEX expression was absent in the testis and ovary (Supplementary Figure 2b), we ruled out a contribution of the gonads to the reversal of the sexual dimorphism observed in the adrenal cortex of *HHEX KO* mice. Therefore, we performed immunohistochemistry, and staining revealed high expression of AR in the zF (and to a lesser extent in the zG) (Figure 5I), which was confirmed by scRNA-seq (Supplementary Figure 5d). This suggested that the feminization of *Hhex KO* male adrenals could be a result of the loss of androgen signaling directly in the zF. However, AR expression assessed by RT-qPCR was not abolished in males *Hhex KO* compared to their littermate controls (Supplementary Figure 5e), suggesting that HHEX is unlikely upstream of the androgen signaling. To provide a comprehensive analysis, we compared the transcriptome of 6-week-old *Hhex KO* male adrenals and 25-week-old *ARKO* male adrenals and found a significant overlap. Nearly 70% of genes upregulated in *Hhex KO* male adrenals belong to the list of upregulated genes in ARKO male adrenals, and 55% belonged to the list of downregulated genes (Figure 5J and Supplementary Figure 5f). **Taken together, these results suggest that HHEX is a downstream mediator of AR action and that the female transcriptome in the zF is a common program in both sexes before puberty. During the rise of systemic androgen levels concomitant with testis maturation, HHEX expression emerges throughout the zF, which establishes the mature male adrenal transcriptome and consequently institutes an AR:HHEX-dependent sexual dimorphism in the adrenal cortex**.

### HHEX is crucial for maintaining the identity of the innermost adrenocortical cell subpopulation and promoting *Abcb1b* expression in both sexes

**Figure 6:**
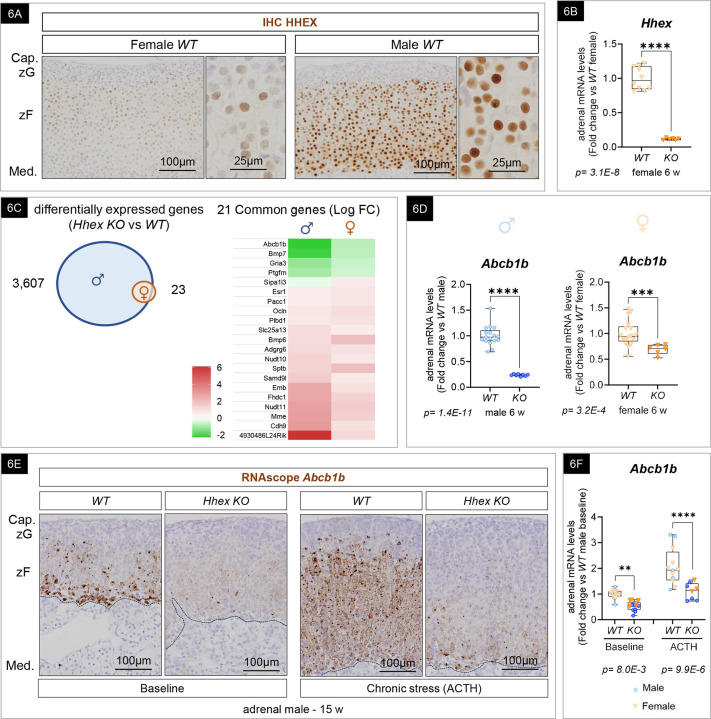
HHEX is required for *Abcb1b* expression in the inner zF of both male and female adrenals. (**A**) HHEX expression in adult female and male adrenal gland by immunohistochemistry (IHC). Nuclei are counterstained with hematoxylin. *WT*: Wild Type, Cap.: capsule, zG: zona glomerulosa, zF: zona fasciculata. (**B**) Quantification of *Hhex* transcripts by RT-qPCR in 6-week-old *WT* (n=10) and *SF1-Cre; Hhex KO* (n=6) female. Graphs represent individual biological replicates and the means with 95% confidence interval. *p*-value (*p*) is calculated using a two-tailed unpaired t-test with Welch’s correction. (**C**) Venn diagram representing the differentially expressed genes in *SF1-Cre; Hhex KO* compared to their respective *WT* (males in blue and females in orange) and heatmap of commonly differentially expressed genes in males and female *SF1-Cre; Hhex KO*. FC: Fold change. (**D**) Quantification of *Abcb1b* transcripts by RT-qPCR in 6-week-old *WT* (n=17 and 17) and *SF1-Cre Hhex KO* (n=8 and 6) male (_◯_) and female (▽) adrenals. Graph represents box plots with individual biological replicates. *p*-value (*p*) is calculated using a two-tailed unpaired t-test with Welch’s correction. (**E**) Schematic of ACTH-induced chronic stress for 10 days in adult mice. *Abcb1* expression in 15-week-old *WT* and *SF1-Cre; Hhex KO* male adrenals by RNAscope, at baseline and after chronic stress. Nuclei are stained in blue with hematoxylin. Dotted lines represent the corticomedullary junction. *WT*: Wild Type, *KO*: Knockout, Cap.: capsule, zG: zona glomerulosa, zF: zona fasciculata, Med: medulla (**G**) Quantification of *Abcb1b* transcripts by RT-qPCR in 15-week-old *WT* (n= 14 and 12) and *SF1-Cre Hhex KO* (n=14 and 8) male (_◯_) and female (▽) adrenals. Graph represents box plots with individual biological replicates. *p*-value (*p*) is calculated using a 2-way ANOVA followed by a Šídák’s multiple comparisons test.

The lower expression of *Hhex* in females compared to males after puberty and the lack of evident LD depletion in female *Hhex KO* mice led us to question whether *HHEX* contributes to transcriptional programs in the female mouse adrenal cortex. Analysis of HHEX expression by immunohistochemistry in *WT* male and female adrenals revealed a striking lower staining intensity in zF cells of female adrenals (Figure 6A). Nonetheless, *Hhex* transcript levels were higher in *WT* female adrenals than their *Hhex KO* littermates, demonstrating the existence of a baseline expression in female adrenal cells (Figure 6B). Additionally, bulk RNAseq analysis of 6-week-old female adrenals revealed 23 significant DEGs between *Hhex KO* and *WT* mice. Accordingly, the PCA plot demonstrated the separation of female samples based on genotype on the second component of the PCA plot (Figure 5A), suggesting that HHEX contributes to transcriptional programs in the female adrenals as well. Despite the low number of genes differentially expressed between *WT* and *Hhex KO* females, 21 out of 23 were common to DEGs in male *WT* and *Hhex KO*. This included 16 upregulated genes and 5 downregulated genes in *Sf-1-Cre*^*high*^ *Hhex KO* compared to their respective controls (Figure 6C). Among them, *Abcb1b* was the most downregulated gene upon *Hhex* deletion, and was further studied based on its established role in sterol flux and stress adaptation in the adrenal cortex^19,98^. Decreased *Abcb1b* expression was further confirmed in larger cohorts, at two time points, in both sexes, using RT-qPCR (35–80% decrease) (Figure 6D (6-week-old) and 6F (15-week-old)) and RNAscope (Figure 6E, left). Based on the decreased expression of *Abcb1b* upon *Hhex* deletion, we thus hypothesized that the expression of other genes uniquely expressed in the inner zF subpopulation might be downregulated with *Hhex* loss. In agreement with this hypothesis, we performed RT-qPCR on *WT* and *Hhex KO* male adrenals and observed a decrease of mRNA levels for other markers of these cells, such as *Srd5a2* and *Mgst2* at 6 weeks of age (Supplementary Figure 6a), suggesting HHEX is required for a unique gene expression profile in this population.

To clarify when HHEX is required for *Abcb1b* expression, we used *Cyp11b1-Cre*^*ERT2*^ *; Hhex*^*flox/flox*^ (*Hhex KO*) and *Cyp11b1-Cre*^*ERT2*^ *; Hhex*^*wt/wt or flox/wt*^ (*WT* and *Heterozygotes*) mice (Supplementary Figure 2b) fed a tamoxifen chow for 4 weeks after puberty. This strategy restricted the recombination to the fasciculata lineage upon tamoxifen administration and discriminated defects affecting embryogenesis and/or homeostasis. Similar to the *Sf1-Cre*^*high*^ model, we observed a significantly decreased expression of *Abcb1b* in the adrenal of *Cyp11b1-Cre*^*ERT2*^ *; Hhex*^*flox/flox*^ males and females compared to littermate controls (Supplementary Figure 6b), suggesting that HHEX is necessary for *Abcb1b* expression during homeostasis. This also confirmed that the function of HHEX in promoting *Abcb1b* expression was specific to the adrenal and not to the brain or gonad.

Based on our finding that HHEX promotes *Abcb1b* expression, we hypothesized that the previously described expansion of the *Abcb1b* population in response to chronic stress^19^ would be impaired in *Hhex KO* mice. To test this hypothesis, we used long-term ACTH administration as a surrogate for chronic stress exposure^99^ and performed an *in situ* single-molecule approach (RNAscope) for *Abcb1b*. As expected, ACTH induced hyperplasia and hypertrophy in *WT* mice (male and female) and led to significant increases in adrenal-body weight ratios in both sexes and both genotypes (Supplementary Figure 6c and 6d). Using RNAscope, we observed that the *Abcb1b* population was localized to the inner zF in *WT* adult mouse adrenals at baseline (Figure 6F and Supplementary Figure 6e), consistent with previous reports^19^. In females, *Abcb1b* expression was also found in cells of the X-zone (Supplementary Figure 6e). Consistent with other *in vivo* models of chronic stress, we observed a centrifugal expansion of the *Abcb1b* expression domain in the adrenals of both male and female *WT* mice, which demonstrates the robustness of this response upon HPA axis activation (ACTH response). At baseline (non-ACTH treated), the intensity of the *Abcb1b* staining was visibly reduced in both male and female *Hhex* KO adrenals (Figure 6F and Supplementary Figure 6e, left panels), which was confirmed and quantified by RT-qPCR (Figure 6G). Under chronic ACTH administration, the *Abcb1b* staining was also visibly reduced in *Hhex KO* compared to *WT* adrenals (Figure 6F and Supplementary Figure 6e, right panels). Surprisingly, chronic ACTH administration led to increased adrenal weight in *Hhex KO* despite a failure to increase *Abcb1b* expression, suggesting that expansion of the *Abcb1b* expression domain and zF hyperplasia are two partially uncoupled mechanisms. While the adrenal-specific role of *Abcb1b* is unknown in mice, ABCB1 has been shown to mediate GC release from human adrenal cells^19^ and *ABCB1* polymorphism (rs2032582) has been associated with higher cortisol secretion in cortisol-producing adrenal adenomas in humans^100^. Therefore, to assess whether HHEX is contributing to GC release during a chronic stress response, we harvested female *WT* and *Hhex KO* adrenals upon chronic ACTH administration (when *Abcb1b* expression is increased in both sexes). We measured corticosterone released into media by explants and found similar levels between female ACTH-treated *WT* and ACTH-treated *Hhex KO* adrenal explant (Supplementary Figure 6f). This observation suggests that in females, unlike in males, despite *Hhex* loss and a decrease in *Abcb1b* expression of 46%, residual *Abcb1b* expression may be sufficient to sustain corticosterone release under the conditions tested. **Taken together, these results demonstrate that in both sexes, HHEX is required to maintain optimal *Abcb1b* expression in the inner zF, at baseline and upon chronic stress.**

## Discussion

In the present study, we generated a single-cell RNA seq dataset of the adrenocortical steroidogenic lineage that revealed marked transcriptome heterogeneity in GC-producing zF cells. We identified the **homeobox HHEX** as the most enriched transcription factor in the **zF** and established its role in defining inner zF cell identity. We demonstrated that HHEX is required to **protect lipid droplets from androgen-induced lipid depletion** and that the loss of HHEX increases lipophagy, leading to decreased steroidogenesis and GC deficiency *in vivo* in males. We demonstrate that HHEX is a *bona fide* **target gene of the androgen receptor** and is highly expressed in the male adrenal, which allows for the repression of female-biased transcriptional programs. Finally, we show that HHEX is required for the expression of the **steroid and xenobiotic efflux transporter *Abcb1b*** at baseline and in response to chronic stress in both males and females.

Our scRNA-seq findings on cells of the steroidogenic lineage are consistent with prior work defining well-known markers of the zG (*Cyp11b2*, *Dab2*, *Lef1*, etc.) and zF (*Cyp11b1*). Further, our results strengthen recent observations that characterized a unique *Abcb1b*^*+*^*/Sbsn*^*+*^*/Srd5a2*^*+*^ population in the inner cortex, and reveal novel zone-specific regulators enriched in the zF (*Mmd2*, *Acsbg1* and *Abca1*) and zG (*Pp2r2b* and *Pcdh19*). While some prior studies have focused on adrenal development^101^, our study focused on the mouse adult steroidogenic lineage during homeostatic maintenance and identified the novel zF-specific regulator HHEX. HHEX is a transcription factor that belongs to the family of homeobox proteins that contribute to segmental identity, anterior/posterior patterning of the embryo, mammalian organogenesis, and adult tissue maintenance. Depending on cellular context and cofactor interactions, HHEX functions as a transcriptional repressor or an activator in a tissue-specific manner. In the mouse adrenal, we found that HHEX promotes the expression of *Abcb1b* in the zF but negatively regulates the expression of *Dax1 (Nr0b1)*. *HHEX* single nuclear polymorphisms (SNPs) have been repeatedly associated with Type 2 diabetes mellitus. Variants rs1111875 and rs7923837 are associated with impaired insulin secretion and decreased hepatic insulin degradation^102–105^. Other studies revealed a correlation between *HHEX* polymorphisms and metabolic parameters such as triglyceride (rs7923837) and total cholesterol levels (rs2488075 and rs947591)^106^. Relevant to our study, rs2497306 is negatively correlated with levels of DHEAS, a steroid solely produced by the inner human adrenal cortex^107^. While this observation supports our data that HHEX controls steroid output, current mouse models do not permit us to directly assess the role of HHEX in adrenal androgen production (DHEA) because the mouse adrenal cortex does not express cytochrome P450 17α-hydroxylase/17,20 (CYP17) after birth, an enzyme required for androgen synthesis^17^. Importantly, our study lays the groundwork for deciphering the participation of the adrenal gland in the phenotype associated with *HHEX* genetic variants.

In this study, we not only demonstrate that HHEX is required for GC production, but we also unmasked the critical role of lipophagy in adrenal lipid droplet catabolism. While the neutral cytoplasmic CE hydrolase HSL, encoded by *Lipe,* has been considered to be the main ester hydrolase in the adrenal^108,109^, our findings suggest that conditions exist under which LAL-mediated lipolysis is prevalent. Multiple lines of evidence support that LAL contributes to the regulation of adrenal lipid metabolism. In humans, it has been reported that LAL deficiency accounts for up to 3% of primary pediatric adrenal insufficiency^110,111^. More broadly, LAL mutations are responsible for autosomal recessive cholesteryl ester storage disease (OMIM #27800). The most severe form with complete absence of LAL activity, Wolman disease (OMIM #620151), is usually fatal in the first six months of life. Clinical findings include dyslipidemia, cirrhosis, and calcification of the adrenal^110,112–118^, hypothesized to be the accumulation of excess hydrophobic lipids that result in necrosis of adrenal cells and calcification^119^. Our findings that inhibition of lysosomes by HCQ-S partially rescues depletion of lipid droplets in male *Hhex KO* adrenals indicate that macrolipophagy^120^, an autophagosome-mediated degradation process, facilitate lipid catabolism in the adrenal cortex. Our results also implicate AR-HHEX signaling in regulating adrenal lipophagy and reveals the sex-specific role of androgen-promoting lipid depletion. Therefore, our mouse model opens an exciting new avenue of study focusing on the molecular mechanisms regulating cholesterol metabolism *via* lipophagy.

In steroidogenic cells, biosynthesis of steroid hormones utilizes cholesterol as a precursor, and in gonads, lipophagy contributes to sex steroid synthesis^121–123^. However, our finding of decreased corticosterone in *Hhex KO* adrenals suggests that lipophagy does not stimulate steroidogenesis. These results implicate lipophagy in the control of hormone synthesis and suggest that activation of lipophagy in the zF could limit adrenal hormone production by restricting cholesterol ester availability for steroidogenesis. Whether excess lipophagy contributes to clinically relevant adrenal insufficiency is unknown. However, cortisol excess is a common feature of adrenocortical carcinoma (ACC), an aggressive form of adrenal cancer, and has been repeatedly associated with poor prognosis among patients^124,125^. Impaired lipophagy and toxic lipid accumulation play a crucial role in the development and progression of multiple diseases, such as atherosclerosis^126–128^ and Metabolic Dysfunction-Associated Steatotic Liver Disease (MASLD)^129^. Activation of lipophagy and cholesterol efflux has been proposed as an attractive treatment strategy to prevent cholesterol plaque formation. Lipophagy-inducing compounds have been identified and generated^130^ with a number of moieties already clinically approved^131^. Additional studies will be necessary to assess the potential of lipophagy as a promising therapeutic approach to prevent cortisol overproduction in Cushing disease and mild autonomous cortisol secretion, and reduce comorbidities of GC-overproducing adrenal tumors.

We also identified a HHEX-dependent population of inner zF cells in male and female mice that express high levels of *Abcb1b* (also known as MDR1 and P-glycoprotein). Despite the human adrenal gland expressing the highest level of ABCB1, this efflux pump is best known for ensuring blood-brain barrier^98^ integrity by preventing the accumulation of toxic levels of xenobiotics and steroids. *Abcb1*-deficient mice exhibit elevated GC in the brain, which is speculated to suppress the HPA axis. Consistent with this notion, *Abcb1*-deficient mice present with lower corticosterone, ACTH and CRH levels^132^. In humans, polymorphism (rs2032582) in *ABCB1* is associated with decreased HPA axis response (GC levels)^19^ and pharmacological inhibition of ABCB1 activity decreases GC release from human adrenocortical cell lines^19^ suggesting a role for ABCB1B at the adrenal level in addition to the brain. These findings raise the question of whether HHEX variants account for physiological or subclinical variation in the stress response. It is also possible that ABCB1 protects adrenocortical cells from xenobiotics and drugs. The adrenal cortex expresses many detoxifying enzymes, such as the P450 enzymes, Aldo-Keto reductases, and glutathione transferases, particularly in the inner cortex. The overlapping expression of ABCB1 suggests multiple mechanisms exist to prevent the accumulation of cytotoxic compounds. In ACC, the lack of efficacy of chemotherapy is thought to be a consequence of increased expression of ABCB1^133^. *In vitro* ABCB1 inhibition, using Tariquidar or Veparamil, sensitized H295R, a human adrenocortical carcinoma cell line, to doxorubicin and etoposide^134^. Consequently, modulators of ABCB1 expression, such as HHEX, are promising targets for improving the cytotoxicity of current treatments of ACC and other multidrug resistant cancer type.

Finally, sex differences are increasingly considered major contributing factors to differences in adrenal disease manifestations, which are more common in females^135^. A more complete understanding of the genetic programs and regulatory factors orchestrating adrenocortical sexual dimorphism will lay the groundwork for future effective sexspecific therapies. The canonical androgenic pathway (via AR/NR3C4) is crucial for the masculinization of zonation and the male-specific transcriptome of the adrenal^24,28,96^. High expression of AR in the zF and weak expression in the zG^97^ were confirmed in our study, and sexual dimorphism was partially abolished upon *Hhex* deletion in males, despite sustained AR expression and normal levels of circulating androgens. *Hhex KO* and *ARKO* adrenals share many differentially expressed genes, and *Hhex* expression is driven by androgens, indicating that AR engages HHEX to mediate adrenal sexual dimorphism, at least in part by repressing the female transcriptional program. Among the shared differentially expressed genes, *Dax1* is a well-known female-biased gene with high expression in the entire female adrenal cortex and low expression in males (except in the zG)^97^. The *Dax1* expression pattern mirrors the expression of *Hhex* and *Dax1* is repressed at puberty precisely when HHEX expression is initiated. Following *Hhex* deletion, *Dax1* expression is increased in the male adrenal to levels comparable to the female adrenal, suggesting that HHEX is a crucial negative regulator of *Dax1* expression. Sex-biased expression of *Dax1* has been shown to be dependent on AR but independent from its binding to the *Dax1* promoter^97^. Instead, the suppressive action of AR was mainly achieved through SF-1 binding to the *Dax1* promoter. Considering our results, we propose that HHEX is part of this regulatory system, serving as a transcriptional repressor of SF-1-mediated *Dax1* expression in the zF. While the mouse adrenal cortex is recognized as one of the most sexually dimorphic non-reproductive organs, whether, and to what extent, this is the case in humans remains unknown. Indeed, contrary to rodents, humans of both sexes produce adrenal androgens, and in pathological conditions such as Congenital Adrenal Hyperplasia (CAH) and Polycystic Ovary Syndrome (PCOS), androgens can reach supraphysiological levels. Deciphering the mechanisms by which androgens coordinate sex differences in adrenocortical responsivity/stress adaptation will likely be informative for improved care. To our knowledge, this is the first report of the regulation of the expression of a homeobox protein by androgens in steroidogenic cells of the adrenal gland which expands the current knowledge on the regulation of homeobox expression by sex hormones. In conclusion, our work adds to the growing scientific consensus that the adrenal gland is an androgen-sensitive organ and now places HHEX as a major contributor.

To conclude, a better understanding of adrenal cell identity and differentiation is needed for the development of targeted therapy of glucocorticoid-related adrenal disorders. We analyzed cellular diversity in adrenal steroidogenic lineage in mice and identified multiple novel potential molecular regulators, notably HHEX, which was investigated using conditional knockout mice and cutting-edge chromatin accessibility molecular techniques. Our findings reveal that HHEX is crucial for maintaining plasma glucocorticoid levels at baseline in males. We also gleaned new mechanistic insights into the regulation (by HHEX) of the *Abcb1b*+ population, which is crucial for stress adaptation. Overall, our findings add to the existing knowledge involving the function of HHEX in differentiated cells. However, HHEX seems to have tissue-specific roles. Its involvement in cholesterol metabolism was completely unknown in the adrenal or any other organs until now. In the adrenal cortex, we demonstrated that HHEX contributes to androgen signaling and participates in the repression of the female transcriptional program in the adrenal. In parallel, its activity protects lipid droplets by preventing androgens from activating lipophagy and triggering lipid depletion in adrenocortical steroidogenic cells. Maintaining cholesterol storage integrity and homeostasis is crucial for adequate steroid release, and our work improves our understanding of the complexity of GC-producing cells. In particular, we lay the groundwork for deciphering the landscape of HHEX functions and provide potential clues to the regulation of lipophagy. Collectively, this information should facilitate cell-targeted therapeutics to treat Cholesteryl ester storage disease (CESD), and other disorders associated with excess lipid storage. Genome-wide association studies have identified variants of HHEX associated with type 2 diabetes, and our work suggests that genetic alterations or variants of expression could be associated with the sensitivity of the adrenal gland to stress response and androgen signaling. Finally, most adrenal diseases do not affect men and women equally. Preclinical biomedical studies have historically focused on male animals, leaving essential questions unanswered. The present study exemplifies how considering sex as a biological variable (SABV) can reveal fundamental molecular mechanisms regulating cellular processes such as lipophagy. We expect the generalization of including SABV when performing experimental studies will improve our understanding of adrenal disease mechanisms.

## Limitations of the study

Our research provides a comprehensive single-cell RNAseq dataset of the steroidogenic lineage in the adult male mouse adrenal. However, our downstream functional studies focused solely on HHEX, the identified top transcription factor enriched in the zF. Other proteins and transcription factors enriched in the zG, the zF, and the newly identified *Abcb1b*^*+*^ cell population remain to be further studied and fully characterized to improve our understanding of the molecular mechanisms modulating steroid production in the adult adrenal cortex. To investigate the role of HHEX in adrenocortical function, we combined genome profiling approaches and transgenic mouse models. Despite a lack of a gain-of-function approach, we generated and phenotyped for the first time adrenal-specific *Hhex KO* mouse models of both sexes and at multiple ages. However, *Hhex KO* mice may not inform us of all the possible roles of HHEX in the human adrenal, especially regarding adrenal androgen production. Indeed, functional reticularis cells are absent from rodent species commonly used in a laboratory. Therefore, animal models that better recapitulate human adrenal zonation and steroid production are required for more precise mechanistic and preclinical studies in the future. We also demonstrated that HHEX is expressed in the normal human adrenal of both women and men by immunohistochemistry, but the number of samples was limited and may not allow the accurate assessment of important differences in HHEX expression between sexes, ages, or hormonal status in humans. Finally, our results demonstrate for the first time that androgen signaling serves as a potent stimulating signal driving HHEX expression in the adrenal at puberty, but other hormonal factors, such as estrogens, might also influence adrenal functions and were not addressed in the current study.

## Material and Methods

### Ethical approval declaration

All experiments were carried out in accordance with protocols approved by the Institutional Animal Care & Use Committee (IACUC) at the University of Michigan, the guidelines set by the National Institutes of Health (NIH), and the three R rule (Replacement, Reduction, and Refinement). All efforts were made to minimize animal suffering and distress.

### Sex as a biological variable.

This study examined both male and female mice since sexual dimorphism was observed in the results and therefore studied in the current manuscript.

### Mice

Mice were bred in-house and maintained on a C57Bl/6 background at the University of Michigan, USA. They were housed on a 12h light/12h dark cycle (lights on at 6 am) and fed the commercial rodent chow 5L0D (PicoLab^®^ Laboratory Rodent Diet). Exceptions were made during breeding and before weaning when the Formulab Diet 5008 chow (LabDiet) was used, and during Cre^ERT2^ activation experiments, when mice were placed in a new cage and exclusively fed Teklad Custom TAM diet (400 TC, 2016 - TD.130859, Envigo, expected to provide ~40 mg tamoxifen per kg of body weight per day, assuming 20–25 g body weight and 3–4g intake). In all experiments, mice were provided water and food *ad libitum*. At weaning (around 3 weeks of age), mice were separated from parents and kept with same sex littermates at a maximum of five animals per cage.

*Hhex*^*flox/flox*^ mice were purchased at Jackson Laboratory (Stock 025396; B6N.129S1(Cg)-Hhex<tm2Cwb>/J; https://www.jax.org/strain/025396) and initially donated by Dr. Clifford Bogue, M.D., Yale University, USA^136^. They contained loxP sequences flanking exon 2–3 of the *Hhex* mouse gene (ENSMUSG00000024986). *Hhex*^*flox/flox*^ mice have been previously described and used to successfully delete *Hhex* in the liver^136^, pancreas^51,137^, osteoclast^138^, and myeloid lineage^139^. After cryorecovery, *Hhex*^*flox/flox*^ mice were crossed with *Sf1-Cre*^*high*^ *; mT/mG mice*, *AS-Cre; mT/mG,* and *Cyp11b1-Cre*^*ERT2*^; *mT/mG* lines. *Sf1-Cre*^*high*^ mice were provided by the late Dr. Keith Parker, M.D, Ph.D, University of Texas, USA^63^. Sf1-Cre allele harbors five copies of a transgene containing 111 kb of the *Sf1* locus to target Cre expression in various steroidogenic tissues including the adrenal cortex. This mouse line has been described multiple times to successfully delete various floxed alleles in the adrenal steroidogenic lineage^20,21,65,140,141,141–14720,21,26,30,65,66,140–144,144,146–148^. *AS-Cre (Cyp11b2-Cre)* mice were kindly shared by Dr. David Breault, M.D., Ph.D., Harvard, USA^27^, and were similarly used to delete various floxed alleles in the definitive cortex^20,24,66^. *Cyp11b1-Cre*^*ERT2*^ mice were developed and kindly shared by Dr. Felix Beuschlein, M.D, Zurich, Switzerland. The Cyp11b1-CreERT2 mouse line was generated by PolyGene AG (Rümlang, Switzerland) to establish a Cyp11b1-knockout mouse model with a tamoxifen-inducible Cre recombinase (Cre-ERT2) under the gene’s control. Exon 2 was selected for inserting the expression cassette, preserving potential regulatory elements in intron 1. To prevent alternative translation initiation, all five ATG start codons in exon 1 were mutated to CTG. The targeting vector included homology arms (4.97 kb and 1.83 kb) generated by PCR from C57Bl/6N BAC DNA, as well as Cre-ERT2 and β-globin poly(A) sequences assembled via overlap-extension PCR. Additionally, a 1.3 kb fragment containing mutated exon 1, intron 1, and part of exon 2 was synthesized and cloned into the short arm–Cre-ERT2 construct. The final targeting vector, H052.4 TV, incorporated an FRT-flanked neomycin selection cassette and was validated through sequencing. The following primers were used to select positive embryonic stem cell clones H052.17: binds to the elongated short arm of homology 5’- CACGCTGAACTAGCATAGCC - 3’ H052.19: binds to the Cre-ERT2 5’- CTACACCAGAGACGGAAATCCATC - 3’. *Rosa*^*mT/mG*^ reporter line^64^ has been described multiple times and successfully used to track recombined cells, including steroidogenic cells^20,24,26,27,30,65^. *AR*^*flox*^ mice^91^ were made at the Catholic University of Leuven, Belgium, and kindly shared by Dr. Frank Claessens and Dr. Johan Swinnen.

### Rats

Naïve rats were obtained from the animal/rodent redistribution program in place at the University of Michigan and euthanized with C0_2_ according to ULAM protocol. Rat adrenals were harvested, fixed, processed, and embedded similarly to the mouse adrenals.

### Human Adrenal Glands

Human adrenal samples were obtained from renal transplantation donors at the University of Michigan. Formalin fixed paraffin-embedded human adrenal samples without overt pathology based on histologic analysis were used to prepare 5-μm sections that were used for immunohistochemistry. Bulk human tissue gene expression for HHEX were obtained from the GTEx portal (GTEx Analysis Release V10 (dbGaP Accession phs000424.v10.p2).

### Genotyping

Mouse tail clips of 2 to 3 mm of length were harvested at weaning, and genomic DNA was extracted using the HotSHOT method^149^. Briefly, tissue was digested in 75 μl of Alkaline Lysis Reagent (25 mM NaOH, 0.2 mM disodium EDTA, pH of 12 without adjusting) for 30 min at 95°C. Samples were then chilled on ice for 2–3 min, and 75 μl of Neutralization Buffer (40 mM Tris-HCl, pH of 5 without adjusting) were added (Supplementary Table 1). 20 μL PCR reactions were set up in TempAssure 0.2 mL PCR 8 strips color (USA scientific, #1402–4708). For each PCR reaction, 2 μL of genomic DNA were added to a mix comprised of 10 μL of 2X GoTaq^®^ Green Master Mix (Promega, #M7123), combined with 1 μL of each primer (10 μM) and milli Q water for a final volume of 20 μL. Genotyping primer pairs used are listed in Supplementary Table 2. After amplification, PCR products were separated on 2% agarose gels dyed with SYBR Safe (ThermoFischer Scientific, #S33102).

### Hormonal manipulation, treatment, and surgery

Chronic ACTH treatment was performed using Synachten 1mg/mL diluted in PBS 1X (Gibco, #10010023) to 0.25mg/mL. 13 to 15-week-old male and female mice were injected twice a day intraperitoneally (50 μL) for 10 days. Adrenal enlargement was confirmed at dissection. Testicular gonadectomy was performed using 6-week-old *WT* and *Hhex KO* males. Animals were anesthetized with isoflurane (Fluriso, VetOne) and simultaneously injected with Carprofen (Rimadyl, diluted to 1mg/mL) at 5mg/kg for pain relief. Testes were surgically removed, and animals were placed individually in new clean cages after surgery, monitored twice a day for 7–10 days for recovery until suture clips were removed under brief isoflurane anesthesia. At dissection, the regression of seminal vesicles (androgen-sensitive organ) was confirmed, indicating the successful withdrawal of androgen production.

### *In vivo* hydroxychloroquine treatment

Hydroxychloroquine sulfate (HCQ-S, Tokyo Chemical Industry, #H1306) was first resuspended in di-ionized water at a concentration of 6mg/mL, aliquoted, and stored at −20°C. HCQ-S was then diluted to a concentration of 0.6mg/mL with room temperature deionized water (similar to control mice). The solution was administered *ad libitum* in clean water bottles every week. No other liquid source was available during the treatment duration. Based on average water intake, mice were delivered a daily oral dose of 2–4 mg of HCQ-S. Mice were monitored at least weekly.

### Tissue Harvesting

The day prior to euthanasia, each individual mouse was briefly put on a scale, and the body weight (in grams) was recorded to calculate the adrenal to body weight ratio after euthanasia. At dissection, left adrenal glands were placed in PBS 1X on ice until a precise dissection was performed under a stereo microscope (Nikon, #SMZ800) to remove surrounding fat. Weighing (in milligrams) and imaging (Olympus, #DP21) were quickly recorded to prevent tissue dehydration and lysis. Right adrenals were briefly harvested, dissected from surrounding fat, and immediately snapp frozen in liquid nitrogen for long-term storage at −80°C.

### Mouse adrenal single-cell dissociation and single-cell RNAseq

Adrenal glands were harvested from 15-week-old *SF1-Cre*^*high*^*; Rosa*^*mT/mG*^ male mice following rapid decapitation to reduce stress-induced transcriptional changes. Adrenals were placed immediately into ice-cold 1X Hank’s Balanced Salt Solution (HBSS 1X, Thermo Fisher 14025092) containing calcium and magnesium. 30–50mg of tissues were then finely chopped in 100–200 μL of HBSS 1X using single-edge razor blades (Persona GEM, #62-0179). From this point, all tips and tubes were precoated with 3% BSA (Roche, #3116956001) in PBS 1X (w/v) to prevent cell loss. Tissue pieces were then transferred to 5 mL tubes (Eppendorf, #0030119487) containing 2 mL of cold HBSS 1X supplemented with 10% Fetal Bovine Serum (Corning, 35–010-CV) and centrifuged for 5 min at 4°C at 500 g and supernatant was pipetted out and discarded. Single cell suspension was then obtained at low temperature (4 to 10°C) by combining enzymatic and mechanic dissociation according to the following steps. Mix 1, containing 1900 μL of Buffer X (Mitenyi Biotec, #130-092-628), 50 μL of Papain (Mitenyi Biotec, #130-092-628) and 20 μL of collagenase (Sigma-Aldrich, #C2139) prepared at a concentration of 100mg/mL in DMEM-12, was added to the tissue preparation and incubated for 15 min at low temperature. Mix 2 (10 μL of enzyme A and 20 μL of Buffer Y, Mitenyi Biotec, #130-092-628) was added to the digestive solution for 1h from this step. During the dissociation, the cell suspension was gently agitated with mechanical pipetting every 10 minutes and visually assessed under a microscope for 1 hour until the tissue was fully digested. The suspension was then filtered through 70 μm filters (PluriSelect USA, #43-10070-40) to obtain a single cell suspension, and enzymes were neutralized using HBSS 1X containing 10% Fetal Bovine Serum. Red blood cells were removed using Red Blood Cell Lysis buffer (Roche, #11814389001) according to manufacturer guidelines (15 min on orbital shaker in a cold room), and the cells were washed twice in HBSS containing 2% FBS before counting. After final centrifugation, cells were resuspended in 1 mL of HBSS 1X, FBS 2%. Following dissociation, cells were stained with 2 μg/ml DAPI and 5 μM Vybrant DyeCycle Ruby (Invitrogen, #V10309). Ruby-positive, DAPI-negative single cells were sorted based on fluorescent properties using the Thermofisher Bigfoot Cell Sorter at the University of Michigan Flow Cytometry Core. Cells were collected in BSA-precoated tubes containing HBSS 1X with 2% FBS and kept on ice. Single-cell droplets with a target capture of 10,000 cells were immediately prepared on the 10x Chromium system at the Advanced genomic core. Single-cell libraries were prepared using the Chromium Next GEM Single Cell 3′ Gene Expression Library Construction Kit version 3.1 according to manufacturer instructions. Sequencing was performed on an Illumina NovaSeq (S4) 300 cycle. Raw and processed data have been deposited in NCBI’s GEO database (GSE291472).

### scRNA-seq analysis, cluster identification and gene marker determination

Data of each individual experiment was analyzed according to the following steps: Raw sequencing data was aligned to the mouse reference genome (GRCm39) and quantified using cellranger count (10x Genomics). The resulting count matrices were processed in R (version 4.3.1) using Seurat (version 5.0.1)^150^. Low-quality cells, defined as those exhibiting a high percentage of mitochondrial reads or a low number of detected features were identified and removed using miQC^151^ (version 1.10.0). Doublets were identified and removed using DoubletFinder (version 2.0.4)^152^. Datasets from individual experiments were merged and normalized using the SCTransform function from Seurat. To account for technical variability, we regressed out the percentage of reads mapped to mitochondria and ribosomal genes during normalization. We performed integration of the datasets using harmony (version 1.2.0)^153^. Cell clusters were identified using the FindClusters function in Seurat using the Leiden algorithm (version 0.10.0)^154^. Uniform Manifold Approximation and Projection (UMAP) dimensional reduction was performed on the harmony-corrected embeddings using RunUMAP function in Seurat. Smoothed visualizations of gene expression in UMAP space was generated using Nebulosa (version 1.12.1), which employs kernel density estimates for enhanced visualization and interpretation^155^. Marker genes for each cluster were identified using the FindAllMarkers function in Seurat. Statistical significance was inferred using the Wilcoxon rank-sum test and adjusted for multiple testing using the Benjamini Hochberg method. The top 100 marker genes for each cell clusters are listed in Supplementary Figure 1a. Cortical cell clusters (defined by the expression of cortical cell specific marker Nr5a1) were further classified as ZG- or ZF-biased based on the expression of known ZG and ZF markers such as Vsnl1, Cyp11b2 (ZG), and Cyp11b1 (ZF). Grouped differential expression analysis between ZG- and ZF-biased clusters was performed using the FindMarkers function from Seurat. A volcano plot displaying the top 20 DEG genes was built using EnhancedVolcano (version 1.18.0). A CLOUPE file was generated from the Seurat-processed single cell gene expression dataset using the 10x Genomics’ LoupeR (version 1.1.3) for interactive and visualization and exploration, and available as a supplementary file.

### Hormonal measurements

When hormone measurements were expected to be performed, mice were sacrificed by decapitation in compliance with IACUC protocol between 8:30 and 10 am within 30 sec of handling to minimize stress-induced ACTH secretion. Core trunk blood was collected using sodium heparin-coated tubes (BD Vacutainer^®^ Heparin Tubes, #367871) and centrifuged at 1800 g for 20 min at 4°C to obtain plasma. Samples were divided into two aliquots for ACTH and steroid measurement and stored at −80°C prior to analysis. Corticosterone concentration was determined by liquid chromatography- tandem mass spectrometry (LC-MS/MS) as described previously^20,156^.

### Intra-adrenal Cholesterol esters and Free cholesterol measurement.

For each mouse, both adrenals were harvested and placed in cold PBS 1X, removed fat and weighed for downstream calculation and homogenized in 600 μL of lipid extraction solution (Chloroform, Isopropanol, Igepal) as described by manufacturer (Abcam, #ab65359) with slight modifications. Adrenal tissues were homogenized using a BeadBug^™^ Microtube Homogenizer (2 × 30 sec 400 (x10 speed)). Lysate was transferred into new 1.5 mL Eppendorf tubes and evaporated for 4 hours at 50°C in a chemical hood, then vacuum centrifuged for 30 min. Lipids were finally resuspended in 200 μL of assay buffer. Cholesterol levels were measured using two volumes that were preliminary determined to be in the range of the standard curve (5 and 10 μL for total cholesterol and 25 and 50 μL for Free cholesterol). The average quantity of cholesterol ester in *WT* adrenals was 9.6ug/mg of adrenal gland, similar to published literature^157^.

### *Ex vivo* mouse adrenal explant culture

#### DHT stimulation.

Seven-month-old female adrenals were quickly harvested after euthanasia and put in ice-cold PBS 1 X. They were then cut in half under a tissue culture hood, in a tissue culture dish, using sterile single-edge razor blades (Persona GEM, #62–0179). In a 24 well plate (USA Scientific, #CC7682–7524), two half adrenals were placed in each well in 1 mL of DMEM-F12 (GIBCO, #11330032) supplemented with 1% insulin-transferrin-selenium (GIBCO, #51500056) and 1% Penistrepto (GIBCO, #15140122). No serum was added to avoid exogenous steroid contamination. After 24h, explants were incubated with Vehicle or 5alpha-dihydrotestosterone (DHT) at 0.1 μM (Cerilliant, #D-073-1ML) for 48 hours. RNA was extracted from explants using the Mini RNA extraction kit (RNeasy Mini Kit Qiagen, #74104).

#### Dibutyril cAMP stimulation (Bt2-cAMP).

Dibutyryl-cAMP (sodium salt) (Sigma-Aldrich, D0260 and Cell Signaling #35857) was resuspended in sterile water at a concentration of 0.1mg/μL and used at a final concentration of 2.5 mM. After dissection, two half adrenals were placed in each well in 1 mL of prewarmed DMEM-F12 (GIBCO, #11330032) supplemented with 1% insulin-transferrin-selenium (GIBCO, 51500056) and 1% Penistrepto (GIBCO, #15140122) for 1 hour. No serum was added to avoid exogenous steroid contamination. Adrenals were then transferred into new wells and incubated with Vehicle (water) or Bt2-cAMP for the time indicated in the figures. Media was harvested at the end, and 2.5 μL of culture media was diluted 100 times in DMEM F12 and used for ELISA assays. Corticosterone levels (ENZO, ADI-900–097) were assayed in duplicate according to manufacturer instructions. In this experiment, samples were not treated with the steroid displacement reagent.

### Histology

Left adrenals were quickly transferred to be fixed in Formalin (FischerBrand, #427–098) for 24 hours at room temperature under gentle agitation on an orbital shaker. The next day, adrenals were rinsed in PBS 1X for 10 min at room temperature and transferred in 70% ethanol until processing. Tissue processing was performed on a Leica ASP 300S Tissue Processor at the UMICH Orthopaedic Research Laboratories (ORL) Histology Core (15 min per station for adrenal, 1h for larger tissues). Tissues were embedded in paraffin (Leica Paraplast, #39601006), and 5 μm sections were obtained using a HistoCore BIOCUT microtome (Leica) on Superfrost^™^ Plus Microscope Slides (Fischer Scientific, #12-550-15). A minimum of two serial sections were placed on each slide, thus allowing the inclusion of negative controls for each biological replicate. Sections were first baked for 45 min-1 hour in an incubator at 55°C, then deparaffinized in histoclear (National Diagnostics, #HS-200) (2 times-5 min) and rehydrated in graded ethanol (2 times-5 min in absolute ethanol; 2 times-5 min in 95%; 1 time-5 min in 70%; 1 time-5 min in 50%). After 5 min under running tap water, slides were immersed in antigen retrieval according to the Supplementary Table 3. After 3 times-5 min washes in PBS 1X, slides were incubated in hydrogen peroxide (Sigma-Aldrich, #H1009, 0.3% v/v in water), for 30 min at room temperature to inhibit endogenous peroxidase. Hydrophobic barriers were drawn around each section with an ImmEdge pen (Vector Laboratories, #H-4000). After 3 times-5 min washes in PBS 1X, slides were blocked using 2.5%, ready to use, Horse or Goat Serum (Vector Laboratories, #S-2012–50, #S-1012–50) depending on the on the host specie of the secondary antibody. When antibody made in mouse was used, the M.O.M.^®^ (Mouse on Mouse) ImmPRESS^®^ HRP (Peroxidase) Polymer Kit (MP-2400) was used according to manufacturer instructions. Adrenal sections were then incubated overnight at 4°C in a humid chamber with primary antibodies Supplementary Table 3. The following day, sections were rinsed 3 times-5 min in PBS 1X and incubated with appropriate secondary antibody (Vector Laboratories, Immpress HRP) for 30 or 10 minutes according to the manufacturer instructions (details in Supplementary Table 3). After 3 times-5 min washes in PBS 1X, staining was developed with ImmPACT^®^ DAB EqV Substrate Kit, Peroxidase (HRP) (Vector Laboratories, #SK4103). Sections were then rinsed 3 times-5 min in PBS 1X and 1 time-5 min under running tap water. Hematoxylin staining (Sigma-Aldrich, #GHS132–1L) was then performed to visualize nuclei before mounting with Permount^™^ Mounting Medium. Images were acquired on a Nikon Optiphot-2 with an Olympus DP70 camera and the DP manager software. For PLIN1 quantification of integrated density we used Fiji/ImageJ software (2.14.0/1.54f/Java 1.8.0_66)^158,159^ with the following parameters (Hue: 0–250, Saturation: 0–255, Brightness: 150–255, Thresholding method: Default, Threshold color: B&W, Color Space: HSB, Dark Background)

#### Costaining.

After HHEX staining was developed and sections rinsed 3 times-5 min in PBS 1X, slides were incubated in HCL 0.02N in water for 20 min to inhibit peroxidase from the Immpress HRP secondary antibody. Sections were then rinsed 3 times-5 min in PBS 1X and blocked as previously described, and incubated with DAB2 antibody overnight at 4°C in a humid chamber. The following day, the secondary antibody was applied as previously described, and staining was developed using the ImmPACT^®^ VIP Substrate Kit, Peroxidase (HRP) (Vector Laboratories, #SK-4605).

#### Visualization of endogenous fluorescence.

All steps were performed using dark tube or foil-covered plates to minimize exposure to light. At dissection, left and right adrenals were quickly harvested, dissected from surrounding fat, and incubated for 2 hours in 4% PFA at 4°C, then incubated in Sucrose 30% (Sigma-Aldrich, #84097) diluted in PBS 1X overnight at 4°C. The next day, adrenals were transferred into new tubes and incubated in Tissue-Plus^™^ O.C.T. Compound (Fisher Healthcare, 23-730-571) for 1 hour at 4°C. Finally, adrenals were transferred in cryomold (Tissue-Tek, 4565) and embedded in O.C.T. on a dry ice ethanol bath. The blocks were stored at −80°C until cryosection. The day of imaging, 5–7 μm thick sections were made on a cryostat (Leica CM 3050S) on Superfrost^™^ Plus Microscope Slides (Fischer Scientific, #12-550-15), incubated for 10 min in Hoechst (1:10 000 in PBS 1X) for nuclei visualization then mounted in a PBS/glycerol 50% (v/v).

### *In situ* single-molecule hybridization, *RNA scope*

Adrenals were fixed in 10% normal buffered formalin (VWR) for 24 h at room temperature, rinsed in PBS1X for 10 min, and paraffin-embedded, and cut into 5 μm sections. The RNAscope^™^ 2.5 HD Reagent Kit-BROWN (Advanced Cell Diagnostics, #322300) was used according to the manufacturer’s instructions and detailed previously^20^. Probes are listed in Supplementary Table 4. Images were acquired on a Nikon Optiphot-2 with an Olympus DP70 camera.

### *In situ* neutral lipid staining, *Oil red O*.

Oil red O stock solution was prepared by adding 1.25g of ORO (Sigma-Aldrich, #00625) to 200 ml of 99% (vol/vol) isopropyl alcohol (Sigma-Aldrich, #I9516), and the solution was mixed with magnetic stirring for 2 hours at room temperature. Snap-frozen adrenals were embedded in O.C.T. compound directly in mold in the cryostat’s chamber. Organs were kept frozen during the entire process, and 5–7 μm thick sections were made on a cryostat (Leica CM 3050S) on Superfrost^™^ Plus Microscope Slides (Fischer Scientific, #12-550-15). Prior to storage at −80°C, sections were dried at room temperature for 10 min to prevent detachment from the slides. Oil Red O working solution was prepared the day of the staining by adding 1.5 parts of ORO stock solution to one part of distilled water (30 ml of ORO to 20 ml of water). The solution was left at 4°C for 10 min to thicken before filtration through a 45 μM filter (Corning, #430627) to remove precipitates. In the meantime, slides were equilibrated for 10 min at room temperature, then incubated for 5 min in Oil Red O working solution before being rinsed for 30 min with running tap water. The slides were finally mounted in PBS:Glycerol (Fischer chemical, #G33) solution (1:1), let sit for 10 min at room temperature and sealed using dots of Permount^™^ Mounting Medium on the edge of the coverslip (Corning, #2980–225).

### RNA expression analysis

The right adrenals were quickly harvested after euthanasia, fat was quickly removed, and adrenal tissues were transferred into a tube, snap frozen, and stored at −80°C. At the time of RNA extraction, each adrenal was quickly transferred into a Lysing Matrix D 2 mL tube (MP Biochemicals, #6913100) containing 600 μL of RLT lysis Buffer (RNeasy Mini Kit Qiagen, 74104) supplemented with 6 μL of 2-mercaptoethanol (Sigma Aldrich, M7522). Lysis was performed using a BeadBug^™^ Microtube Homogenizer (2 × 30 sec 400 (x10 speed)). Lysat was transferred into new 1.5 mL tubes and an equal volume of 70% ethanol was added and mixed by pipetting. RNA was extracted according to manufacturer instructions with few modifications as follow to avoid contamination with Thiocyanate Guanidine in the final eluted sample. New collection tubes were used after each step of centrifugation so that the exterior of the column never came in contact with previously discarded flowthrough. Additional collection tubes were made by cutting the lids of 2 mL centrifuge tubes (USA scientific, #1620–2700). An additional step of centrifugation was added after the RW1 step to ensure complete removal from the column. When rinsing the column with 500 μL of RPE, the inside of the lid and the inner wall from the top of the column were rinsed during both washes. An additional centrifugation step was performed for 1 min at full speed (12 000g) to completely dry the membrane before elution. RNAs from male adrenals were eluted in 20 μL of water and 30 μL for female adrenals. RNA quality and concentration were assessed using a nanodrop (expected 260/280 ratio >1.8 and 260/230 ratio 2–2.2). RNAs were then stored at −80°C until retrotranscription or library preparation for RNAseq. For RT-qPCR, 500ng of RNA were treated with DNAse (Invitrogen, #18068–015) according to manufacturer instructions, and retrotranscribed into cDNAs at a final volume of 20μL using the High-Capacity cDNA Reverse Transcription Kit (Applied Biosystems, #4368814). cDNAs were diluted 10 times in water, and 2 μL were used for qPCR which was performed in MicroAmp Endura Plate Optical 96 well fast clear (Applied Biosystems, # 4483485), using the Power SYBR Green PCR Master Mix (Applied Biosystems, # 4367659) in a final volume of 10uL. Relative gene expression was determined using the ΔΔCt method using *Actb* (Actin beta) as a housekeeping gene and averaging duplicates for each biological replicates. Primer pairs are listed in Supplementary Table 5. Adrenal gene expression profiles for four 6-week-old *Sf1-Cre/+; Hhex*^*fl/fl*^ and four *WT* littermates were analyzed by bulk RNA sequencing. RNA samples were similarly treated with DNAse, and quality was evaluated by Bioanalyzer 2100 (Eukaryote Total RNA Nano chip, Agilent) at the Advanced Genomic Core at the University of Michigan (RIN > = 7.50 and free of genomic DNA contamination). Libraries were prepared from total RNA using standard poly(A) capture-based protocols and sequenced in paired-end mode on an NovaSeq (S4) 300. Sequencing quality metrics, including base quality scores and read count, were assessed using FastQC. Adapter sequences and low-quality bases were trimmed using the bbduk tool from BBTools. Processed paired-end reads were aligned to the mouse transcriptome sequence (GENCODE release M28, obtained from https://www.gencodegenes.org) using Kallisto^160^. Transcript-level abundance estimates were summarized to gene-level expression values using the lengthScaledTPM method in tximport^161^. Downstream analyses were performed in R using Bioconductor packages. To account for differences in library size, gene-level expression data were normalized using the Trimmed Mean of M-values (TMM) method implemented in edgeR^162^. Lowly expressed genes were filtered out using the filterByExpr function from edgeR to retain only genes with biologically meaningful expression levels. Unwanted and hidden sources of variation, such as batch effects, were removed using the sva package^163^. Principal component analysis (PCA) was performed using the standard prcomp function in R, and biplots were constructed using ggplot2. Differential gene expression analysis was performed using limma^164^. Raw and processed data have been deposited in NCBI’s GEO database (GSE291472). Common differentially expressed genes between 6-week-old male Hhex KO compared to their respective littermate WT and 25-week-old male ARKO compared to their respective littermate WT are available in Supplementary Figure 5f.

### Genome-wide profiling of DNA-binding proteins, Cleavage Under Targets and Tagmentation (CUT&Tag-IT^™^)

CUT&Tag-IT^™^ Assay Kit – Tissue (Active Motif, #53170) was used according to manufacturer instructions. Briefly, fresh adrenals from 6/9-week-old *WT* males were quickly harvested after euthanasia, cleaned from fat and transferred into ice-cold PBS 1X until being weighed. 10mg of tissue (4 to 6 adrenals) was used for nuclei preparation including the following conditions: no primary antibody, H3K27ac, and AR. For each condition, 20 μL of concanavalin A beads were prepared at room temperature according to manufacturer instructions. Briefly, 10 mg of tissue was lysed in 1 mL of Lysis buffer, and nuclei were extracted using a 1 mL dounce homogenizer. Lysate was filtered through 40 μm strainer, and nuclei were pelleted by centrifugation at 4°C. A range of 127 000–170 000 nuclei per mg of male mouse adrenals were obtained, and 280 000 nuclei per conditions were used. Nuclei were then bound to concanavalin A beads and incubated at 4°C overnight with primary antibodies (Supplementary Table 6). The following day, guinea pig anti-rabbit secondary antibody was bound to primary antibodies followed by an incubation with assembled pA-Tn5 transposomes. Tagmentation was then achieved by incubating samples at 37°C for 1 hour. DNA was then solubilized, purified and eluted. 30 μL of tagmented DNA was used to prepare libraries. Libraries were sequentially purified using SPRI Bead clean up from the kit, and AMPure XP Reagent (Beckman Coulter, #A63880) according to the manufacturer’s instructions. The quality of the library was assessed on a Bioanalyser at the Advanced Genomic Core at the University of Michigan. Libraries were sequenced on an Illumina NextSeq 500 platform using the P1 100-cycle kit in paired-end mode. Raw sequencing reads were processed using the following bioinformatics pipeline. Read preprocessing: Paired-end reads were interleaved using seqtk merge (https://github.com/lh3/seqtk). Adapter sequences and low-quality bases were trimmed using fastp (https://github.com/OpenGene/fastp) with default parameters. Quality control reports, including read length distribution, base quality and adapter content, were generated in HTML format. Alignment: Preprocessed reads were aligned to the mm10 mouse reference genome using Bowtie2 (version 2.3.5.1) with the following parameters: --local –very-sensitive –no-mixed –no-discordant -I 10 -X 700 to ensure high sensitivity for local alignment while preventing discordant and mixed alignments. Peak calling: Peaks were called using Genrich (https://github.com/jsh58/Genrich), with the -j option to enable ATAC-seq mode which adjust for the insertion bias caused by the Tn5 transposase. To visualize genome-wide signal distributions, BigWig files were generated from the aligned BAM files using the bam_to_bigwig function from the GenomicAlignments package in R. Raw and processed data have been deposited in NCBI’s GEO database (GSE291472).

### Software and applications

Graphs were generated using GraphPad Prism (10.2.2). Illustrations and Figures were created using PowerPoint (Version 2411 Build 16.0.18277.20082), the free life science icon library from Biorender.com, and R for the UMAP, the Inferno plots and the Volcano plot. The plugin Grammarly for Microsoft Word was used for grammar and spell-checking purposes at writing and editing stages of the manuscript. Literature references were added using the Zotero app (7.0.13).

### Statistics, scientific rigor and reproducibility

GraphPad Prism (10.2.2), R and G*Power (Version 3.1.9.7) were used for statistical analysis. Details related to the statistical test, number of biological replicates, mean, standard deviation, t-values, degree of freedom, F-value, power and *p*-value are available in Supplementary Table 7. No data were excluded from the analyses. A *p*-value <0.05 was considered significant. Exact p-values of interest are reported on each graphs together with appropriate star symbols (* <0.05, **<0.01, *** <0.001, **** <0.0001). When comparing two independent groups, we assessed the normality of the data by performing a Shapiro-Wilk test. If passed, a two-tailed unpaired t-test was performed to compare the means with a Welch correction to address issues related to unequal variances (F value ≠1), and differences in sample size between groups. When data were not passing the normality test, a Mann-Whitney test was applied to compare the ranks. Sample size was determined based on power calculations performed using G*Power to obtain >80% power with 5% type 1 error. When the p-value was <0.05, but 80% power could not be reached, an orthogonal approach was employed (detailed in Supplementary Table 7). For more than two-groups, we performed an ANOVA, followed by a Šídák’s multiple comparisons test. For histology, samples were blinded before analysis whenever possible, however in some cases, the genotype was evident from the images based on the adrenal lipid droplet content. When possible, orthogonal methods were used to ensure reproducibility of the results. For example, gene of interest identified by scRNAseq and bulk RNAseq were confirmed by RT-qPCR and/or immunohistochemistry on larger cohorts.

## Supplementary Material

1

## Data Availability

All sequencing datasets generated in this study have been deposited in the Gene Expression Omnibus (GEO) database under the accession code GSE291472.

## Highlights:

1. Single cell characterization of the male adrenal steroidogenic lineage leads to the identification of new transcriptional regulators.
2. Deletion of *Hhex* in the adrenal cortex results in lipid depletion and glucocorticoid insufficiency in males through lipophagy.
3. HHEX is a *bona fide* androgen receptor target gene in the adrenal *zona fasciculata* (zF), and its expression increases at puberty.
4. HHEX represses the expression of the female-biased gene *Nr0b1* (Dax1) at puberty in males.
5. HHEX is a master regulator of inner zF terminal differentiation, driving ABCB1B expression in this cell population.

## ABBREVIATIONS

ACTH: Adrenocorticotropic Hormone
AR: Androgen Receptor
CUT&Tag: Cleavage Under Targets and Tagmentation
DEGs: Differentially Expressed Genes DHT: Dihydrotestosterone
FACS: Fluorescence-activated cell sorting
GC: Glucocorticoids
GWAS: Genome-Wide Association Studies
HHEX: Hematopoietically Expressed Homeobox
HPA: Hypothalamic-Pituitary-Adrenal
KO: Knockout
LD: Lipid droplets
PRH: Proline-Rich Homeodomain protein
SNP: single nucleotide polymorphism
WT: Wild Type
zF: zona Fasciculata
zG: zona Glomerulosa
scRNA-seq: single-cell RNA sequencing
