# Supplementary material for: The transcription factor HHEX maintains glucocorticoid levels and protects adrenals from androgen-induced lipid depletion
Source: Res Sq. 2025 Apr 15:rs.3.rs-6248794. Preprint. [Version 1] doi: 10.21203/rs.3.rs-6248794/v1 (PMC12047992; doi:10.21203/rs.3.rs-6248794/v1)
Supplement: 1 [file NIHPPRS6248794V1-supplement-1.pdf]

Dumontet et al. 2025: Supplementary Figure 1

1a

Top 100 marker genes – Page 1/2– Complete list on Supplementary Table 7

Cycling Populations
Ranked by avg_Log2FC (adjusted pvalue <0.05)

cluster	gene	avg_log2FC	p_val_adj	cluster	gene	avg_log2FC	p_val_adj
CYC.1	Fbxl2	7.92	3E-243	CYC.2	E2f7	11.01	0E+00
CYC.1	Pomt1	7.85	0E+00	CYC.2	Clspn	8.57	0E+00
CYC.1	Copa	7.50	2E-231	CYC.2	Uhrf1	8.26	0E+00
CYC.1	Kcnk2	7.11	6E-165	CYC.2	Cdc6	8.25	0E+00
CYC.1	H2ac24	7.04	2E-276	CYC.2	Esco2	8.09	0E+00
CYC.1	Nxt2	6.97	0E+00	CYC.2	Lrr1	7.90	0E+00
CYC.1	Hirip3	6.95	9E-220	CYC.2	Ccne2	7.77	0E+00
CYC.1	Nudc	6.87	6E-113	CYC.2	H2ac10	7.77	1E-194
CYC.1	Psma6	6.84	2E-126	CYC.2	Pclaf	7.71	0E+00
CYC.1	Rnf138rt1	6.77	4E-236	CYC.2	Rrm2	7.60	2E-230
CYC.1	E230001N04Rik	6.75	2E-146	CYC.2	Tk1	7.51	0E+00
CYC.1	Ak2	6.66	4E-145	CYC.2	Dtl	7.49	0E+00
CYC.1	Gabrb3	6.57	3E-133	CYC.2	Exo1	7.46	0E+00
CYC.1	Cbx1	6.54	6E-64	CYC.2	Kntc1	7.37	0E+00
CYC.1	Tsn	6.53	4E-124	CYC.2	Neil3	7.22	0E+00
CYC.1	Lama5	6.51	3E-290	CYC.2	E2f8	7.17	0E+00
CYC.1	Maged2	6.45	6E-142	CYC.2	H3c2	7.14	4E-99
CYC.1	Sec22b	6.33	5E-153	CYC.2	H2ac22	7.07	1E-127
CYC.1	Chtop	6.33	1E-246	CYC.2	Rad51ap1	7.05	0E+00
CYC.1	1700037C18Rik	6.30	0E+00	CYC.2	Tcf19	6.96	0E+00
CYC.1	Atp6ap2	6.26	4E-225	CYC.2	H2ac11	6.94	5E-92
CYC.1	Cad	6.23	6E-191	CYC.2	Mxd3	6.87	3E-198
CYC.1	Palld	6.20	3E-130	CYC.2	Shisa8	6.86	3E-140
CYC.1	9530068E07Rik	6.18	3E-239	CYC.2	Haspin	6.84	6E-207
CYC.1	Insc	6.12	4E-126	CYC.2	Gm2788	6.79	5E-92
CYC.1	Sh3kbp1	6.04	2E-173	CYC.2	Dscc1	6.77	0E+00
CYC.1	Tmem39a	6.02	1E-154	CYC.2	Hrob	6.74	3E-237
CYC.1	9630002D21Rik	5.95	2E-160	CYC.2	Melk	6.74	0E+00
CYC.1	Blmh	5.93	1E-169	CYC.2	Ercc6l	6.60	0E+00
CYC.1	Tomm20l	5.89	2E-164	CYC.2	Top2a	6.55	0E+00
CYC.1	Bspry	5.87	5E-171	CYC.2	Asf1b	6.55	8E-277
CYC.1	Lzic	5.86	3E-120	CYC.2	Cdca5	6.54	2E-219
CYC.1	Gm17249	5.85	2E-175	CYC.2	Rad51	6.51	5E-303
CYC.1	Sptssa	5.85	0E+00	CYC.2	Brca1	6.51	0E+00
CYC.1	Ckap5	5.84	1E-135	CYC.2	H3c3	6.41	3E-136
CYC.1	Gpc4	5.78	1E-239	CYC.2	Rad54b	6.40	0E+00
CYC.1	D130040H23Rik	5.76	2E-132	CYC.2	Fanci	6.39	1E-291
CYC.1	Tmod3	5.73	7E-196	CYC.2	Chaf1b	6.38	0E+00
CYC.1	Ttf1	5.72	2E-83	CYC.2	Tex15	6.36	1E-140
CYC.1	Adprh	5.69	3E-123	CYC.2	Ankle1	6.34	2E-115
CYC.1	Fbxl8	5.63	7E-167	CYC.2	Bard1	6.26	1E-270
CYC.1	Znrf3	5.60	4E-152	CYC.2	Mybl2	6.23	2E-221
CYC.1	Wdr19	5.60	5E-210	CYC.2	H2ac24	6.22	9E-30
CYC.1	Cd1d1	5.44	5E-84	CYC.2	Kif15	6.21	2E-209
CYC.1	Fbxo5	5.40	9E-69	CYC.2	Ndc80	6.21	7E-293
CYC.1	Rilpl2	5.39	2E-137	CYC.2	Brip1	6.19	0E+00
CYC.1	ENSMUSG000001	5.38	2E-116	CYC.2	Ttk	6.18	3E-234
CYC.1	Ccdc122	5.37	2E-210	CYC.2	Ccne1	6.16	3E-191
CYC.1	Fbxo34	5.33	1E-85	CYC.2	Myh7b	6.14	2E-118
CYC.1	Pim2	5.33	2E-301	CYC.2	Aurkb	6.12	4E-191
CYC.1	Cadps	5.31	1E-73	CYC.2	Pole	6.09	0E+00
CYC.1	Atrnl1	5.25	1E-76	CYC.2	Mcm10	6.05	0E+00
CYC.1	Gm43305	5.21	5E-91	CYC.2	Cdt1	5.98	0E+00
CYC.1	Rere	5.15	2E-54	CYC.2	Shcbp1	5.95	4E-225
CYC.1	Prpf3	5.14	2E-117	CYC.2	Kif4	5.94	4E-225
CYC.1	Paqr8	5.13	3E-82	CYC.2	Prim1	5.93	0E+00
CYC.1	Cpa2	5.11	4E-187	CYC.2	Ska1	5.93	4E-170
CYC.1	Ten1	5.10	3E-109	CYC.2	Iqgap3	5.93	4E-172
CYC.1	Gm9899	5.08	5E-62	CYC.2	Phf19	5.92	2E-165
CYC.1	Sumo3	5.07	1E-138	CYC.2	Depdc1b	5.92	5E-133
CYC.1	Tusc3	5.06	6E-150	CYC.2	Cenpm	5.88	0E+00
CYC.1	Dusp19	5.04	1E-207	CYC.2	Bub1	5.87	3E-186
CYC.1	Ccdc148	5.03	6E-84	CYC.2	Fanca	5.84	5E-236
CYC.1	Wdr31	5.02	4E-109	CYC.2	2810408I11Ri	5.83	6E-208
CYC.1	Zc3h13	4.99	2E-55	CYC.2	Map1a	5.79	2E-137
CYC.1	Parvb	4.98	2E-93	CYC.2	Arhgef26	5.77	6E-136
CYC.1	Ndufaf3	4.97	8E-56	CYC.2	Diaph3	5.70	4E-233
CYC.1	Ccdc192	4.97	2E-112	CYC.2	Cenpk	5.68	3E-260
CYC.1	2010320M18Rik	4.93	1E-50	CYC.2	Pkmyt1	5.68	2E-234
CYC.1	Gm16063	4.93	2E-70	CYC.2	Mis18bp1	5.65	1E-209
CYC.1	Prpf38a	4.89	3E-129	CYC.2	Ncapg	5.64	8E-205
CYC.1	Map2k6	4.89	2E-102	CYC.2	Ncaph	5.62	2E-211
CYC.1	Abca6	4.83	4E-74	CYC.2	Ube2t	5.61	3E-210
CYC.1	Sgcb	4.82	2E-170	CYC.2	Eme1	5.60	4E-109
CYC.1	Cptp	4.68	1E-225	CYC.2	Spc25	5.58	7E-178
CYC.1	5530601H04Rik	4.68	3E-34	CYC.2	Spc24	5.58	3E-202
CYC.1	Eif4e3	4.67	2E-95	CYC.2	Eldr	5.57	1E-302
CYC.1	Gnb2	4.61	4E-43	CYC.2	Cdc45	5.54	8E-265
CYC.1	Fuca1	4.61	6E-67	CYC.2	Pbk	5.45	1E-217
CYC.1	Ptgfrn	4.57	2E-34	CYC.2	Slc43a3	5.45	3E-111
CYC.1	9330159F19Rik	4.56	1E-87	CYC.2	Cnih2	5.44	8E-91
CYC.1	Nkapd1	4.56	1E-108	CYC.2	Cenph	5.43	1E-246
CYC.1	Snrnp25	4.55	8E-169	CYC.2	Cit	5.40	1E-196
CYC.1	Comt	4.55	7E-69	CYC.2	Fignl1	5.40	2E-292
CYC.1	Col16a1	4.53	6E-60	CYC.2	Nusap1	5.38	1E-89
CYC.1	Cenpj	4.52	8E-138	CYC.2	Cdk1	5.37	1E-141
CYC.1	Jpt2	4.51	9E-95	CYC.2	Kif11	5.34	2E-127
CYC.1	Gipc1	4.49	6E-109	CYC.2	Cdca2	5.30	1E-106
CYC.1	Pla2r1	4.47	8E-98	CYC.2	Ticrr	5.26	3E-117
CYC.1	Pdia6	4.45	4E-50	CYC.2	Knl1	5.26	1E-126
CYC.1	Mcrs1	4.44	2E-44	CYC.2	Atad2	5.25	4E-105
CYC.1	Gabrg3	4.42	3E-40	CYC.2	4930579G24F	5.25	3E-140
CYC.1	Chst2	4.41	4E-97	CYC.2	Oip5	5.25	2E-151
CYC.1	Fmn1	4.37	4E-93	CYC.2	Fancd2	5.24	9E-137
CYC.1	Cpox	4.33	1E-52	CYC.2	Ncapg2	5.24	2E-135
CYC.1	Socs1	4.26	3E-88	CYC.2	Gm56727	5.24	4E-54
CYC.1	Rhbdl3	4.24	1E-47	CYC.2	Nsl1	5.23	5E-59
CYC.1	Thoc6	4.23	2E-42	CYC.2	H1f5	5.21	7E-40
CYC.1	Rbfox3	4.21	6E-31	CYC.2	Cenpu	5.20	2E-202
CYC.1	Gm49490	4.18	4E-46	CYC.2	Sgo1	5.19	2E-228

zG populations
Ranked by avg_Log2FC (adjusted pvalue <0.05)

cluster	gene	avg_log2FC	p_val_adj	cluster	gene	avg_log2FC	p_val_adj	cluster	gene	avg_log2FC	p_val_adj
ZG1	Agmat	1.46	1E-57	ZG2	Synpo2	1.72	2E-68	ZG3	1700016P03Rik	4.96	4E-168
ZG1	Eno3	1.40	2E-197	ZG2	Gm57425	1.49	3E-21	ZG3	Gm7247	4.09	1E-66
ZG1	Gulo	1.32	2E-62	ZG2	C030034L19Rik	1.43	1E-49	ZG3	Gm3294	3.85	1E-64
ZG1	Gm29157	1.24	8E-97	ZG2	Osbpl10	1.41	1E-17	ZG3	Nr4a2	3.78	7E-231
ZG1	Cd24a	1.21	7E-45	ZG2	6330411D24Rik	1.38	4E-44	ZG3	Notch3	3.70	3E-77
ZG1	Ecrg4	1.20	0E+00	ZG2	Ano3	1.35	3E-04	ZG3	Gm17276	3.57	4E-179
ZG1	Tmem121	1.16	6E-74	ZG2	Trabd2b	1.35	1E-30	ZG3	Sik1	3.38	1E-226
ZG1	Scg5	1.10	5E-77	ZG2	Gm48094	1.35	4E-31	ZG3	Nr4a1	3.38	1E-219
ZG1	Flt4	1.09	6E-11	ZG2	Tmem132c	1.32	1E-28	ZG3	Acvr1c	3.29	2E-219
ZG1	Cyp11b2	1.06	6E-142	ZG2	Cadps	1.27	2E-21	ZG3	Tafa2	3.14	5E-46
ZG1	Prss12	1.02	7E-22	ZG2	Lama3	1.25	2E-70	ZG3	Pde10a	3.09	2E-209
ZG1	Fdps	1.01	1E-84	ZG2	Gm28905	1.24	6E-16	ZG3	Arid5a	3.07	3E-67
ZG1	Slc2a4	1.01	3E-36	ZG2	Fat3	1.21	4E-127	ZG3	Gm56730	3.04	5E-80
ZG1	Steap1	0.99	2E-225	ZG2	Gm14344	1.19	7E-37	ZG3	Slc6a5	3.03	2E-189
ZG1	Sqle	0.96	8E-18	ZG2	Cap2	1.18	6E-50	ZG3	Nfil3	2.97	6E-196
ZG1	Idi1	0.94	2E-62	ZG2	Gm16064	1.17	1E-14	ZG3	Tmem266	2.92	6E-80
ZG1	Pnma8a	0.93	5E-23	ZG2	Prr16	1.17	1E-26	ZG3	Gm13889	2.74	6E-103
ZG1	Rab33a	0.92	2E-96	ZG2	Gm26652	1.15	6E-09	ZG3	Hmgcr	2.69	6E-129
ZG1	Tmem158	0.91	3E-56	ZG2	Opcml	1.15	4E-05	ZG3	Pex5l	2.69	4E-52
ZG1	Fabp5	0.90	7E-59	ZG2	9630014M24Rik	1.15	4E-27	ZG3	Ark2c	2.69	7E-68
ZG1	Col18a1	0.90	5E-94	ZG2	Gm30624	1.12	9E-14	ZG3	Kcnk5	2.67	3E-154
ZG1	Cpb1	0.90	3E-144	ZG2	Nrp1	1.12	3E-30	ZG3	Adamts1	2.61	1E-28
ZG1	Cldn10	0.90	7E-21	ZG2	Cyp7b1	1.09	4E-10	ZG3	Nrg4	2.56	5E-33
ZG1	Fdx1	0.88	3E-208	ZG2	Gm49890	1.09	8E-22	ZG3	Batf3	2.53	4E-82
ZG1	Emp2	0.88	8E-09	ZG2	Pdzd2	1.09	8E-25	ZG3	Ldlr	2.52	6E-141
ZG1	Ppp1r14c	0.87	4E-17	ZG2	Insyn2a	1.08	2E-20	ZG3	Adamts9	2.51	8E-72
ZG1	Clstn3	0.86	8E-81	ZG2	Gm26944	1.08	8E-23	ZG3	Gm12059	2.49	1E-29
ZG1	Tubb2a	0.84	2E-40	ZG2	Kif6	1.07	4E-16	ZG3	Klf4	2.43	1E-76
ZG1	Traf4	0.84	4E-29	ZG2	Amph	1.07	3E-51	ZG3	Myot	2.40	1E-69
ZG1	Epn3	0.84	4E-13	ZG2	Grin2a	1.05	3E-06	ZG3	Proser2	2.39	5E-52
ZG1	Ctnnd2	0.84	2E-42	ZG2	Gm15594	1.04	1E-21	ZG3	Bmp2	2.34	2E-54
ZG1	Cyp51	0.83	3E-50	ZG2	Fam124a	1.04	5E-19	ZG3	Gm2897	2.30	3E-43
ZG1	Ntrk3	0.82	5E-27	ZG2	Sox6	1.03	1E-85	ZG3	Gm16062	2.27	4E-106
ZG1	Hpcal4	0.82	8E-14	ZG2	Dixdc1	1.03	2E-19	ZG3	Coq10b	2.22	3E-138
ZG1	Jaml	0.82	1E-24	ZG2	Gm42439	1.03	6E-46	ZG3	Plk3	2.22	2E-28
ZG1	Fads2	0.81	2E-119	ZG2	Gm15764	1.02	6E-21	ZG3	Ntrk2	2.19	1E-22
ZG1	Camk2n2	0.81	3E-37	ZG2	9630002D21Rik	1.02	7E-114	ZG3	Camk2n2	2.18	9E-125
ZG1	Pcdh19	0.81	8E-101	ZG2	Nrep	1.02	2E-08	ZG3	Thsd7a	2.18	1E-36
ZG1	Egfl6	0.80	4E-40	ZG2	Zdhhc14	1.02	6E-36	ZG3	Gm57354	2.17	1E-80
ZG1	Tafa4	0.79	1E-27	ZG2	Kcnd2	1.01	4E-136	ZG3	Usp2	2.14	6E-109
ZG1	Mvd	0.77	4E-04	ZG2	Kcng3	1.01	2E-60	ZG3	Gm21973	2.11	7E-29
ZG1	Htr3a	0.77	4E-41	ZG2	Gm14066	1.01	3E-44	ZG3	Gad1	2.08	3E-47
ZG1	Bex1	0.76	1E-34	ZG2	Gm32250	1.00	4E-17	ZG3	Ctnnd2	2.07	3E-45
ZG1	Msmo1	0.76	1E-91	ZG2	Tmtc1	1.00	7E-29	ZG3	Bean1	2.07	2E-38
ZG1	Procr	0.75	5E-43	ZG2	Gm32304	1.00	1E-40	ZG3	Irs2	2.06	3E-79
ZG1	Icam4	0.73	1E-11	ZG2	Clic6	0.99	3E-25	ZG3	Agxt2	2.04	3E-11
ZG1	Gm56664	0.73	8E-15	ZG2	Cacna2d3	0.99	7E-104	ZG3	Adarb1	2.04	2E-87
ZG1	Txn2	0.73	2E-232	ZG2	Kcnj12	0.99	5E-13	ZG3	Procr	2.03	5E-89
ZG1	Fam81a	0.73	9E-20	ZG2	Pgbd1	0.99	7E-13	ZG3	Noct	2.01	2E-76
ZG1	Ncald	0.72	8E-79	ZG2	Samd5	0.99	3E-32	ZG3	Adamts20	2.00	1E-17
ZG1	Neurl1a	0.70	2E-09	ZG2	Gm10848	0.99	4E-73	ZG3	Crem	2.00	2E-155
ZG1	Tspan6	0.70	2E-79	ZG2	Egflam	0.98	7E-11	ZG3	6030498E09Rik	1.99	1E-13
ZG1	Fgf20	0.70	2E-12	ZG2	Tafa4	0.98	4E-28	ZG3	Fhl2	1.98	3E-10
ZG1	Dhcr7	0.69	2E-72	ZG2	Hs3st1	0.97	3E-16	ZG3	Mir670hg	1.98	4E-14
ZG1	Syne4	0.69	3E-23	ZG2	Frmpd4	0.96	2E-177	ZG3	Shroom4	1.97	1E-94
ZG1	Car8	0.69	1E-03	ZG2	Astn2	0.96	2E-18	ZG3	Cyp11b2	1.94	1E-78
ZG1	Apoc4	0.69	4E-98	ZG2	Pard3bos1	0.95	2E-08	ZG3	Dusp5	1.93	7E-32
ZG1	Vsnl1	0.69	2E-180	ZG2	Sptlc3	0.95	6E-15	ZG3	Pde4d	1.91	2E-43
ZG1	Igfbp3	0.68	2E-07	ZG2	Ppargc1a	0.95	3E-23	ZG3	Fosb	1.91	2E-94
ZG1	Dkk3	0.68	8E-12	ZG2	St6galnac3	0.94	4E-15	ZG3	S1pr3	1.90	8E-40
ZG1	Tm7sf2	0.68	2E-03	ZG2	4930578G10Rik	0.93	7E-14	ZG3	Fosl2	1.90	4E-91
ZG1	Rtl8a	0.67	9E-99	ZG2	Gm17036	0.93	5E-09	ZG3	Ptgs2	1.90	4E-18
ZG1	Rasl10b	0.66	6E-21	ZG2	Fhod3	0.92	2E-29	ZG3	Tent5a	1.89	1E-72
ZG1	Cspg5	0.66	7E-68	ZG2	Gm3289	0.92	6E-46	ZG3	Nebl	1.89	1E-11
ZG1	Socs1	0.66	5E-28	ZG2	Unc79	0.92	8E-13	ZG3	Ntrk3	1.89	3E-47
ZG1	Sp5	0.66	3E-17	ZG2	Prkaa2	0.92	6E-32	ZG3	Pvr	1.84	2E-43
ZG1	Hmgn3	0.66	4E-106	ZG2	Daam2	0.91	2E-106	ZG3	Fdx1	1.84	7E-182
ZG1	Pmvk	0.66	2E-12	ZG2	Lhfpl2	0.91	3E-106	ZG3	Cpa3	1.82	5E-28
ZG1	Fbln1	0.66	1E-55	ZG2	Klf12	0.91	3E-166	ZG3	Lrrc73	1.79	7E-35
ZG1	Lrrc55	0.65	3E-81	ZG2	D7Ertd443e	0.90	3E-49	ZG3	Osbp2	1.78	6E-26
ZG1	Sc5d	0.65	2E-36	ZG2	Gm13269	0.90	2E-55	ZG3	Ncald	1.78	5E-98
ZG1	Grep1	0.65	1E-11	ZG2	Gm20404	0.90	6E-06	ZG3	Csrnp1	1.77	1E-23
ZG1	Triobp	0.65	3E-83	ZG2	Flvcr2	0.90	1E-25	ZG3	Unc5c	1.77	5E-21
ZG1	Hmgb3	0.64	6E-183	ZG2	Nr3c2	0.89	2E-52	ZG3	Baalc	1.77	3E-32
ZG1	Myot	0.64	2E-07	ZG2	Sorbs2os	0.89	1E-66	ZG3	Dusp8	1.76	3E-54
ZG1	Car2	0.63	4E-104	ZG2	Gm3667	0.89	8E-11	ZG3	Gm16153	1.76	3E-14
ZG1	Gamt	0.63	6E-145	ZG2	Mid1	0.89	7E-93	ZG3	Cpeb4	1.75	3E-104
ZG1	Rundc3a	0.62	3E-23	ZG2	Gm26672	0.89	4E-06	ZG3	Flcn	1.75	8E-56
ZG1	Ntm	0.62	7E-12	ZG2	Trib2	0.89	4E-31	ZG3	Kcnq3	1.73	9E-49
ZG1	Agtr2	0.62	2E-02	ZG2	Lzts1	0.89	4E-22	ZG3	Per1	1.73	2E-60
ZG1	Nudt10	0.62	2E-07	ZG2	Gm4876	0.89	2E-23	ZG3	P3h2	1.71	9E-45
ZG1	eGFP	0.62	2E-209	ZG2	Arhgap20	0.88	1E-06	ZG3	Man1a	1.70	3E-92
ZG1	Impdh1	0.61	2E-12	ZG2	Pde1a	0.88	1E-02	ZG3	Slc2a13	1.69	1E-31
ZG1	Lipa	0.61	2E-50	ZG2	Gm20422	0.88	6E-08	ZG3	Cbarp	1.69	2E-67
ZG1	Tmem100	0.61	3E-04	ZG2	Gm39094	0.88	1E-07	ZG3	Cbx4	1.68	5E-121
ZG1	Rtl8b	0.60	6E-62	ZG2	Igdcc3	0.88	2E-16	ZG3	Gm56880	1.67	2E-13
ZG1	Fdft1	0.60	1E-24	ZG2	A330070K13Rik	0.88	5E-15	ZG3	Srxn1	1.66	2E-53
ZG1	Hsd17b7	0.60	2E-07	ZG2	Cacnb2	0.88	3E-168	ZG3	Mpp7	1.66	3E-10
ZG1	Lrrc8e	0.60	6E-18	ZG2	Gm14342	0.88	1E-19	ZG3	Pld1	1.66	2E-11
ZG1	Gm30085	0.60	7E-08	ZG2	Eda2r	0.88	6E-06	ZG3	Kif26b	1.65	1E-15
ZG1	Tceal3	0.60	6E-59	ZG2	Npas3	0.87	2E-101	ZG3	Cyp51	1.63	1E-59
ZG1	Rwdd2a	0.59	8E-12	ZG2	D130043K22Rik	0.87	3E-12	ZG3	Ypel4	1.62	1E-18
ZG1	Batf3	0.59	4E-11	ZG2	Arhgap6	0.87	3E-09	ZG3	Prss12	1.62	3E-27
ZG1	Usp35	0.59	3E-08	ZG2	Gm42567	0.87	5E-16	ZG3	Pard6a	1.60	2E-57
ZG1	Tub	0.59	1E-46	ZG2	Rttn	0.86	2E-12	ZG3	Nr4a3	1.60	7E-101
ZG1	Sh3bp4	0.58	1E-38	ZG2	B130024G19Rik	0.86	3E-26	ZG3	Ppp2r2b	1.60	2E-54
ZG1	Ctsf	0.58	4E-133	ZG2	Sntb1	0.86	4E-100	ZG3	Tmem178	1.59	1E-64
ZG1	Fam43a	0.58	7E-22	ZG2	Gm49355	0.85	9E-08	ZG3	Gm56555	1.58	7E-35
ZG1	Dusp14	0.58	2E-08	ZG2	Nfat5	0.85	3E-154	ZG3	ENSMUSG000001	1.57	3E-12
ZG1	Itih5	0.57	3E-14	ZG2	Nox1	0.85	2E-03	ZG3	Trp53inp1	1.57	8E-76

Supplementary Figure 1: (**a**) List of the top 100 marker genes for each cell clusters identified in Figure 1C. Complete list available in Supplementary Table 7. (**b**) UMAP representation of mki67 expression, proliferative marker. (**c**) UMAP representation of *Abcb1b*, *Mgst2*, *Sbsn,* and *Srd5a2* expression, markers of inner zF cell population. (**d**) UMAP representation of zG enriched genes *Ppp2r2b* and *Pcdh19*. (**e**) UMAP representation of inner zF enriched genes: *Mmd2*, *Acsbg1* and *Abca1*. (**f**) Expression of HHEX (in brown) and DAB2 (purple) in 15-week-old *WT* and *SF1-Cre Hhex KO* male adrenals by immunohistochemistry (IHC). Nuclei were stained in blue with hematoxylin. Cap.: capsule, zG: zona glomerulosa, zF: zona fasciculata, Med.: medulla (**g**) Representation of HHEX expression in various human tissues (GTEx dataset).

Dumontet et al. 2025: Supplementary Figure 1

1a

Top 100 gene marker genes – Page 2/2 – Complete list on Supplementary Table 7

zG/zF population Ranked by avg_Log2FC (adjusted pvalue <0.05)

cluster	gene	avg_log2FC	p_val_adj
ZG/ZF	Malat1	1.75	9E-13
ZG/ZF	Firre	1.53	4E-04
ZG/ZF	Dync2h1	1.19	2E-04
ZG/ZF	Rfx7	1.00	3E-04
ZG/ZF	Zfp407	0.99	1E-03
ZG/ZF	Kazn	0.97	7E-03
ZG/ZF	Pard3b	0.97	2E-03
ZG/ZF	Pnisr	0.93	1E-05
ZG/ZF	E430024I08Rik	0.92	5E-04
ZG/ZF	Vps13a	0.88	2E-02
ZG/ZF	Ralgapa1	0.88	1E-03
ZG/ZF	Wdpcp	0.86	1E-02
ZG/ZF	Zcchc7	0.86	5E-03
ZG/ZF	Lcorl	0.84	2E-03
ZG/ZF	Nfat5	0.83	3E-05
ZG/ZF	Ppp1r9a	0.83	4E-03
ZG/ZF	Auts2	0.83	1E-05
ZG/ZF	Agap1	0.83	8E-03
ZG/ZF	Atad2b	0.83	3E-02
ZG/ZF	Dmd	0.82	7E-03
ZG/ZF	Cacnb2	0.81	6E-04
ZG/ZF	Kcnq1ot1	0.80	8E-03
ZG/ZF	Cacna1c	0.79	6E-05
ZG/ZF	Trio	0.79	8E-03
ZG/ZF	Phlpp1	0.77	1E-02
ZG/ZF	Psd3	0.77	6E-03
ZG/ZF	Dlgap1	0.77	4E-04
ZG/ZF	Plcb1	0.75	4E-03
ZG/ZF	Utrn	0.75	4E-04
ZG/ZF	Btrc	0.74	1E-02
ZG/ZF	Gramd1b	0.74	1E-02
ZG/ZF	Nckap5	0.73	3E-03
ZG/ZF	Zbtb20	0.70	5E-04
ZG/ZF	Mast4	0.67	4E-02
ZG/ZF	Rapgef4	0.64	2E-02
ZG/ZF	Kcnma1	0.63	2E-02
ZG/ZF	Rora	0.54	2E-03
ZG/ZF	Shroom4	0.47	3E-02

zF populations Ranked by avg_Log2FC (adjusted pvalue <0.05)

cluster	gene	avg_log2FC	p_val_adj
ZF	Mmd2	4.26	1E-266
ZF	Aff2	3.61	1E-75
ZF	Acsbg1	3.50	7E-234
ZF	Sema5b	2.88	5E-88
ZF	Cyp11b1	2.86	6E-266
ZF	F3	2.81	2E-62
ZF	Fabp3	2.71	1E-76
ZF	Drd4	2.68	4E-53
ZF	Gm31333	2.68	5E-67
ZF	Col5a1	2.67	2E-68
ZF	Vegfd	2.55	1E-53
ZF	Cited1	2.50	3E-159
ZF	Tmem52	2.40	4E-174
ZF	Tspan15	2.39	1E-48
ZF	Csdc2	2.39	4E-106
ZF	Gml2	2.36	6E-64
ZF	Cacnb4	2.30	5E-56
ZF	Hhex	2.22	1E-139
ZF	Vnn1	2.16	9E-41
ZF	Plin5	2.14	6E-41
ZF	Adgrg1	2.09	7E-37
ZF	Gdf15	2.08	7E-04
ZF	Cryab	2.05	4E-59
ZF	Scube3	1.99	2E-87
ZF	Rapsn	1.91	1E-52
ZF	Mt2	1.87	6E-72
ZF	Scx	1.87	1E-156
ZF	Dbn1	1.86	1E-66
ZF	Gm20629	1.83	1E-38
ZF	Adh1	1.78	2E-187
ZF	Gstm7	1.77	2E-96
ZF	C4b	1.73	6E-119
ZF	Wfdc6a	1.70	6E-26
ZF	Acan	1.69	1E-121
ZF	Klf15	1.69	1E-34
ZF	Sh2d4a	1.66	5E-53
ZF	Epas1	1.65	5E-104
ZF	Prss35	1.63	5E-211
ZF	C4a	1.59	9E-107
ZF	Id1	1.57	4E-39
ZF	Abca1	1.57	6E-155
ZF	Serpinb6b	1.56	2E-109
ZF	Frmd5	1.55	7E-53
ZF	Rnd3	1.54	1E-80
ZF	Myof	1.53	2E-38
ZF	Mapt	1.52	1E-69
ZF	Adamts6	1.51	3E-45
ZF	Selenbp1	1.50	7E-114
ZF	Adhfe1	1.50	3E-51
ZF	Hmox1	1.48	3E-41
ZF	Col6a1	1.48	1E-123
ZF	Nr1h4	1.47	5E-41
ZF	Gstt1	1.44	4E-135
ZF	Kdr	1.43	5E-20
ZF	Aldh1l1	1.41	3E-68
ZF	Wnt5b	1.40	4E-17
ZF	Gm14858	1.39	7E-20
ZF	Gm15958	1.38	7E-29
ZF	Plxdc2	1.36	5E-31
ZF	Susd3	1.36	3E-89
ZF	Slc37a2	1.35	4E-27
ZF	Itga1	1.34	9E-57
ZF	Igf1	1.34	4E-31
ZF	Isg15	1.33	3E-07
ZF	Gm13483	1.28	4E-24
ZF	Gm15201	1.28	2E-21
ZF	D330050G23Rik	1.28	2E-31
ZF	1700087I21Rik	1.27	3E-16
ZF	Ampd3	1.27	1E-31
ZF	Tmem117	1.26	2E-28
ZF	Timp2	1.26	4E-71
ZF	Cdkn1c	1.25	6E-149
ZF	Dpf3	1.25	5E-12
ZF	Pdlim2	1.25	4E-64
ZF	Amotl2	1.25	1E-54
ZF	Fads6	1.24	1E-62
ZF	Col4a5	1.24	8E-30
ZF	Sez6l2	1.23	2E-48
ZF	Gadd45a	1.23	2E-27
ZF	Spry1	1.23	8E-53
ZF	Mt1	1.22	1E-103
ZF	Anxa5	1.19	1E-268
ZF	Nupr1	1.19	4E-08
ZF	Nt5e	1.19	8E-11
ZF	Serpinb9	1.19	7E-76
ZF	Akr1cl	1.19	0E+00
ZF	Abca5	1.18	7E-22
ZF	Wnt5a	1.18	1E-50
ZF	Apln	1.15	1E-07
ZF	Fosl2	1.14	3E-89
ZF	Nat8f1	1.14	4E-59
ZF	Rbms3	1.14	4E-84
ZF	F11r	1.13	4E-70
ZF	Prss36	1.13	6E-14
ZF	Naaladl2	1.13	3E-15
ZF	Mill2	1.12	3E-30
ZF	Srd5a2	1.12	1E-87
ZF	Gm12216	1.12	5E-18
ZF	Gstm1	1.12	4E-243
ZF	Pakap	1.11	1E-19

cluster	gene	avg_log2FC	p_val_adj
Inner.ZF	D630023F18Rik	10.50	0E+00
Inner.ZF	Ahnak2	10.26	0E+00
Inner.ZF	Shc2	10.03	1E-187
Inner.ZF	Gm45338	10.03	1E-187
Inner.ZF	Adgra1	9.91	1E-155
Inner.ZF	Aldh1a1	9.23	0E+00
Inner.ZF	Masp1	9.14	0E+00
Inner.ZF	Kcnj4	9.03	1E-155
Inner.ZF	Gpx3	8.78	1E-177
Inner.ZF	Slc6a17	8.77	8E-133
Inner.ZF	Ackr3	8.72	0E+00
Inner.ZF	Mgst2	8.67	0E+00
Inner.ZF	Cabp1	8.63	0E+00
Inner.ZF	Acsbg2	8.45	4E-183
Inner.ZF	Gm13031	8.45	6E-119
Inner.ZF	Adgrg2	8.18	4E-183
Inner.ZF	S100a16	8.11	0E+00
Inner.ZF	Ptprt	8.03	1E-158
Inner.ZF	Apol6	7.91	1E-188
Inner.ZF	Sbsn	7.90	6E-124
Inner.ZF	Atp2b2	7.86	8E-84
Inner.ZF	Wnt10b	7.71	8E-277
Inner.ZF	Gabrb1	7.61	7E-45
Inner.ZF	Cntn3	7.55	6E-241
Inner.ZF	Srd5a2	7.49	2E-128
Inner.ZF	Sytl5	7.44	0E+00
Inner.ZF	Aif1l	7.35	0E+00
Inner.ZF	Mapk13	7.32	6E-274
Inner.ZF	Runx2	7.31	2E-186
Inner.ZF	Abcb1b	7.26	2E-25
Inner.ZF	Flnc	7.09	3E-47
Inner.ZF	Palm3	7.03	2E-112
Inner.ZF	Runx2os1	7.00	0E+00
Inner.ZF	Adamts15	6.98	3E-68
Inner.ZF	Cgref1	6.96	2E-117
Inner.ZF	Fam20a	6.93	4E-214
Inner.ZF	Pde1b	6.92	6E-281
Inner.ZF	Aldh1a7	6.91	2E-251
Inner.ZF	Plin4	6.89	1E-171
Inner.ZF	Gm16178	6.80	0E+00
Inner.ZF	Ildr2	6.54	5E-158
Inner.ZF	Crispld2	6.45	4E-137
Inner.ZF	Mrgpre	6.45	1E-105
Inner.ZF	Meiob	6.33	2E-110
Inner.ZF	Txndc2	6.31	2E-82
Inner.ZF	Abcb1a	6.19	1E-209
Inner.ZF	Htra3	6.17	2E-159
Inner.ZF	Radil	6.08	1E-103
Inner.ZF	4833422C13Rik	6.05	2E-119
Inner.ZF	Matn2	6.05	8E-153
Inner.ZF	Tgfbi	5.96	1E-130
Inner.ZF	Tent5b	5.95	1E-117
Inner.ZF	Kcnd3os	5.95	4E-30
Inner.ZF	Il34	5.92	3E-131
Inner.ZF	Prune2	5.92	4E-128
Inner.ZF	Jakmip3	5.88	5E-92
Inner.ZF	Rhov	5.82	6E-63
Inner.ZF	Acat3	5.82	5E-61
Inner.ZF	Rgcc	5.81	4E-92
Inner.ZF	Rpp25	5.78	1E-57
Inner.ZF	Nkain4	5.78	7E-24
Inner.ZF	Apol9a	5.78	4E-142
Inner.ZF	Layn	5.66	3E-141
Inner.ZF	1810064F22Rik	5.65	9E-73
Inner.ZF	Ppl	5.64	1E-105
Inner.ZF	Hspb8	5.59	3E-171
Inner.ZF	Myo1a	5.57	2E-46
Inner.ZF	Col5a3	5.54	6E-161
Inner.ZF	Anpep	5.49	1E-55
Inner.ZF	Osmr	5.48	5E-72
Inner.ZF	Stard13	5.45	3E-41
Inner.ZF	Nell2	5.45	7E-36
Inner.ZF	Gm2350	5.35	1E-27
Inner.ZF	Mmp19	5.30	4E-130
Inner.ZF	Slc25a30	5.26	3E-53
Inner.ZF	P2rx6	5.24	3E-45
Inner.ZF	Cited4	5.19	3E-91
Inner.ZF	Thpo	5.18	2E-21
Inner.ZF	Erbb3	5.18	2E-20
Inner.ZF	Rnf208	5.18	2E-19
Inner.ZF	Lgals3	5.18	1E-218
Inner.ZF	Rab11fip4	5.17	2E-51
Inner.ZF	Scara5	5.17	2E-78
Inner.ZF	Cp	5.16	1E-19
Inner.ZF	Tnfrsf1b	5.14	6E-90
Inner.ZF	Frmd5	5.12	3E-53
Inner.ZF	Tnxb	5.05	5E-85
Inner.ZF	Stx11	5.03	6E-134
Inner.ZF	Apol9b	5.01	1E-63
Inner.ZF	Rarb	4.94	4E-23
Inner.ZF	D430041D05Rik	4.93	5E-76
Inner.ZF	Sh3tc1	4.93	2E-52
Inner.ZF	Efnb2	4.91	2E-10
Inner.ZF	Gask1b	4.88	3E-61
Inner.ZF	Hmga2-ps1	4.88	7E-73
Inner.ZF	Ptprn	4.84	2E-38
Inner.ZF	Fgf7	4.82	1E-22
Inner.ZF	Dio3os	4.81	3E-27
Inner.ZF	Mcub	4.81	2E-133
Inner.ZF	Pcsk6	4.80	1E-26

Non steroidogenic Ranked by avg_Log2FC (adjusted pvalue <0.05)

cluster	gene	avg_log2FC	p_val_adj
on-steroidoger	Ccr2	14.64	0E+00
on-steroidoger	Trbc2	14.14	1E-193
on-steroidoger	Il1b	13.91	0E+00
on-steroidoger	Grap2	13.85	0E+00
on-steroidoger	Ly6c2	13.82	1E-264
on-steroidoger	Klrk1	13.73	1E-201
on-steroidoger	Ifi209	13.44	0E+00
on-steroidoger	Cd79b	13.35	0E+00
on-steroidoger	Slfn1	13.25	0E+00
on-steroidoger	F630028O10Rik	13.22	0E+00
on-steroidoger	Cd2	13.19	0E+00
on-steroidoger	Napsa	13.15	0E+00
on-steroidoger	2310001H17Rik	13.14	0E+00
on-steroidoger	Emilin2	13.06	0E+00
on-steroidoger	S100a8	12.99	3E-125
on-steroidoger	Il18rap	12.97	2E-280
on-steroidoger	Gm56663	12.88	4E-296
on-steroidoger	Cd53	12.88	0E+00
on-steroidoger	Cd3d	12.85	1E-193
on-steroidoger	Mcemp1	12.80	1E-264
on-steroidoger	Cmah	12.79	0E+00
on-steroidoger	Lilrb4a	12.66	0E+00
on-steroidoger	S100a9	12.65	8E-93
on-steroidoger	Cd7	12.62	2E-154
on-steroidoger	Sash3	12.55	0E+00
on-steroidoger	Ccl3	12.53	5E-233
on-steroidoger	Gimap3	12.50	0E+00
on-steroidoger	Csf2rb	12.50	0E+00
on-steroidoger	Cd300lb	12.48	2E-272
on-steroidoger	Iglc2	12.44	2E-281
on-steroidoger	Cd101	12.44	5E-233
on-steroidoger	Prkcq	12.43	6E-241
on-steroidoger	Abcg3	12.42	0E+00
on-steroidoger	Dhrs9	12.40	1E-138
on-steroidoger	Anxa1	12.36	0E+00
on-steroidoger	Tifab	12.36	0E+00
on-steroidoger	Ccr7	12.35	8E-249
on-steroidoger	Mirt1	12.33	3E-225
on-steroidoger	E330020D12Rik	12.32	3E-288
on-steroidoger	Rhoh	12.26	0E+00
on-steroidoger	Clec12a	12.26	0E+00
on-steroidoger	Ptafr	12.25	2E-280
on-steroidoger	Ccr1	12.23	1E-256
on-steroidoger	Trbc1	12.20	5E-178
on-steroidoger	Cd300ld	12.20	3E-225
on-steroidoger	Plac8	12.18	0E+00
on-steroidoger	Clec4a3	12.17	0E+00
on-steroidoger	Ifi206	12.13	2E-280
on-steroidoger	S1pr4	12.11	2E-280
on-steroidoger	Mir142hg	12.10	0E+00
on-steroidoger	Lax1	12.10	1E-264
on-steroidoger	Cybb	12.06	0E+00
on-steroidoger	Pld4	12.06	0E+00
on-steroidoger	AW112010	12.05	0E+00
on-steroidoger	Samd3	12.02	3E-162
on-steroidoger	Fgd2	11.99	0E+00
on-steroidoger	Rac2	11.99	0E+00
on-steroidoger	Selplg	11.98	0E+00
on-steroidoger	Gm35154	11.97	8E-249
on-steroidoger	Rnase6	11.95	0E+00
on-steroidoger	Pou2af1	11.93	8E-186
on-steroidoger	Tnfrsf13c	11.92	2E-209
on-steroidoger	Tox	11.92	3E-217
on-steroidoger	Klrb1c	11.89	1E-146
on-steroidoger	Siglecg	11.88	3E-280
on-steroidoger	Lair1	11.86	0E+00
on-steroidoger	Vis1	11.82	3E-217
on-steroidoger	Fcmr	11.82	3E-280
on-steroidoger	Ifitm6	11.82	8E-186
on-steroidoger	Ccl4	11.81	3E-280
on-steroidoger	Arhgap45	11.81	0E+00
on-steroidoger	Mmp8	11.80	3E-162
on-steroidoger	Rgs14	11.80	2E-280
on-steroidoger	Mmp9	11.79	2E-139
on-steroidoger	Treml2	11.78	3E-217
on-steroidoger	Ccl9	11.74	3E-288
on-steroidoger	Siglece	11.72	5E-233
on-steroidoger	Acp5	11.72	0E+00
on-steroidoger	Lilrb4b	11.71	0E+00
on-steroidoger	Gimap7	11.69	3E-217
on-steroidoger	Gpr174	11.67	5E-233
on-steroidoger	Sirpb1c	11.67	0E+00
on-steroidoger	Nkg7	11.67	4E-225
on-steroidoger	Tlr13	11.66	3E-217
on-steroidoger	Ipcef1	11.64	0E+00
on-steroidoger	Ms4a4b	11.63	0E+00
on-steroidoger	Igkc	11.63	8E-281
on-steroidoger	Gvin2	11.63	8E-249
on-steroidoger	Clec4e	11.61	1E-138
on-steroidoger	Cd28	11.61	2E-146
on-steroidoger	Wfdc21	11.60	1E-146
on-steroidoger	Btla	11.60	0E+00
on-steroidoger	Ptpro	11.58	0E+00
on-steroidoger	Ikzf1	11.58	0E+00
on-steroidoger	P2ry10	11.58	6E-178
on-steroidoger	5830428M24Rik	11.58	1E-201
on-steroidoger	Sirpb1a	11.58	3E-225
on-steroidoger	Cd33	11.54	2E-264
on-steroidoger	Itgal	11.52	0E+00
on-steroidoger	Inpp5d	11.44	0E+00

Supplementary Figure 1: (**a**) List of the top 100 marker genes for each cell clusters identified in Figure 1C. Complete list available in Supplementary Table 7. (**b**) UMAP representation of mki67 expression, proliferative marker. (**c**) UMAP representation of *Abcb1b*, *Mgst2*, *Sbsn,* and *Srd5a2* expression, markers of inner zF cell population. (**d**) UMAP representation of zG enriched genes *Ppp2r2b* and *Pcdh19*. (**e**) UMAP representation of inner zF enriched genes: *Mmd2*, *Acsbg1* and *Abca1*. (**f**) Expression of HHEX (in brown) and DAB2 (purple) in 15-week-old *WT* and *SF1-Cre Hhex KO* male adrenals by immunohistochemistry (IHC). Nuclei were stained in blue with hematoxylin. Cap.: capsule, zG: zona glomerulosa, zF: zona fasciculata, Med.: medulla (**g**) Representation of HHEX expression in various human tissues (GTEx dataset).

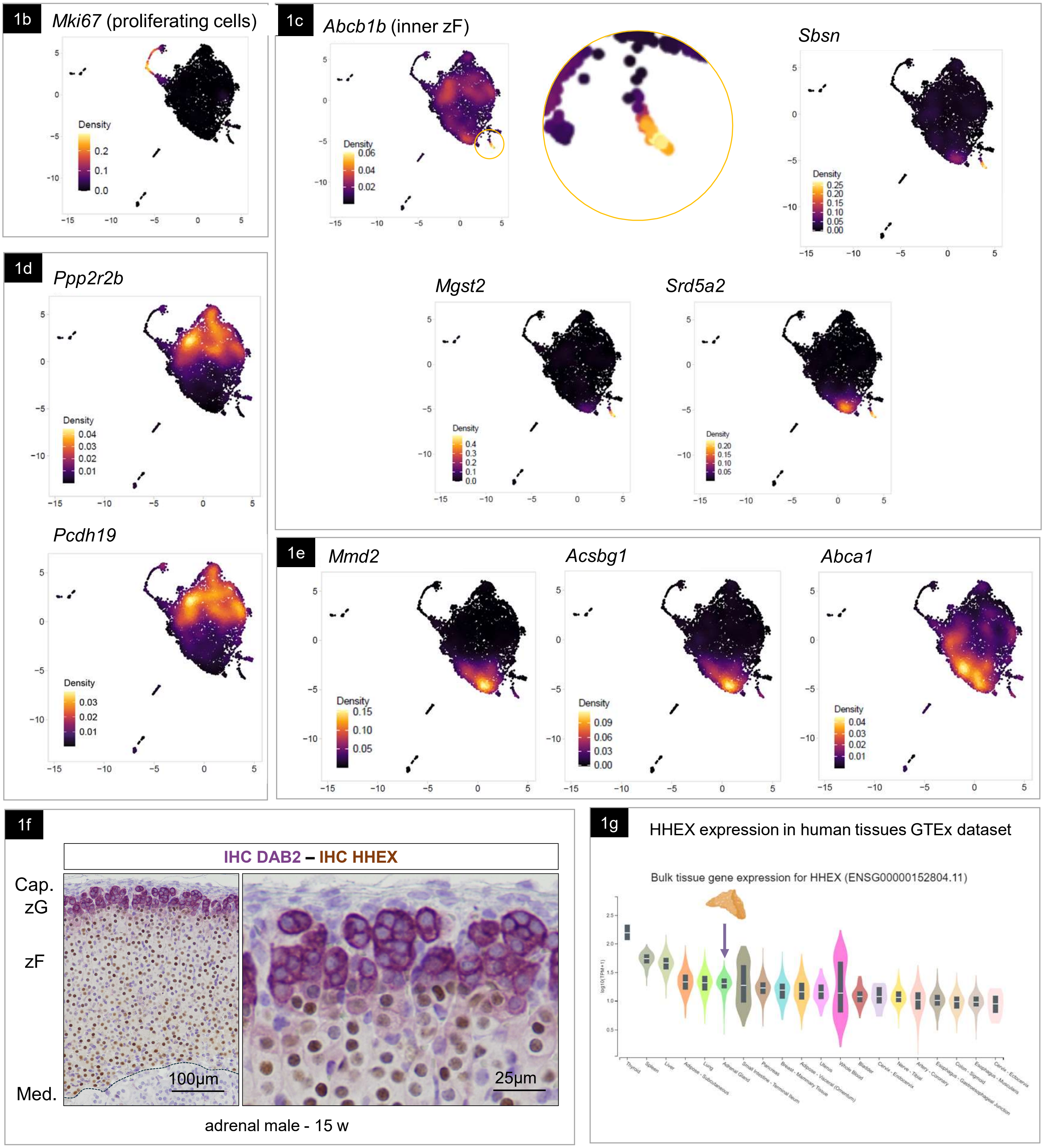

Supplementary Figure 1: (**a**) List of the top 100 marker genes for each cell clusters identified in Figure 1C. Complete list available in Supplementary Table 7. (**b**) UMAP representation of mki67 expression, proliferative marker. (**c**) UMAP representation of *Abcb1b*, *Mgst2*, *Sbsn,* and *Srd5a2* expression, markers of inner zF cell population. (**d**) UMAP representation of zG enriched genes *Ppp2r2b* and *Pcdh19*. (**e**) UMAP representation of inner zF enriched genes: *Mmd2*, *Acsbg1* and *Abca1*. (**f**) Expression of HHEX (in brown) and DAB2 (purple) in 15-week-old *WT* and *SF1-Cre Hhex KO* male adrenals by immunohistochemistry (IHC). Nuclei were stained in blue with hematoxylin. Cap.: capsule, zG: zona glomerulosa, zF: zona fasciculata, Med.: medulla (**g**) Representation of HHEX expression in various human tissues (GTEx dataset).

Dumontet et al. 2025: Supplementary Figure 2

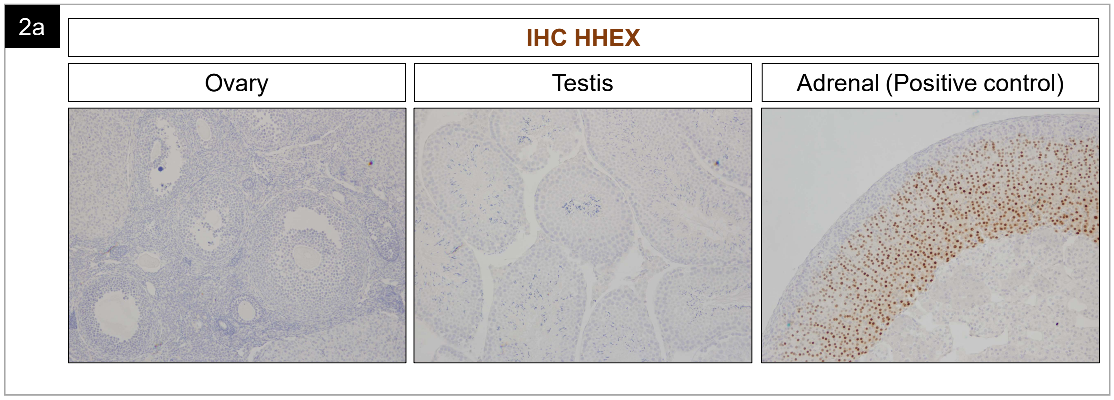

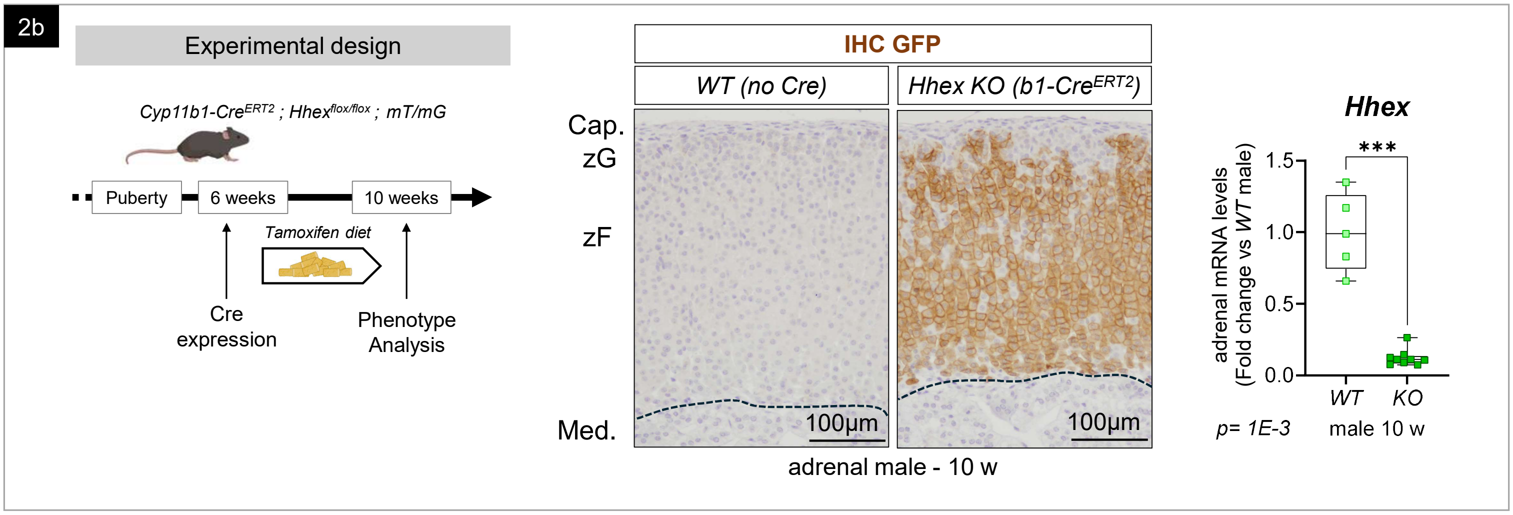

Supplementary Figure 2: (**a**) Absence of HHEX expression in mouse testis and ovaries assessed by immunohistochemistry. A section from an adult male adrenal was used as positive control (**b**) (A) Schematic representation of the transgenic mouse models used to inactivate HHEX conditionally in the zona fasciculata using *Cyp11b1-Cre*^{ERT2}-mediated recombination of exon 2 and 3 of *Hhex* gene. GFP immunohistochemistry (IHC) demonstrating the efficiency of the recombination in the zona fasciculata in *Cyp11b1-CreERT2 ; Hhex KO* mice. Quantification of *Hhex* transcripts by RT-qPCR in 10-week-old *WT* (n=5) and *Cyp11b1-Cre*^{ERT2} *Hhex KO* (n=9) male adrenals. Graph represents box plots with individual biological replicates. *p*-value (*p*) was calculated using a Mann-Whitney test.

Dumontet et al. 2025: Supplementary Figure 3

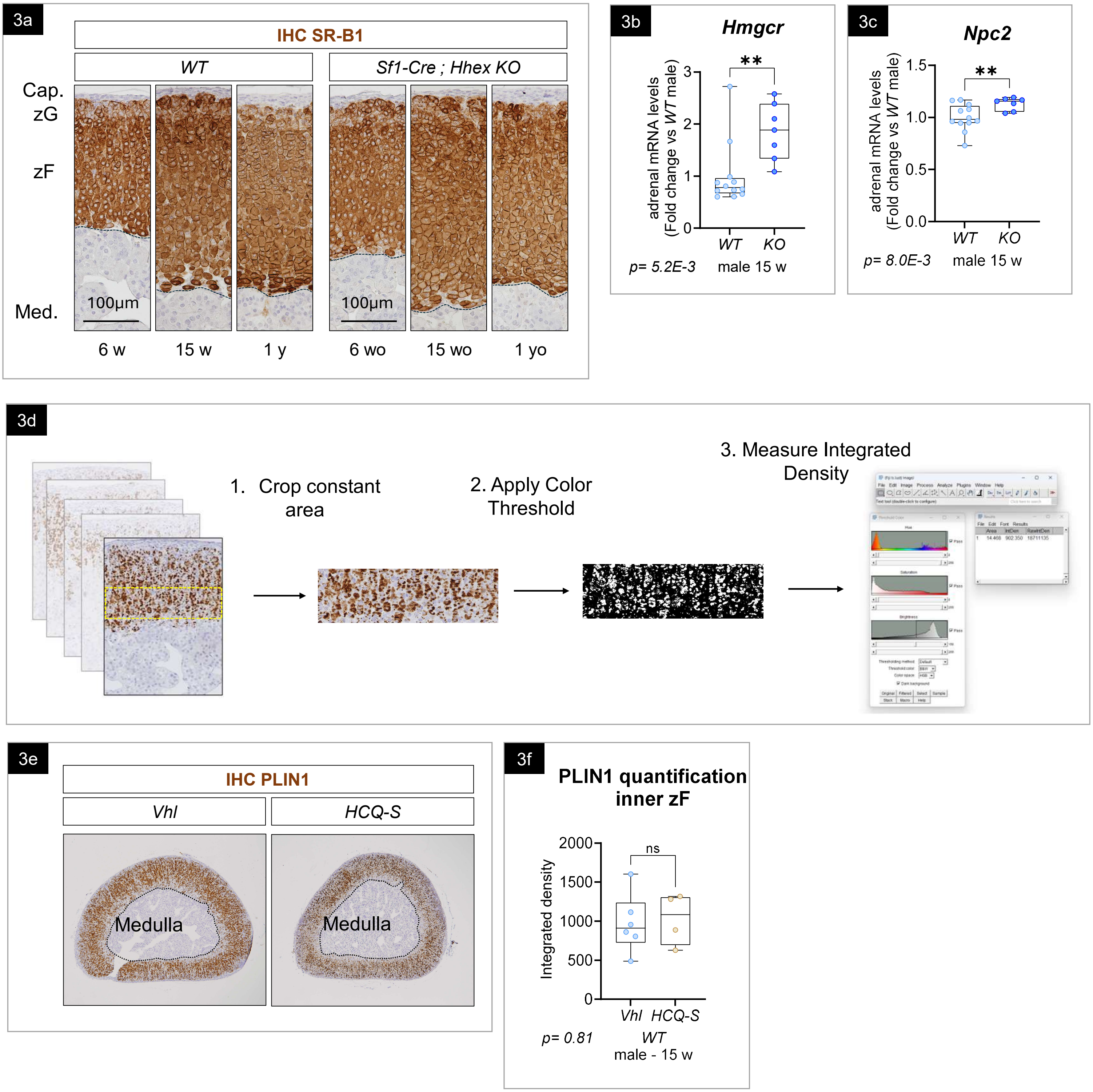

Supplementary Figure 3: (**a**) SR-B1 immunohistochemistry (IHC) in adrenals of *WT* and *Sf1-Cre ; Hhex KO* adrenals at 6 weeks, 15 weeks and over 1 year old. Nuclei were stained in blue with hematoxylin. Dotted lines represent the corticomedullary junction. *WT*: Wild Type, *KO*: Knockout, Cap.: Capsule, zG: zona glomerulosa, zF: zona fasciculata, Med.: medulla. (**b**) Quantification of *Hmgcr* transcripts by RT-qPCR in 15-week-old *WT* (n=12) and *SF1-Cre ; Hhex KO* (n=7) male adrenals. Graph represents box plots with individual biological replicates. *p*-value (*p*) was calculated using a Mann-Whitney test. (**c**) Quantification of *Npc2* transcripts by RT-qPCR in 15-week-old *WT* (n=12) and *SF1-Cre ; Hhex KO* (n=7) male adrenals. Graph represents box plots with individual biological replicates. *p*-value (*p*) was calculated using a two-tailed unpaired t-test with Welch's correction. (**d**) Schematic depicting the workflow used to quantify PLIN1 staining using imageJ. (**e**) PLIN1 immunohistochemistry (IHC) in adrenals of *WT*, Vehicle and hydroxychloroquine sulfate-treated (HCQ-S) 15-week-old male mice. Nuclei were stained in blue with hematoxylin. Dotted lines represent the corticomedullary junction. WT: Wild Type, KO: Knockout, Cap.: Capsule, zG: zona glomerulosa, zF: zona fasciculata, Med.: medulla. (**f**) Quantification of PLIN1 immunohistochemistry in the inner zF using imageJ. Graph represents box plots with individual biological replicates. Vehicle n=6, HCQ-s n=4. *p*-value (*p*) was calculated using a two-tailed unpaired t-test with Welch's correction.

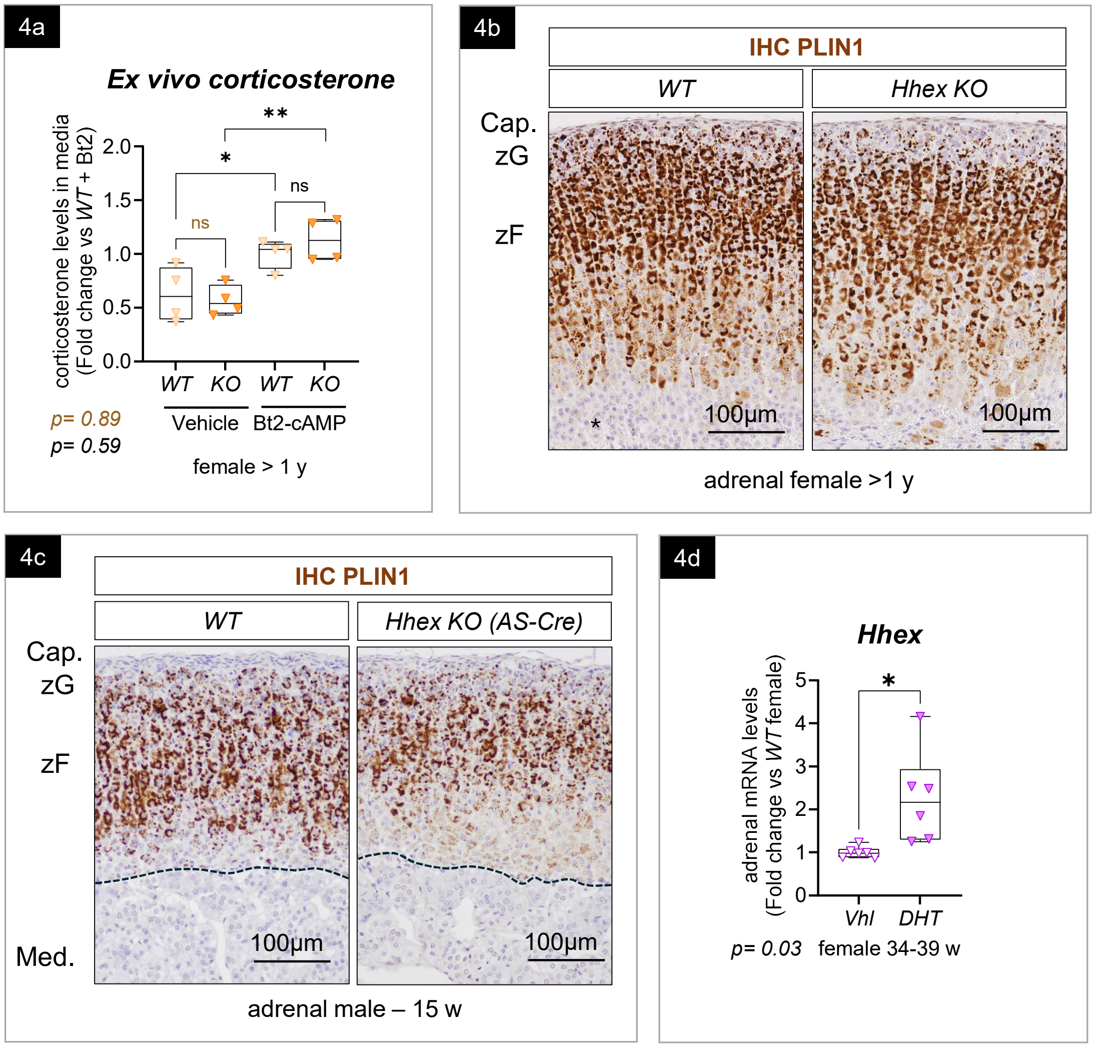

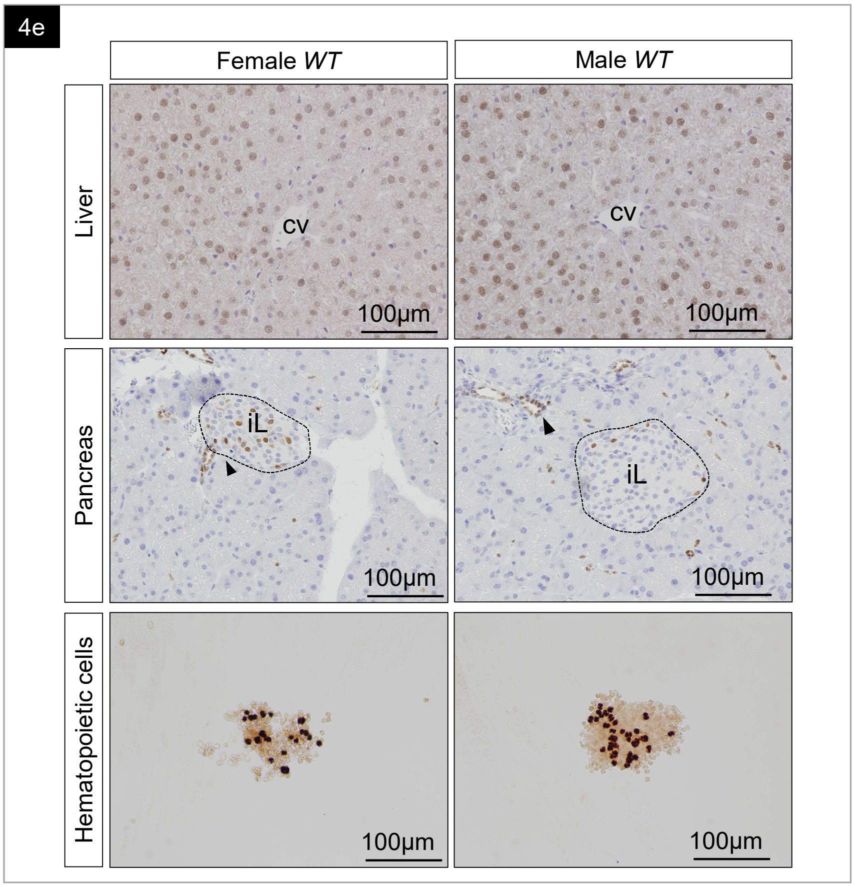

Supplementary Figure 4: (**a**) *Ex vivo* corticosterone production, released in culture media by adrenal explants after 2.5 hours incubation with 2.5mM of Bt2. *WT* (n=4) and *SF1-Cre ; Hhex KO* females (n=7). Graph represents box plots with individual biological replicates. *p*-value (*p*) was calculated using a 2-way ANOVA followed by a Šídák's multiple comparisons test. (**b**) PLIN1 immunohistochemistry in primiparous female. Nuclei were stained in blue with hematoxylin. Dotted lines represent the corticomedullary junction. *WT*: Wild Type, *KO*: Knockout, Cap.: Capsule, zG: zona glomerulosa, zF: zona fasciculata. *medulla cells were mostly absent from the images. (**c**) PLIN1 immunohistochemistry in *Cyp11b2-Cre ; Hhex KO*. Nuclei were stained in blue with hematoxylin. Dotted lines represent the corticomedullary junction. *WT*: Wild Type, *KO*: Knockout, Cap.: Capsule, zG: zona glomerulosa, zF: zona fasciculata, Med.: medulla. (**d**) *Ex vivo Hhex* expression by RT-qPCR in female adrenal explants treated for 48 hours with Vehicle (Vhl) or dihydrotestosterone (DHT) (0.1µM). (**e**) HHEX expression by immunohistochemistry (IHC) in 6-week-old female and male liver, pancreas and hematopoietic cells. cv: central vein, iL: islet of Langherans, arrowheads: endothelial cells.

Dumontet et al. 2025: Supplementary Figure 5

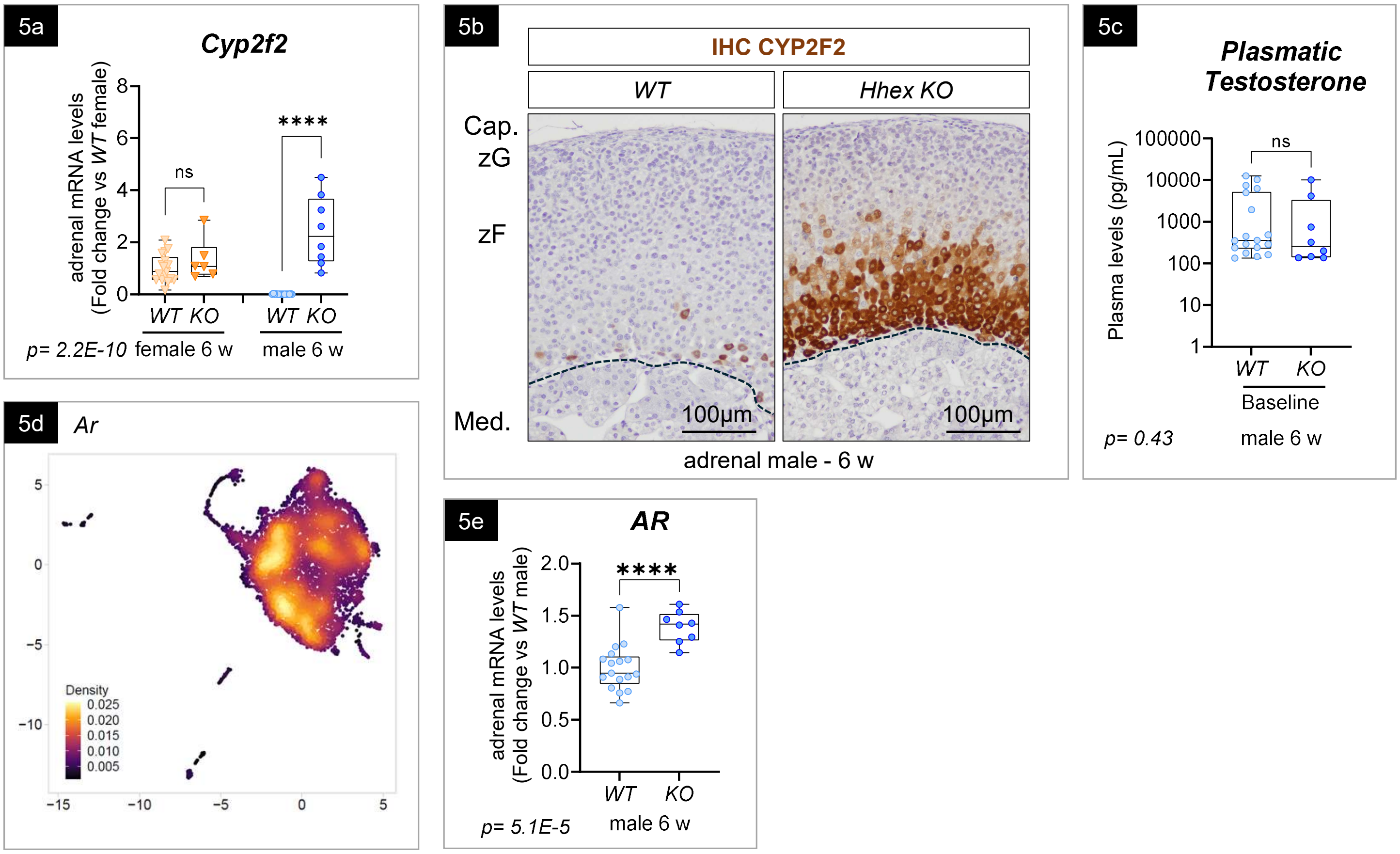

Supplementary Figure 5: (**a**) *Cyp2f2* expression by RT-qPCR in the adrenal gland of 6-week-old female (▽) and male (○), *WT* (n= 10 & 12) and *SF1-Cre ; Hhex KO* (n= 6 & 8). Graph represents box plots with individual biological replicates. *p*-value (*p*) was calculated using a 2-way ANOVA followed by a Šídák's multiple comparisons test. (**b**) CYP2F2 expression by immunohistochemistry (IHC) in adrenals of *WT* and *SF1-Cre ; Hhex KO* 6-week-old male mice. Nuclei were stained in blue with hematoxylin. Dotted lines represent the corticomedullary junction. *WT*: Wild Type, *KO*: Knockout, Cap.: Capsule, zG: zona glomerulosa, zF: zona fasciculata, Med.: medulla. (**c**) Testosterone plasma levels in 6-week-old *WT* (n=18) and SF1-Cre ; Hhex KO (n=8) male mice. Graph represents box plots with individual biological replicates. *p*-value (*p*) was calculated using a Mann-Whitney test. (**d**) UMAP representation of *Ar* expression in the scRNA-seq data set. (**e**) *Ar* expression by RT-qPCR in 6-week-old *WT* (n= 17) and *SF1-Cre ; Hhex KO* (n=8) male adrenals. Graph represents box plots with individual biological replicates. *p*-value (*p*) was calculated using a two-tailed unpaired t-test with Welch's correction. (**f**) List of genes commonly differentially expressed in 6-week-old *Hhex KO* males and 25-week-old *ARKO* male mice compared to their respective *WT*. Related to Figure 5J

Dumontet et al. 2025: Supplementary Figure 5

5f

Upregulated (674) in both KO by alphabetical order							Downregulated (562) in both KO by alphabetical order					
0610031O16Rik	Ccno	Ephb6	Hsd3b1	Ndufb5	Psmd12	Surf4	1520401A03Rik	Chst10	Gipc3	Man1c1	Ptgr1	Tbc1d16
0610038B21Rik	Cd1d1	Epop	Hspa12a	Ndufv3	Psme1	Suz12	4632428C04Rik	Chst12	Gja4	Map11	Ptprf	Tbc1d2
1110008P14Rik	Cd320	Etfa	Ibtk	Neb	Psme4	Svs1	6430584L05Rik	Cib2	Gjc1	Map3k9	Ptrh1	Tdrkh
1600014C10Rik	Cd36	Etnk1	Icosl	Nfia	Pter	Tacr2	9530077C05Rik	Ckb	Gjc2	Map7d1	Pxn	Tead3
1700028E10Rik	Cd47	Exoc4	Idh1	Nfyb	Ptgs2os2	Taf4b	A030001D20Rik	Cklf	Glrb	Mast4	Rab11fip1	Tent5b
1700030C10Rik	Cdc45	Exoc6	Idi1	Nipsnap3b	Ptp4a1	Tbrg1	A4galt	Clba1	Glt8d2	Mchr1	Rab11fip4	Tgfbr2
1700125H20Rik	Cdh2	Exosc5	Il22ra1	Nit2	Pvr	Tcf24	A830082N09Rik	Clic1	Gm10406	Medag	Rab3il1	Them6
2300009A05Rik	Cdh9	Exosc8	Il4ra	Nop10	Rab2a	Tef	AA986860	Cltb	Gm10575	Mettl26	Rab4a	Tie1
2610028E06Rik	Cdhr5	Fam110b	Inca1	Nqo2	Rab32	Thrb	Abca8b	Cmtm3	Gm19461	Mfsd13a	Rab7b	Timp1
3110045C21Rik	Cdk2ap2	Fam114a2	Insc	Nr0b1	Rab33a	Thrsp	Abcc8	Cnnm2	Gm31333	Mgat3	Rab8b	Tjp2
4930486L24Rik	Cdk6	Fam120a	Insr	Nr1d2	Rab5if	Timm10b	Abr	Cnp	Gm5134	Mical2	Rabep1	Tk2
6430548M08Rik	Cdk8	Fam126a	Ireb2	Nr5a1	Rab9	Timm23	Acadvl	Cnrip1	Gm5148	Mpp3	Rad9a	Tle2
9530062K07Rik	Cdkl5	Fam169a	Isca2	Nrep	Rad54l2	Timm8b	Acan	Col16a1	Gm826	Mrgpre	Rapsn	Tle5
A830035O19Rik	Cdv3	Fam210a	Iscu	Nsdhl	Rapgef4	Tlcd1	Acap3	Col8a1	Gm9767	Mrps11	Rarg	Tle6
Aass	Cdyl	Fam32a	Isoc2a	Nudcd2	Rassf8	Tmbim6	Acer2	Coro1c	Gmip	Mtarc2	Rasgrf1	Tmcc1
Abca1	Cebpb	Fat4	Isoc2b	Nudt10	Rbbp8	Tmed3	Ada	Cpm	Gmnn	Mtmr11	Rasl12	Tmem106a
Abca4	Cert1	Fbxo7	Ivd	Nudt11	Rcl1	Tmem135	Adcy3	Cpne2	Gnaz	Mutyh	Rasl2-9	Tmem200a
Abhd10	Cgrrf1	Fbxw11	Jarid2	Nudt12	Rcn1	Tmem139	Adcy7	Creb3l1	Gnb4	Mxra7	Rbpms2	Tmem200c
Abra	Chpt1	Fdft1	Jtb	Nudt8	Rdh1	Tmem141	Add1	Crlf2	Gnb5	Myl6b	Reck	Tmem237
Acad11	Ciapin1	Fdps	Kcns3	Nup188	Retreg1	Tmem167	Adgrg1	Crtc1	Gngt2	Myo18a	Relb	Tmem37
Acadsb	Cib4	Fer	Kif5b	Obp2a	Rfk	Tmem170b	Adra1d	Crym	Gnpda1	Myo1a	Rem1	Tmem51
Acat1	Cisd1	Fetub	Kitl	Ociad2	Rfx7	Tmem178b	Adssl1	Csf1	Gpr137b	Nags	Rep15	Tmem8b
Acbd5	Cisd2	Fggy	Klhl24	Olfr1034	Rgs12	Tmem185a	Afmid	Cspg4	Gpr18	Nans	Rftn2	Tmod1
Aco1	Cited2	Fhdc1	Kmt5a	Olfr446	Rgs2	Tmem29	Agpat4	Csrp2	Gpsm3	Ncapd2	Rfx1	Tnfaip2
Aco2	Cited4	Filip1	Knop1	Opa1	Rhod	Tmem33	Aif1	Ctsz	Gpx8	Ndufs2	Rgs3	Tnfrsf11a
Acox1	Clint1	Fitm1	Kyat3	Osbpl6	Rims4	Tmem38b	Akr1b10	Ctxn3	Grb14	Nectin1	Rhbdf1	Tnfrsf23
Acsl1	Clpb	Fkbp4	L3hypdh	Osr2	Rnf180	Tmem50a	Alad	Cxxc5	Greb1	Nectin2	Rhbdl3	Tnfsf12
Acsm3	Cmas	Fktn	Larp1b	Oxtr	Rnf181	Tmem62	Alox12	Cyba	Gria3	Nelfa	Rhcg	Tnfsfm13
Acss3	Cmpk1	Fndc10	Lars	Pag1	Rnf5	Tmprss6	Alpl	Cygb	Grin2c	Neo1	Rhof	Tns3
Adamts3	Cnih1	Fndc5	Ldhd	Paics	Rnf6	Tomm20	Amigo3	Cyp11a1	Grk5	Nfatc2	Rilpl1	Tox2
Adh1	Cops5	Frmd5	Lims1	Pakap	Rnmt	Top1mt	Angpt2	Cyp4f16	Grrp1	Nfx1	Rin3	Tpm2
Adh5	Coq6	Frzb	Lipa	Pank1	Rpl7a-ps4	Tor1aip2	Angpt4	Cytl1	Grtp1	Niban2	Ripor1	Trank1
Adh7	Coq8a	Fscn1	Lmo4	Panx3	Rps27l	Tpsb2	Angptl2	D11Wsu47e	Gsdme	Nol4l	Ripply3	Treml2
Adi1	Cox14	Fv1	Lmod2	Paox	Rtkn	Traf4	Ank1	D630003M21Rik	Gstt1	Npr2	Rnaset2b	Trib2
Adipor2	Cox18	Fxn	Lpgat1	Paqr9	Rtn4	Trappc8	Ankrd44	Dbn1	H1f0	Npr3	Rnd3	Trip6
Aen	Cox6a1	Fzd4	Lpin3	Parg	Samd5	Trim12c	Ap2m1	Ddah2	H2aw	Nr1h3	Rnf227	Tshz2
Aff2	Cox7a2	G2e3	Lratd1	Parm1	Sbk1	Trp53inp2	Apln	Ddr1	H2-Eb1	Nsmce3	Rnf24	Tspan11
Agt	Cox7b	Gabarapl2	Lrpprc	Parp1	Sccpdh	Tspan12	Aprt	Defb25	Hdac1	Nsmf	Rpusd4	Ttll1
Agxt2	Cox7c	Gabrg3	Lrrc57	Pccb	Scfd1	Tstd3	Arhgap15	Denn2b	Hdac11	Nsun5	Rrad	Ttll12
Ahcyl2	Cpeb3	Gadl1	Lrrk2	Pcgf5	Sdhaf4	Ttll2	Arhgap44	Dennd2a	Hdac6	Nt5e	Rtn4rl1	Ttll7
Ajap1	Cpt1a	Galnt7	Lxn	Pcna	Sec22a	Ttpa	Arhgef10	Dio3	Hdhd3	Oaf	Rubcnl	Tubb3
Ak3	Cracdl	Gan	Ly6h	Pcx	Sec22b	Txndc2	Arhgef10l	Dmc1	Heyl	Odf3	Rufy1	Tubg2
Akr1c18	Cript	Gbe1	Macrod1	Pde1a	Sec24d	Txndc5	Arhgef19	Doc2b	Hmgb3	Olfm1	S1pr1	Txn2
Akr1cl	Cry2	Gclc	Man1a	Pde7a	Serf1	Txnl1	Arl2	Dusp14	Hmox1	Olfml2a	Scarb1	Uaca
Akr1d1	Cse1l	Gcnt4	Man2b1	Pde8b	Serpina3c	Ube2k	Arl3	Dyrk3	Hnrnph3	Olfr1396	Sec62	Unkl
Aldh2	Cst8	Gfus	Map1lc3a	Pdgfd	Serpinb1a	Ubqln2	Armc2	Ece2	Hsd3b6	Olfr330	Selenon	Ush1g
Aldh3b1	Ctso	Ggct	Map6d1	Pdhb	Sertad4	Ubxn2a	Armcx2	Ecrg4	Hspb1	Optc	Selenow	Usp21
Aldh7a1	Cul3	Ggh	Mapt	Pdhx	Sestd1	Uck2	Arsa	Efemp2	Hspg2	Osbp2	Sema3g	Vav2
Aldh9a1	Cyp11b1	Ggt6	Marchf2	Pecr	Sfxn1	Ugt1a1	Art2b	Efna1	Htr3a	Osbpl3	Sema5b	Vcl
Aldoc	Cyp21a1	Glrx5	Marchf5	Pex13	Sgms1	Umodl1	As3mt	Efna4	Htra3	Osbpl5	Sema7a	Vcpkmt
Amd1	Cyp2b10	Gm13010	Mbd2	Pex16	Sh2d4a	Unc93a2	Asb16	Efnb2	Icam4	Osgin1	Septin6	Vdr
Amn1	Cyp2c23	Gm13212	Med1	Pex19	Sh3bgr	Uso1	Atg16l2	Ehd2	Idh2	Osmr	Serinc5	Vps26c
Amy1	Cyp2f2	Gm16630	Megf9	Pex5	Sh3glb1	Vapa	Atp1b2	Ehd4	Ifitm2	Otub2	Serpina6	Vsig10
Angptl1	Cyp51	Gm18726	Mettl7a1	Pgap2	Shb	Vegfd	Atp4a	Elmo2	Igdcc4	P2rx6	Sh3bgrl3	Wfdc6a
Ankdd1b	Cyth3	Gm19522	Mettl9	Pgm1	Shmt1	Vldlr	Atp6v1c2	Eml1	Igfbp5	P2ry2	Sh3gl2	Wfdc8
Ankrd35	D16Ertd472e	Gm19689	Mexis	Pgm3	Sidt2	Vmn1r79	Atp9a	Emp3	Igsf3	P3h4	Shh	Zcchc17
Ankrd46	Dbp	Gm2098	Mfn2	Pgrmc1	Sin3b	Vmp1	Atxn7l2	Enpep	Il17rd	Pafah1b3	Shmt2	Zdhhc8
Apip	Dcaf12	Gm2694	Mfsd14a	Phldb2	Sinhcaf	Vnn1	AU021092	Ephx1	Inf2	Pafah2	Sipa1	Zfp251
Apoc4	Dcaf17	Gm31235	Mfsd4b3-ps	Piga	Slc14a2	Vnn3	B4galnt3	Eppin	Inpp4a	Palld	Slc12a2	Zfp423
Apof	Dcun1d3	Gm36201	Mgst1	Pigk	Slc17a6	Vps37c	Baiap2	Eps8	Iqcg	Palm3	Slc12a4	Zfp618
Apol6	Dcun1d4	Gm4013	Mia2	Pik3c2g	Slc24a3	Vsig8	Batf3	Erich6	Itpk1	Palmd	Slc16a14	Zfp647
Appl2	Dcxr	Gm49083	Micos13	Pkdcc	Slc25a12	Wdr61	Bbs4	Esam	Itpr3	Paqr4	Slc16a2	Zfp960
Arhgap42	Ddx4	Gm7463	Mir6358	Pla1a	Slc25a33	Wfdc12	Bcam	Etnk2	Jsrp1	Parp3	Slc25a37	
Armcx5	Deptor	Gm973	Mkln1	Pla2g12a	Slc25a5	Xdh	Bcl11b	Evc	Jun	Pbxip1	Slc27a3	
Arrdc4	Dera	Golga7	Mlkl	Pla2g4d	Slc29a3	Xpnpep1	Bcr	Evc2	Kazald1	Pde4a	Slc29a4	
Asap2	Derl2	Gpam	Mme	Plekha3	Slc31a1	Xrcc6	Bfsp1	Exoc3l	Kcnab3	Pde5a	Slc2a3	
Ascc3	Desi1	Gpbp1l1	Mob4	Plod2	Slc33a1	Xxylt1	Bik	Exoc3l2	Kcnd3	Pdgfa	Slc2a4	
Asgr1	Desi2	Gpc4	Mocs1	Plpp3	Slc35f3	Ykt6	Blnk	Extl1	Kcnj4	Pdgfrl	Slc30a2	
Asgr2	Dhcr24	Gpc5	Mospd2	Plpp6	Slc38a9	Zfp185	Bspry	F2	Kcnj5	Pdia5	Slc35e4	
Asl	Dhdh	Gpt2	Mpnd	Pm20d2	Slc4a7	Zfp51	Btg1c	Faah	Kcnk9	Pdlim7	Slc44a2	
Asns	Dhrs3	Gpx1	Mr1	Pmm1	Slc7a6	Zfp91	C1ra	Fam181b	Kcnma1	Pea15a	Slc49a4	
Atf6	Dhx40	Gramd1b	Mrpl20	Polk	Slc7a8	Zhx3	C1s1	Fam187b	Kcnmb4	Peg13	Slc6a8	
Atl2	Dmd	Grb10	Mrpl53	Polr1d	Slfn8	Znrf2	Cables1	Fat3	Kcnq1	Pfn2	Smad3	
Atox1	Dmrta1	Greb1l	Mrpl57	Pon2	Slfn9	Zswim7	Cacng4	Fbxo10	Kdelr3	Pgghg	Smagp	
Atp2a2	Dnajb11	Grsf1	Mrps24	Pon3	Smad2		Cad	Fdxr	Khdrbs3	Pgm2	Smarcd3	
Atp5g3	Dock1	Gspt1	Mrps28	Pou4f1	Smad9		Calhm2	Fgd5	Kif19a	Phldb1	Smn1	
Atp5j	Dock8	Gsta3	Mrps34	Ppa1	Smap1		Camk1d	Fgf11	Kiz	Pik3ap1	Smtn	
Atp5md	Dpm1	Gstk1	Ms4a10	Ppat	Smim10l1		Camsap3	Fhl2	Klc1	Pitpnm3	Snn	
Avpi1	Dpy19l1	Gstp1	Msh3	Ppp1cb	Smim26		Capn2	Filip1l	Klc3	Pkia	Snx20	
B4galt1	Dpy30	Gtf2b	Msmo1	Ppp1r14c	Snap23		Car7	Fkbp10	Klhdc7a	Plcb3	Sorbs2	
Bach1	Dpyd	Gtf3c3	Mtap	Ppp2ca	Snhg6		Card14	Flnb	Klhl3	Plcd1	Sort1	
Bag2	Dsp	Hacd1	Mtfr1	Ppp2cb	Snrpg		Catsper2	Flrt1	Lama5	Plcd3	Spag4	
Bcl7c	Dtd2	Hacd2	Mthfd1	Ppp2r1a	Sod1		Cbfb	Flt1	Layn	Plcl1	Spry1	
Bend7	Dusp22	Haus2	Mtm1	Ppp2r3a	Sod2		Cc2d1a	Flt4	Lgals1	Plekha6	Spsb2	
Bet1	Dynlt3	Hbp1	Mtrex	Ppp3ca	Speer9-ps1		Ccdc102a	Fmnl2	Lhfp	Plekhg2	Sptan1	
Bin3	E2f3	Hdac4	Mtx2	Pqlc3	Spg20		Ccdc107	Fmnl3	Lifr	Plekhm2	Src	
Bmyc	Echdc1	Hdlbp	Mvd	Prdx6	Spink4		Ccdc114	Fnbp1l	Lin37	Plxdc1	Srcin1	
Bnip3	Echdc3	Heca	Mxi1	Prelid2	Spint2		Ccdc153	Foxo6	Lin7b	Plxnc1	Srd5a2	
Boc	Echs1	Helt	Myc	Prkaa2	Sptlc2		Ccdc30	Foxred2	Llgl1	Podxl2	Srgap3	
Bola3	Egfl6	Hint3	N4bp2l1	Prkar2a	Srd5a1		Ccdc8	Frmd4a	Loxl3	Ppfibp1	Ssbp3	
Bphl	Ehf	Hmgcr	Naa60	Prkcd	Srp54c		Cccdc88b	Frmd6	Lpar1	Ppil1	St6galnac2	
Cadps2	Eif3j1	Hmgcs1	Nampt	Prlr	Ssr2		Ccl19	Fstl3	Lrp5	Praf2	St6galnac4	
Car8	Eif4g3	Hnmt	Narf	Prob1	Ssu72		Ccnd1	Fzd5	Lrrc1	Prima1	St8sia5	
Carhsp1	Elfn1	Hnrnpll	Nat8f1	Prodh	St3gal1		Cd24a	Fzd9	Lrrk1	Prkcb	Stk10	
Casp3	Ell2	Hoxb5	Nceh1	Proser2	Stard4		Cd300lg	Gad1	Lrrn2	Prmt2	Strc	
Cat	Elmo1	Hoxb6	Ncoa4	Prpf18	Stard5		Cd63	Gadd45gip1	Lrrn4	Prnp	Sulf2	
Cbln1	Emb	Hoxb7	Ndufa2	Prr16	Stx17		Cd74	Gas5	Lsr	Prrg4	Supt5	
Cbr1	Emc6	Hrg	Ndufa4	Prxl2b	Stxbp3		Cdc42ep1	Gata1	Ly6d	Prss35	Syde1	
Cbr2	Emc9	Hs3st1	Ndufa6	Psd3	Sub1		Cdc42ep2	Ggcx	Lynx1	Prss53	Synj2	
Ccng1	En2	Hsd17b7	Ndufa7	Psmb5	Sugct		Cercam	Ggt5	Mad1l1	Ptgis	Synpo	

Dumontet et al. 2025: Supplementary Figure 6

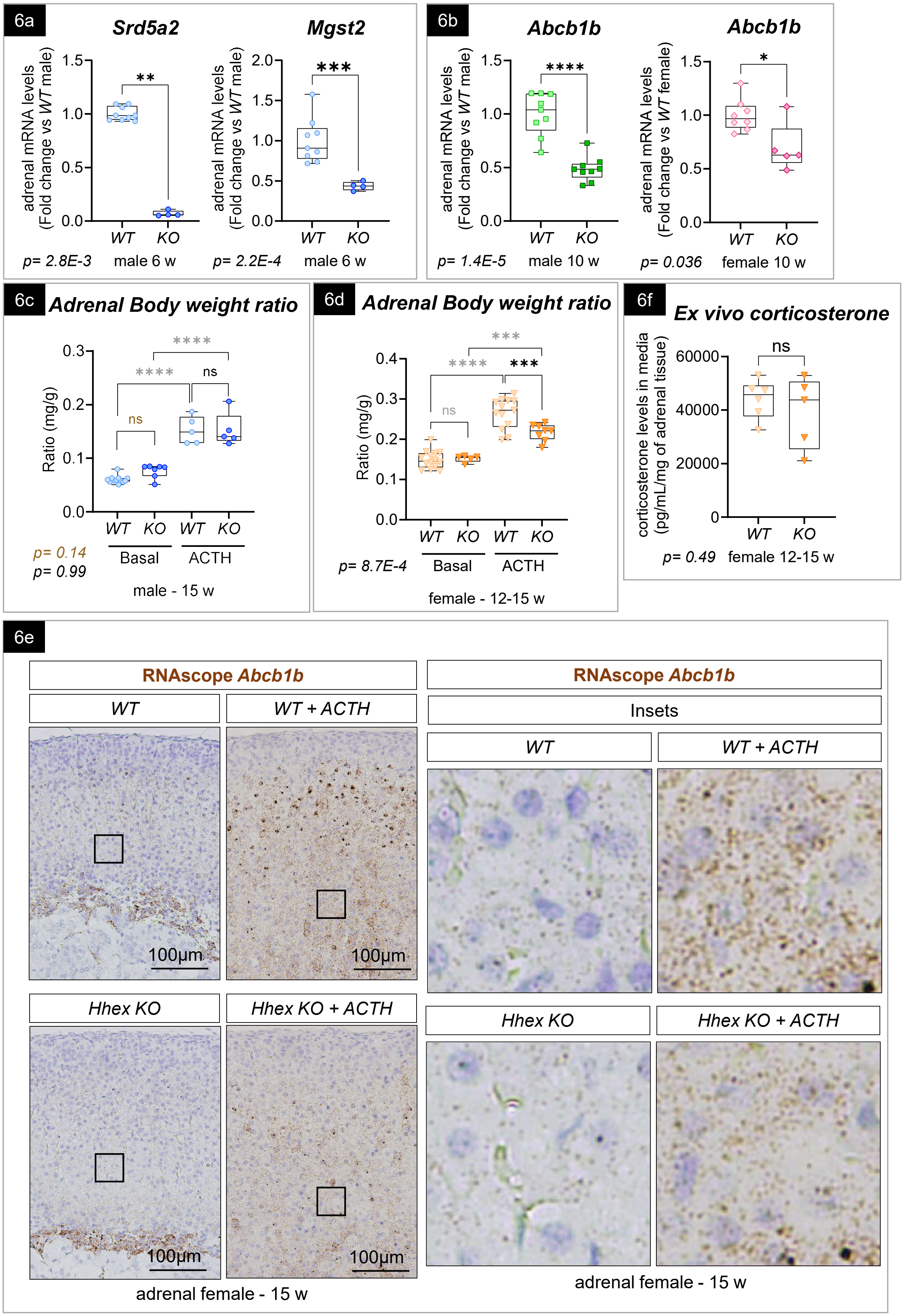

Supplementary Figure 6: (**a**) Quantification of *Srd5a2* and *Mgst2* transcripts by RT-qPCR in 6-week-old *WT* (n=9) and *SF1-Cre ; Hhex KO* (n=4) male adrenals. Graph represents box plots with individual biological replicates. *p*-value (*p*) was calculated using a Mann-Whitney (*Srd5a2*) and a two-tailed unpaired t-test with Welch's correction (*Mgst2*). (**b**) Quantification of *Abcb1b* transcripts by RT-qPCR in 10-week-old *WT* (n=17 and 17) and *Cyp11b1-Cre*^{ERT2} *Hhex KO* (n=8 and 6) male (□) and female (◊) adrenals. Graph represents box plots with individual biological replicates. *p*-value (*p*) is calculated using a two-tailed unpaired t-test with Welch's correction. (**c-d**) Adrenal (left gland) body weight ratio of male (**c**) and female (**d**) mice at baseline and after chronic ACTH administration. Ns: non-significant. A complete list of p-values is provided in the supplementary table related to Statistics. (**e**) *Abcb1* expression in 15-week-old *WT* and *SF1-Cre ; Hhex KO* female adrenals by RNAscope, at baseline and after chronic stress. Nuclei are stained in blue with hematoxylin. Dotted lines represent the corticomedullary junction. *WT*: Wild Type, *KO*: Knockout. (**f**) *Ex vivo* corticosterone production, released in culture media for 6 hours by adrenal explants from female after chronic ACTH administration. *WT* (n=6) and *SF1-Cre ; Hhex KO* (n=5). Graph represents box plots with individual biological replicates. *p*-value (*p*) was calculated using a two-tailed unpaired t-test with Welch's correction.
